# Prediction of Passive Membrane Permeability by Semi‐Empirical Method Considering Viscous and Inertial Resistances and Different Rates of Conformational Change and Diffusion

**DOI:** 10.1002/minf.201900071

**Published:** 2019-10-14

**Authors:** Yoshifumi Fukunishi, Tadaaki Mashimo, Takashi Kurosawa, Yoshinori Wakabayashi, Hironori K. Nakamura, Koh Takeuchi

**Affiliations:** ^1^ Molecular Profiling Research Center for Drug Discovery (molprof) National Institute of Advanced Industrial Science and Technology (AIST) 2-3-26, Aomi, Koto-ku Tokyo 135-0064 Japan; ^2^ Technology Research Association for Next-Generation Natural Products Chemistry 2-3-26, Aomi, Koto-ku Tokyo 135-0064 Japan; ^3^ IMSBIO Co., Ltd. Owl Tower, 4–21-1, Higashi-Ikebukuro, Toshima-ku Tokyo 170-0013 Japan; ^4^ Hitachi Solutions East Japan, 12–1 Ekimaehoncho, Kawasaki-ku, Kawasaki Kanagawa 210-0007 Japan; ^5^ BY-HEX LLP, 1–19-14, Shimizu, Suginami-ku Tokyo 167-0033 Japan; ^6^ Biomodeling Research Co., Ltd. 1-704-2 Uedanishi, Tenpaku-ku, Nagoya Aichi 468-0058 Japan

**Keywords:** PAMPA, Fick's law, QSPR, regression model, middle molecule

## Abstract

Membrane permeability is an important property of drugs in adsorption. Many prediction methods work well for small molecules, but the prediction of middle‐molecule permeability is still difficult. In the present study, we modified a classical permeability model based on Fick's law to study passive membrane permeability. The model consisted of the distribution of solute from water to membrane and the diffusion of solute in each solvent. The diffusion coefficient is the inverse of the resistance, and we examined the inertial resistance in addition to the viscous resistance, the latter of which has been widely used in permeability prediction. Also, we examined three models changing the balance between the diffusion of solute in membrane and the conformational change of solute. The inertial resistance improved the prediction results in addition to the viscous resistance. The models worked well not only for small molecules but also for middle molecules, whose structures have more conformational freedom.

## Graphical abstract

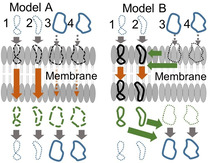

## 1 Introduction

Permeability is one of the most important factors in a drug's adsorption and target‐binding properties in cells. The understanding and predicting membrane permeability of molecules have been studied for last few decades. It is still one of the hot topics, especially under circumstances where the molecular weights of drug molecules have been increasing and larger molecules often face the lower permeability than smaller drug molecules do. There have been a number of reports on permeability.^1^, ^2^, ^3^, ^4^, ^5^, ^6^, ^7^, ^8^, ^9^, ^10^, ^11^, ^12^, ^13^, ^14^, ^15^, ^16^, ^17^, ^18^, ^19^, ^20^, ^21^, ^22^, ^23^, ^24^, ^25^, ^26^ The main permeability problems are adsorption in human intestine, extraction from kidney, penetration of the blood‐brain barrier, skin permeability, and the permeability of the cell membrane to approach target proteins in cells. Caco‐2 cells and MDCK cell systems are two of the model systems that mimic human intestine adsorption and extraction from kidney, respectively. Parallel artificial membrane permeability assay (PAMPA) systems have been popular in vitro assay systems for the past 20 years.^26^, ^27^, ^28^, ^29^, ^30^, ^31^, ^32^ PAMPA systems have been improved to mimic in vivo permeability by trying various membrane materials, pH levels of donor and acceptor liquids and the other conditions. Certain mechanisms underlie permeability.^2^, ^3^, ^26^ Namely, solute molecules penetrate the cell membrane by diffusion (transcellular), the solute molecules go through the tight junction (paracellular), and transporters and channel proteins work in the influx and efflux processes. Among these mechanisms, PAMPA permeability represents transcellular passive permeability.

Recent advances in molecular dynamics (MD) simulations enable the understanding and evaluation of transcellular passive permeability.^4^, ^5^, ^6^, ^7^, ^8^, ^9^, ^10^, ^11^, ^12^, ^13^, ^14^, ^15^ In these calculations, MD simulations have been applied to explicit atomic models of membrane molecules with solvent water molecules. Since permeation is a very slow process, biased MD simulations have been popular in this analysis. MD simulations have shown that the distribution of the existence probability of solute and the diffusion of solute in the membrane gave the permeability constant.

On the other hand, many approaches have adopted quantitative structure‐property relationship (QSPR) models for passive permeability based on Fick's law.^2^, ^3^, ^26^ Previous works have shown the efficiency of this basic theory, and some extensions from this theory have improved prediction accuracy.^16^, ^17^ Fick's law explains that permeability is a combination of the transfer of solutes from the donor water into the membrane and the diffusion of the solutes from the donor to the acceptor sides in the membrane. Only the neutral molecule moves into the lipid layer so that the p*K*_a_ and the partition coefficient (Log*P*) or the distribution coefficient (Log*D*) determine the distribution of solute between the donor water and the membrane. The permeability *P*_app_ is given as follows.

(1) $$LogP{}_{app}=Log\frac{D\cdot M}{h}$$

where *P*_app_, *D*, *M*, and *h* represent the apparent membrane permeability, distribution coefficient, diffusion constant of the solute, and thickness of the membrane, respectively. The above MD simulations support this assumption. Namely, Log*D* corresponds to the probability distribution of existence and *M* corresponds to the diffusion of solute obtained by the MD simulation, respectively.

The diffusion constant *M* is estimated by the Einstein‐Stokes relation

(2) $$M=\frac{k_{B}\cdot T}{6\pi \cdot \mu \cdot R}$$

where *k*_B_, *T*, *μ*, and *R* are the Boltzmann constant, temperature, viscosity and radius of the solute, respectively. Since Log*D* corresponds to the free energy of the transfer from water to membrane, this value could be approximated by the accessible surface area (ASA) method, the generalized Born (GB) method, polar surface area (PSA), and so on.^33^ Thus, the following linear regression model can estimate Log*P*_app_.

(3) $$LogP{}_{app}=LogD-LogR+c_{0}=\sum_{i=1}^{N_{descriptor}}c_{1}^{i}x_{i}+c_{0}$$

where *x*, *c*, and *N*_descriptor_ are the molecular descriptors, the fitting parameters, and the total number of descriptors, respectively, and the fitting parameters represent the characteristic properties of the membrane.

Recent advances in pharmaceutical research have increased the molecular size of drugs, and middle‐molecule drugs, with molecular masses >500 Da, have become popular. In the last few decades, the major drug targets have been receptors and enzymes. Pharmaceutical companies have released several thousands of drug molecules; however, they are now facing a severe depletion in the druggable targets with conventional strategies. Research activities focusing on protein‐protein interaction (PPI) inhibitors have been started instead of the projects on finding ligands to receptors and enzymes.^34^, ^35^ To inhibit PPIs, larger molecules are often preferable than the small molecules. However, the hydrophobic and often insoluble physical properties of middle molecules that are distinct from small ligands cause serious problems in their development stages.

While one of the major problems of middle molecules is the low membrane permeability, the synthetic accessibility in chemical modifications is also limited. The synthetic processes of middle molecules are more complicated and time‐consuming than that of small molecules. Thus, we need the mechanistic understandings to unveil the dominant factors of membrane permeability, rather than black‐box permeability predictions, to guide a rational modification of the middle molecules.

A number of reports suggested that conformational change is essential in permeability and cyclization as well as in the methylation of main‐chain amide groups, and some chemical modifications have improved permeability.^18^, ^19^, ^20^, ^21^, ^22^ Many studies have challenged this problem by using QSPR models and have successfully predicted membrane permeability, including that of middle molecules.^23^ However, some studies have suggested that it is still difficult to predict the membrane permeabilities of middle molecules.

**Figure 1:**
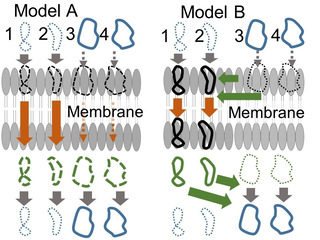
Permeability mechanism Models A and B. The rings represent the solute molecules and the different shapes represent the different conformers. Thick solid and thin dotted rings represent the large and small populations of molecules, respectively. The buffer solvent region is separated by the lipid bilayer in the middle (gray). Orange and green arrows represent the diffusion in the membrane and conformer change in the membrane depicted in gray. The open forms (3 and 4) are major rather than the closed form (1 and 2) in the buffer solvent. In Model A, there is no conformation change in the membrane. In Model B, the solute molecules in membrane show conformation change and the closed form becomes major.

Diffusion constant *M* in eq. 1 consists of viscous resistance and inertial resistance. Equation 2, which is the inverse of viscous resistance, assumes the slow migration of solute in equilibrium. Permeability is non‐equilibrium process in general, and the density of the solute keeps changing in the experiment. When solute molecules push solvent molecules, the inertial resistance is proportional to the cross section of the solute. In general, we have ignored inertial resistance without examining the actual experimental data. In a non‐equilibrium state, the density of solute in the membrane cannot reach equilibrium. The solute can pass through the membrane before the conformational ensemble of the solute reaches equilibrium distribution (see Figure 1A).^4^ In this case, the distribution of solute is different from the distribution coefficient *D* observed in equilibrium.

In the present study, we examined the diffusion process by using a classical QSPR model. We considered inertial resistance the same as viscous resistance. Also, we developed formulas considering the above two cases in which the conformational change occurs faster or slower than the diffusion in the membrane.^5^

## 2 Methods

We classify the membrane permeability process into two models according to the speed of permeability.^5^ If the membrane permeation is faster than the conformational change of the solute in the membrane, each conformer penetrates the membrane while keeping the 3D structure of each conformer, and the dominant conformer in water could be the main contributor to the permeability (permeability model A in Figure 1A). If the membrane permeation is slower than the conformational change of the solute in the membrane, the most stable conformer in the membrane could contribute to the permeability (permeability model B in Figure 1B). In this article, we apply Fick's law to membrane permeability, since the physical meaning of Fick's law is clear and simple.

### 2.1 Permeability Model A

Since each solute penetrates the membrane without conformational change, the distribution of conformers in the membrane is equal to that in water solvent. The total permeability ratio (*P*_app_^all^) is the summation of the permeability of all conformers.

(4) $$LogP_{app}^{all}=Log\left(\sum_{a=1}^{N_{conformer}}d_{wat}\left(a\right)P_{app}\left(a\right)\right)$$

Here, *P*_app_(*a*), *d*_wat_(*a*), and *N*_conformer_ are the apparent permeability of the *a*‐th conformer, the distribution of the *a*‐th conformer in water, and the total number of conformers, respectively. The following relation is obvious.

(5) $$P_{app}^{all}={\left\langle \frac{D(a)M(a)}{h}\right\rangle }_{wat}={\left\langle exp\left(log\frac{D(a)M(a)}{h}\right)\right\rangle }_{wat}\;={\left\langle exp\left(LogP_{app}\left(a\right)\cdot log10\right)\right\rangle }_{wat}$$

where the brackets < >_wat_ stand for the average over the distribution in water. The fraction of the a‐th conformer in water (=*d*_wat_(*a*)) is given by

(6) $$d_{wat}\left(a\right)=\frac{n\left(a\right)\exp(-E^{wat}\left(a\right)/\left(k_{B}T\right)}{\sum_{b=1}^{N_{conformer}}n\left(b\right)\exp(-E^{wat}\left(b\right)/\left(k_{B}T\right)}$$

where *E* ^wat^(*a*) and *n*(*a*) are the energy and the degeneracy of the *a*‐th conformer, respectively.

When we apply Kubo's cumulant expansion to eq. 5, the first two terms of the expansion are as follows.^36^

(7) $$LogP_{app}^{all}=<LogP_{app}\left(a\right){>}_{wat}-\frac{1}{2}\left\langle LogP_{app}{\left(a\right)}^{2}-{\left\langle LogP_{app}\left(a\right)\right\rangle }^{2}\right\rangle {}_{wat}+\frac{1}{6}\cdots$$

The first term is the average Log*P*_app_ and the second is the deviation of Log*P*_app_.

A linear regression model that is a weighted summation of the descriptor values approximates the Log*P*_app_ of the *a*‐th conformer as follows,

(8) $$LogP{}_{app}\left(a\right)=Log\frac{D(a)M(a)}{h}=\sum_{i=1}^{N_{descriptor}}c_{1}^{i}x_{i}\left(a\right)+c_{0}$$

where *c* and *x*(*a*) are the fitting parameters and the descriptor of the a‐th conformer, respectively.

If the descriptors *x*_A_ and *x*_B_ are independent from each other, <*x*_A_+*x*_B_>=<*x*_A_>+<*x*_B_> and also σ(*x*_A_+*x*_B_)=σ (*x*_A_)+σ (*x*_B_), where σ stands for the deviation. Thus, if all the descriptors are independent of each other, eq. 7 becomes as follows.

(9) $$LogP_{app}^{all}=c_{0}+\sum_{i=1}^{N_{descriptor}}c_{1}^{i}<x_{i}{>}_{wat}+\sum_{i=1}^{N_{descriptor}}c_{2}^{i}\sigma {\left(x_{i}\right)}_{wat}+\cdots$$

In permeability model A, the deviations of all the descriptors, including the ASA and the radius of the solute, contribute to Log*P*_app_^all^ in addition to the average values of these descriptors.

### 2.2 Permeability Model B

If the membrane penetration is much slower than the conformation change, the distribution of conformers reaches equilibrium in the membrane and in water. The distribution constant *D* is given by the partition function of the molecule as follows.

(10) $$LogD=Log\frac{Z^{oct}}{Z^{wat}}=Log\frac{\sum_{b=1}^{N_{conformer}}n\left(b\right)\cdot exp(-E{\left(b\right)}^{oct}/k_{B}T)}{\sum_{b=1}^{N_{conformer}}n\left(b\right)\cdot exp(-E{\left(b\right)}^{wat}/k_{B}T)}$$

where *Z*°^ct^, *Z* ^wat^, and *n*(*b*) are the partition functions in octanol and in water and the degradation number of the *b*‐th conformer. *E*°^ct^ and *E* ^wat^ are the energy of the conformer in octanol and water, respectively.

The *D* value gives the density of molecules on the donor−‐membrane interface of the membrane. The summation of diffusions of all the conformers gives the total diffusion.

(11) $$LogP_{app}^{all}=Log\left(\frac{D}{h}\sum_{a=1}^{N_{conformer}}d_{mem}\left(a\right)M\left(a\right)\right)=LogD-Logh+Log<M\left(a\right){>}_{mem}$$

The fraction of the *a*‐th conformer in the membrane is given by

(12) $$d_{mem}\left(a\right)=\frac{n\left(a\right)\exp(-E^{mem}\left(a\right)/\left(k_{B}T\right)}{\sum_{b=1}^{N_{conformer}}n\left(b\right)\exp(-E^{mem}\left(b\right)/\left(k_{B}T\right)}$$

As in eq. 7, here we apply Kubo's cumulant expansion to eq. 11, giving

(13) $$LogP_{app}^{all}=LogD-Logh+<LogM\left(a\right){>}_{mem}-\frac{1}{2}<LogM{\left(a\right)}^{2}-{\left\langle LogM(a)\right\rangle }^{2}{>}_{mem}+\cdots$$

In permeability model B, the deviations of the descriptors relating to the diffusion contribute to Log*P*_app_^all^ besides the average values of the descriptors. Namely, the deviation of the radius of the solute should contribute to the prediction of Log*P*_app_.

### 2.3 Regression and Prediction

Our physical‐property prediction method was a principal component regression (PCR) with an L2 regularization term based on the molecular descriptors.^37^, ^38^ The regression model was the same as that used in our previous study. Namely, the principal component (PC) analysis projected each compound into each point in a chemical space of the PC, and a multiple linear regression was applied to the molecular coordinates in the chemical space. The principal component axes were selected to minimize the root‐mean‐square error (RMSE) in regression. The addition of the L2 term reduced the RMSE in the prediction.

The physical property of the *i*‐th molecule *V*(*i*) is estimated based on the molecular descriptors {*s*_i_^*b*^} where b represents the *b*‐th descriptor as follows.

(14) $$V\left(i\right)=\sum_{j=1}^{N_{axis}}c_{j}\cdot p_{i}^{j}+c_{{}_{0}}$$

(15) $$p_{i}^{j}=\sum_{b=1}^{N_{descriptor}}d_{b}^{j}\cdot \left(s_{i}^{b}-<s^{b}>\right)$$

where *c_j_*, *p*_i_^*j*^, and *d*_b_^*j*^ are the intercept parameter of the linear function, the PC vector, and the loading vector, respectively. The PC analysis of the descriptor *s_i_* ^*b*^ gives the loading vector *d*_b_^*j*^ and the PC vector (axis) *p*_i_^*j*^. The values of {*c_j_*} are determined by multiple linear regression (MLR). *N*_axis_ (*N*_axis_<*N*_descriptor_) is determined so as to maximize the Q^2^‐value obtained by cross validation tests. The parameters are determined based on the learning set and then are used for prediction. The objective function O({*c*}) consists of the summation of the square error between the experimental (*V* ^exptl^) and calculated (*V* ^calc^) property values with the following regularization term:

(16) $$O\left(\left\{c\right\}\right)=\;\sum_{i=1}^{N_{data}}{\left(V_{i}^{exptl}-V_{i}^{calc}\left(\left\{c\right\}\right)\right)}^{2}+\lambda \sum_{i=1}^{Naxis}{\left(c_{i}\right)}^{2}$$

Here, the parameter set {*c*} is determined to minimize the O value. The weighting parameter λ is fixed to a constant (λ=0.000001) in the present study.

We apply this regression model to *P*_app_, Log*P*, Log*D*, and p*K*_a_. Since the permeability depends on the Log*P*, Log*D*, and p*K*_a_, the *P*_app_ prediction model should include the descriptors for Log*D*, Log*P*, and p*K*_a_ prediction. All the prediction models shared the same molecular descriptors. We applied 4‐fold CV tests to evaluate the accuracy, and all the values in the tables were predicted in the 4‐fold CVs. The CV tests showed RMSE and Q^2^ values. The definitions of Q^2^ and RMSE are determined as follows.(17)
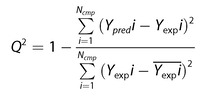

(18)
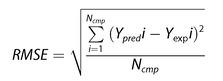



Here, *Y*_pred_ and *Y*_exp_ represent the predicted value in validation and the experimental *Y* value, respectively. In the present study, we do not compare the Q^2^ values of different properties, since the variances of the experimental data (denominator in eq. 17) differed from each other among the different data sets.^39^

### 2.4 Regression and Prediction

Models A and B use the degeneracy and energy of the conformers in eqs. 6 and 9 so that we must sample the conformers. Although an exhaustive search of conformers is very difficult and time‐consuming, many heuristic conformer generation methods have been proposed. The major approaches are random search and systematic search.^40^, ^41^, ^42^, ^43^, ^44^, ^45^, ^46^, ^47^ Especially, the conformer generation of ring systems is more difficult than that of nonring systems. For ring systems, some specialized methods, such as corner and edge flips, have been used.^40^, ^41^ We applied a Monte Carlo random‐sampling method.^42^ In conformer generation, a difficult problem is the estimation of degeneracy (*n*(*a*)) in eqs. 6 and 9. Most conformer generation programs focus on the reproduction of the most stable conformers precisely, but the degeneracy is unclear. Long‐time MD simulations could measure the degeneracy but are not realistic in this work, since there are too many molecules.

One of the simplest approaches should be a uniform sampling of conformers without solvent bias. If the conformer search was a random sampling of dihedral angles of rotatable bonds of a molecule, the generated structural ensemble should mimic the uniform sampling (see some discussions in APPENDIX A in the supporting information). Unfortunately, this approach could not work in the sampling of large or cyclic compounds, since the ring systems could be opened by the random rotations of the dihedral angles and an atomic collision could occur. Thus, we applied a force field to close the ring systems and to avoid atomic collisions. Our conformer generation program transforms the ring systems of a molecule to nonring systems by removing one of the bonds in the ring. The following energy optimization closes the rings. The force field used was an AMBER‐like one including 1–2, 1–3, 1–4, and 1–5 interactions. The 1–4 and 1–5 interaction terms consisted of only van der Waals interactions without electrostatic interactions, since the dielectric constant of water is very different from that of membrane.

A clustering analysis of the conformers generated above estimates the degeneracy number (*n*(*a*) in eqs. 6 and 9). The clustering threshold is the heavy atom RMSD<1.5 Å.

### 2.5 Descriptors

The molecular descriptors consisted of physical and chemical ones. The physical descriptors represent mainly the size of a molecule that is related to the diffusion. The accessible surface area (ASA) is a useful idea with which to represent the solute−solvent interaction. The chemical properties represent mainly the hydrophobicity/hydrophilicity of a molecule that is related to the distribution between water solvent and membrane. In general, charged molecules cannot penetrate a membrane whose dielectric constant is small, but neutral molecules can. The total charge of a molecule is determined by the p*K*_a_ and the MACCS key,^48^ which is a set of substructures that recognizes the functional groups closely related to p*K*_a_.^48^ The atomic charges were AM1‐BCC charges obtained by MOPAC7.^49^, ^50^, ^51^ The hydrogen bonds are determined for the hydrogen atoms of OH and NH groups and for the acceptor atoms with lone pairs (O, N, S). The GBSA method was used to calculate the Log*D* by eq. 10.^33^ Since the descriptors include the ASA, the ASA and the Log*D* term double‐counted the solute‐solvent interaction. The PCR could combine these dependent terms and thus reduce the number of independent fitting parameters. The atomic solvation parameters in the GBSA method were set to 10 cal/mol/Å2 and −5 cal/mol/Å2 for water and membrane, respectively.

Models A (eq. 9) and B (eq. 13) include both the average and deviation terms. The average <*A*> and deviation σ(A) of property *A* are determined as follows. The conformer generation program in section 2.4 generates the conformers.

(19) $$<A>=\frac{\sum_{i=1}^{N_{conformer}}n\left(i\right)A\left(i\right)\exp(-E^{sol}\left(i\right)/\left(k_{B}T\right))}{\sum_{i=1}^{N_{conformer}}n\left(i\right)\exp(-E^{sol}\left(i\right)/\left(k_{B}T\right))}$$

(20) $$\sigma \left(A\right)=\frac{\sum_{i=1}^{N_{conformer}}n\left(i\right){\left(A\left(i\right)-<A>\right)}^{2}exp\left(-E^{sol}\left(i\right)/\left(k_{B}T\right)\right)}{\sum_{i=1}^{N_{conformer}}n\left(i\right)exp(-E^{sol}\left(i\right)/\left(k_{B}T\right))}$$

where sol represents the solvent (water or membrane). *E* ^s^°^l^(*i*) is the relative energy of the *i*‐th conformer in the solvent compared to that in vacuum.

Since membrane permeability is related to Log*D*, or to Log*P* and p*K*_a_ values as described in eqs. 1, 3, 8, and 13, the descriptors that could approximate Log*D*, Log*P*, and p*K*_a_ values should contribute to *P*_app_ values. Dissociations of hydrogen atoms depend on the chemical bonds of the functional groups and the electrostatic field generated by the charge distribution of the solute. A previous work proposed a linear correlation between the proton chemical shift and the p*K*_a_ value, and the chemical shift depends on the electron density on the nucleus.^52^ Thus, we adopted the descriptors used in the Log*D* and p*K*_a_ predictions. Namely, we used the numbers of hydrogen donors and acceptors, the atomic charges of the hydrogen atoms of the NH and OH groups, and the MACCS key to represent the chemical structures of the solutes.

## 3 Data Preparation

The PAMPA permeability data and molecular structures were extracted from ChEMBL database version 24.^53^ The ChEMBL assay and compound IDs used are summarized in Table S1. Most of the experimental conditions included pH 7.4 and the observation times were several hours, but the details of the conditions varied. In addition, a variety of membrane materials have been used.^15^, ^26^, ^27^, ^28^, ^29^, ^30^, ^31^, ^32^ A desirable assay data set consists of the *P*_app_ values obtained under the unique experimental conditions.^18^ The careful data selection limited the number and diversity of examined compounds. While there have been differences in the *P*_app_ values observed through the PAMPA assay, the systems were designed to reproduce *P*_app_ values of the Caco‐2 assay system. To evaluate the wide variety of compounds, the present data set consisted of 737 compound data obtained under the heterogeneous experimental conditions.; There were 70 pairs of the same solute with different *P*_app_ values; for these 70 pairs, the average difference was 0.401 and the RMSD was 0.500. Thus, we discussed the prediction accuracy of Log*P*_app_ >0.5. As with the *P*_app_, we also prepared data on the octanol‐water distribution coefficient (Log*D*, 4215 compounds),^54^ and the dissociation coefficient (p*K*_a_, 240 compounds).^55^, ^56^

Astellas Pharmaceutical kindly provided additional experimental data, since there were fewer data on macrocyclic compounds than on acyclic compounds. The observed experimental property data were *P*_app_, Log*D*, and Log*S* data. The test compounds were 58 selected commercially available macrocyclic compounds. In addition, we asked Enamine Ltd. to evaluate the *P*_app_ values of two cyclic peptides using the PAMPA assay. The molecular structures and experimental values are summarized in Tables S1 and S2 in the supporting information.^57^

We prepared three‐dimensional molecular structures of the examined compounds in the present study. The computational procedure was summarized as APPENDIX B in the supporting information. The initial molecular structures were 2D planar structures without hydrogen atoms in the SD file format. A file transfer program, Hgene/myPresto, was used to prepare the three‐dimensional molecular structures with hydrogen atoms in electrically neutral forms including acids and amines. The atomic charges were the AM1‐BCC charges provided by the MOPAC program. A molecular dynamics simulation program, cosgene/myPresto, gave the energy‐optimized structures with the general AMBER force field (GAFF) assigned by the tplgeneL/myPresto topology generator.^58^, ^59^ The energy optimization gave one stable or meta‐stable molecular structure for each molecule in vacuum. All the molecular structures were prepared in the Sybyl mol2 file format.

The total data set consisted of 795 data points from the ChEMBL database and the unpublished assay data. Table 1 shows the distributions of molecular weight and the total number of atoms as well as the numbers of heavy atoms, ring structures, rotatable bonds, and atoms included in the biggest ring system of each molecule.

**Table 1:** Statistical features of the data set for permeability prediction.

	Average	Deviation
Molecular weight (Da)	424.1	160.4
No. of all atoms	54.5	23.1
No. of heavy atoms	29.6	11.4
No. of rings	3.2	1.3

## 4 Results and Discussion

### 4.1 Prediction Models for Models A and B

Our Log*P*_app_ prediction model represents the dissociation of solute in water, the distribution of solute from donor water (donor) to membrane, and the diffusion of solute in the membrane towards the water (acceptor) phase. Diffusion depends on the solute molecular radius *R*, and many previous works adopted *R* as the descriptor in Log*P*_app_ prediction.^2^, ^16^, ^23^, ^24^ The ES term (eq. 2) represents the viscous resistance, while the real solute molecule feels both the viscous resistance and the inertial resistance. The inertial resistance is proportional to the product of the cross section of the solute and the square of the velocity of the molecule. The Taylor series of the total diffusion becomes as follows.

(21) $$LogM=Log\left(\frac{1}{f_{v}R+f_{i}R^{2}}\right)=-Logf_{v}R\left(1+f_{i}/f_{v}R\right)=-Logf_{i}-LogR+\left(f_{i}/f_{v}\right)\cdot R\;-1/2{\left(f_{i}/f_{v}\right)}^{2}\cdot R^{2}+1/3{\left(f_{i}/f_{v}\right)}^{3}\cdot R^{3}+\cdots$$

where *R*, *f*_v_, and *f_i_* are the average radius of the solute and the respective coefficients of the diffusions related to the viscous and inertial resistance. In the actual calculation, we used the average *R* (denoted as <*R*>) instead of *R*. Thus, we described the diffusion term of Log*P*_app_ as a weighted summation of <*R*>, <*R*>^2^, <*R*>^3^, and Log<*R*>. Also, the <*R*>^3^ term represented the permeant's volume, since the transfer of the molecule from the donor phase to the membrane needed a volume change of the membrane, and this change generated additional energy corresponding to pressure in the membrane. In addition to these terms, we adopted the σ(*R*) term of the cumulant expansion (eqs. 7 and 13).

We discussed only a passive permeability. The Fick’ law (eq. 1) suggested that the permeability process consisted of several processes in a sequential order (the diffusions in water, the partitioning between water and membrane, the diffusion in membrane) and the Log*P*_app_ could be given by a summation of the effects of these processes. In addition, our modifications (eqs. 7, 13 and 21) could be represented by linear regression models. Thus, we started from simple linear regression models as the QSPR equations based on Models A (eq. 22) and B (eq. (22).

(22) $$LogP_{app}^{ModelA}=c^{D}<LogD{\left(a\right)}_{ow}^{calc}{>}_{wat}+c^{E}\sigma {\left(LogD{\left(a\right)}_{ow}^{calc}\right)}_{wat}+\sum_{i=1}^{NA}c_{i}^{AW}\cdot <A_{i}{>}_{wat}+\sum_{i=1}^{NA}c_{i}^{AM}\cdot <A_{i}{>}_{mem}+c^{B}\cdot <B{>}_{wat}+c^{r}\cdot <R{>}_{wat}+c^{S}\cdot <R{>}_{wat}^{2}+c^{V}\cdot <R{>}_{wat}^{3}+c^{r}\cdot \sigma {\left(R\right)}_{wat}+c^{l}\cdot log<R{>}_{wat}+c^{OH}\cdot q\left(OH\right)+c^{NH}\cdot q\left(NH\right)+c^{atom}\cdot N_{atom}+c^{rot}\cdot N_{rot}++c^{HA}\cdot N_{HA}+c^{HD}\cdot N_{HD}+\sum c_{i}^{MACCS}\cdot MACCS_{i}+c^{0}$$

and

(23) $$LogP_{app}^{ModelB}=c^{D}\cdot Log_{10}D_{ow}^{calc}+\sum_{i=1}^{NA}c_{i}^{AW}\cdot <A_{i}{>}_{wat}+\sum_{i=1}^{NA}c_{i}^{AM}\cdot <A_{i}{>}_{mem}+c^{B}\cdot <B{>}_{mem}+c^{r}\cdot <R{>}_{mem}+c^{S}\cdot <R{>}_{mem}^{2}+c^{V}\cdot <R{>}_{mem}^{3}+c^{r}\cdot \sigma {\left(R\right)}_{mem}+c^{l}\cdot log<R{>}_{mem}+c^{OH}\cdot q\left(OH\right)+c^{OH}\cdot q\left(OH\right)+c^{atom}\cdot N_{atom}+c^{rot}\cdot N_{rot}+c^{HA}\cdot N_{HA}+c^{HD}\cdot N_{HD}+\sum c_{i}^{MACCS}\cdot MACCS_{i}+c^{0}$$

where *A_i_*, *B*, *q*(OH), *q*(NH), *N*_atom_, *N*_rot_, *N*_HA_, *N*_HD_, and MACCS represent the ASA of the *i*‐th atom type, the number of intramolecular hydrogen bonds, the maximum atomic charge of the H atom in the OH groups, that in the NH groups, the number of atoms, the number of rotational bonds, that of hydrogen donors, that of acceptors, and the MACCS key. The coefficient *c*’s (*c* ^D^, *c* ^AW^, *c* ^AM^, *c* ^B^, *c* ^R^, *c* ^S^, *c* ^V^, *c* ^r^, *c* ^l^, *c*°^H^, *c* ^NH^, *c* ^at^°^m^, *c* ^r^°^t^, *c* ^HA^, *c* ^HD^, *c* ^MACCS^, and *c* ^0^) are the fitting parameters determined by the regression. The atom type was Sybyl mol2.

### 4.2 Prediction Results by Models A and B: Conformer Dependence

To examine the adequacy of Models A and B, we calculated the conformer dependence of each. Before the validation of the prediction models, we examined the conformer generation. Table 2 shows the average values of <*R*>_wat_ and <*R*>_mem_, σ(*A*)_wat_, and σ(*A*)_mem_ over all of the 795 solute molecules in the dataset, when the number of conformers was set to 100. The conformer generation represented the solute‐size change in water and in membrane. The results supported previous findings.^18^, ^19^, ^20^, ^21^, ^22^, ^25^ Namely, the solutes in membrane were folded into smaller compact structures than those in water (<*R*>_mem_<<*R*>_wat_), and the solutes in water fluctuated more than those in membrane (σ(*R*)_mem_<σ(*R*)_water_). These results showed a consensus with the previously reported phenomena.^18^, ^19^, ^20^, ^21^, ^22^, ^25^ Thus, we applied this conformer generation method in the present study.

**Table 2:** Statistics on diffusion‐related values of 795 compounds at *N*_structure_=100.

Term	Average	Deviation	Min	Max
<*R*>_wat_^a^	4.27	0.80	1.73	7.07
<*R*>_mem_^a^	3.85	0.75	1.73	6.44
σ(*R*)_wat_^a^	0.28	0.17	0.00	0.64
σ(*R*)_mem_^a^	0.03	0.05	0.00	0.51
<*B*>_wat_^b^	0.02	0.13	0.00	1.41
<*B*>_mem_^b^	0.02	0.16	0.00	2.00

^a^: units in Å. ^b^: number of intramolecular hydrogen bonds.

**Figure 2:**
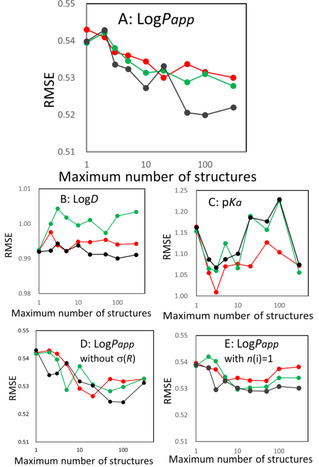
Conformer‐number dependence of predicted properties. X axis is in Log scale. Red, green, and black circles represent the RMSE values obtained by Models A, B, and AB, respectively. (A) Log*P_app_*, (B) Log*D*, (C) p*K_a_*, (D) Log*P_app_* without σ(R) terms, and (E) Log*P_app_* values with *n*(*i*)=1, respectively.

We applied Models A and B to the ensemble of conformers by restricting the maximum number of generated conformers up to 300. Then the 4‐fold CVs of Log*P*_app_ predictions were used to estimate the Q^2^ and RMSE values. Figure 2 and Table S3 show the conformer‐number dependence of the Log*P*_app_ prediction. Both Models A and B worked well, and the increase in conformers improved the prediction accuracy. Some previous works showed that the conformer change increased the membrane permeability.^3^, ^6^, ^7^, ^24^, ^25^ Namely, if the molecule has some conformers that show hydrophilic or hydrophobic surfaces, it can select stable conformers depending on the solvent (hydrophobic conformer in lipid and hydrophilic conformer in water).

There are two problems that make the validation of the models difficult. One of the problems is the lack of sufficient experimental observations of the conformational dynamics of solute in permeability process. Solution NMR experiments could determine the conformations in different environments including those mimic membrane interiors. However, available NMR experimental data that directly compare aqueous and lipid bilayer environments are rather limited. Often, the limited solubilities of middle molecules prevent the observation of NMR signals in aqueous solution.

In addition, most of the permeability data are for small molecules, which are less flexible than middle molecules and have smaller number of possible conformers. There are only small number of experimentally determined permeability data on middle molecules to establish the dynamics‐activity relationships. The limited number of flexible molecules with membrane permeability data also causes the problem in validating of the models based on the prediction accuracy. Recent progress of the solution NMR and molecular dynamics simulations of membrane systems should elucidate and validate the permeability mechanism in near future.

Table S4 also shows the average and maximum CPU times elapsed for one molecule. The longest CPU time was less than 62 minutes. The calculation speed should be faster than the precise MD simulation.

The results obtained by Model A were close to those by Model B. Thus, we combined the models into one by merging their descriptors. Model AB represents the united model, and the fitting parameters were determined in the same way as for Models A and B. The results obtained by Model AB were slightly better than those by Models A and B (see Figure 2 and Table S3). The RMSE values were about 0.5 and close to the deviation of the experimental data (about 0.4). Since these results showed that both Models A and B were possible permeability processes, we could not clarify the ratio of the speeds of conformer change and diffusion. The ratio should be different for each molecule in the data set.

The conformer generation represented two effects. One was the molecular‐size change depending on the solvent. The radius of the molecule in water was different from that in membrane. One conformer rich of intra‐molecule hydrophobic contacts could be dominant in water and the other conformer rich of intra‐molecule hydrogen bonds could be dominant in water. The other effect was the structural fluctuation of the molecule. The dynamics of the molecule in water were different from those in membrane. In this case, multiple conformers existed in water and in membrane, and the population of conformers in water could be different from that in membrane. The deviation terms (σ(*R*)) in eqs. 22 and 23 correspond to this structural fluctuation of the solute molecule. To evaluate the effect of this deviation term, we removed the deviation terms from eqs. 22 and 23. Figure 2D shows the RMSE values obtained by Models A, B, and AB without the σ(*R*) terms. The σ(*R*) terms did not improve the accuracy drastically as the number of conformers increased. These results suggested that the structural change of the solute molecule was important in the Log*P*_app_ prediction, but the dynamic structural fluctuation of the solute molecule in each condition was not so important in the Log*P*_app_ prediction.

We examined which terms in eqs. 22 and 23 reflect the conformer number dependence. The permeability process includes the dissociation of solutes in water and the distribution of electrically neutral solute molecules from water to membrane. These processes relate to the Log*D*, Log*P*, and p*K*_a_ values. Thus, the permeability‐prediction models (eqs. 22 and 23) should predict the Log*D*, Log*P*, and p*K*_a_ values by adjusting the coefficients {***c***} of eqs. 22 and 23. Figures 2B and 2 C show the conformer dependences of the predicted Log*D* and p*K*_a_ values, respectively. The prediction models worked and the RMSE values of these predictions were similar to those previously reported. These predicted values did not show clear dependence on the number of conformers. Using the same model, only the predicted Log*P*_app_ values showed the conformer‐number dependence among these properties. Considering eq. 1, these results suggested that the conformer‐number dependence of Log*P*_app_ originated from the diffusion process (*M* in eq. 1) in the present models and that the <*R*> terms in eqs. 22 and 23 should contribute to the conformer‐number dependence of Log*P*_app_.

We also examined the RMSE and Q^2^ values when all the degeneracies of conformers were identical (*n*(*i*)=1 in eqs. 19 and 20). Figure 2E shows the conformer dependence of the predicted Log*P*_app_ values with *n*(*i*)=1. The results were worse than those with estimated degeneracy, and increasing the number of generated conformers did not improve the RMSE. These results suggested that the conformer generation worked properly and that the estimation of conformer populations was important in the Log*P*_app_ prediction.

### 4.3 Contribution of Diffusion Terms to Log*P_app_* Prediction

We examined the diffusion process in terms of viscous (ES term, eq. 2) and inertial resistances. Table 3 shows the Q^2^ and RMSE values for various combinations of diffusion terms. The diffusion was described by the Log<*R*>, <*R*>, <*R*>^2^, <*R*>^3^, and σ(*R*) terms in eqs. 22 and 23. Since the Taylor series in eq. 21 includes higher‐order terms than <*R*>^3^, we examined the effect of <*R*>^4^ too. <*R*>^3^ was proportional to the volume of solute molecule, but it did not originally refer to volume. <*R*>^2^ and <*R*>^3^ correspond to the cross section of the solute molecule and the cross section of it that causes inertial resistance. The <*R*> and Log<*R*> terms represented viscous resistance. The <*R*>^2^ and <*R*>^3^ terms improved the accuracy, as did the ES term that corresponded to <*R*> and Log<*R*>. The higher‐order term (<*R*>^4^) and the deviation σ(*R*) did not improve the results so much.

**Table 3:** Log*P_app_* prediction results obtained by various diffusion terms at N_structure_=100.

Diffusion term including R	Model A		Model B		Model AB	
	RMSE	Q^2^	RMSE	Q^2^	RMSE	Q^2^
None	0.54	0.77	0.54	0.77	0.54	0.77
<*R*>	0.54	0.77	0.54	0.78	0.54	0.77
Log<*R*>	0.54	0.78	0.53	0.78	0.54	0.78
σ(*R*)	0.54	0.77	0.54	0.77	0.54	0.77
*R*, σ(*R*)	0.54	0.77	0.53	0.78	0.54	0.78
Log<*R*>, σ(*R*)	0.53	0.78	0.53	0.78	0.53	0.79
<*R*>, Log<*R*>	0.53	0.78	0.53	0.78	0.53	0.78
<*R*>, Log<*R*>, σ(R)	0.53	0.78	0.53	0.78	0.53	0.79
{<*R*>^n^; n=1–2}, Log<*R*>, σ(*R*)	0.53	0.78	0.53	0.78	0.53	0.79
{<*R*>^n^; n=1–3}, Log<*R*>, σ(*R*)	0.53	0.78	0.52	0.79	0.52	0.79
{<*R*>^n^; n=1–2}, Log<*R*>	0.53	0.78	0.54	0.78	0.53	0.78
{<*R*>^n^; n=1–3}, Log<*R*>	0.53	0.78	0.53	0.78	0.52	0.79

### 4.4 Molecular Weight and Ring‐Size Dependences

**Figure 3:**
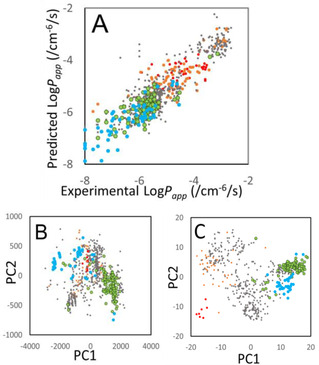
Predicted and experimental Log*P*_app_ (A), chemical spaces (B) based on the descriptors in eq. 23 and (C) based on the Mordred descriptors in term of *MW*, respectively. The model used is Model B at *N*_struct_=100. Red, orange, gray, green, and blue spheres represent the molecules with 0<*MW*<150 Da, 150 Da<*MW*<300 Da, 300 Da<*MW*<500 Da, 500 Da<*MW*<600 Da, and 600 Da<*MW*, respectively.

**Figure 4:**
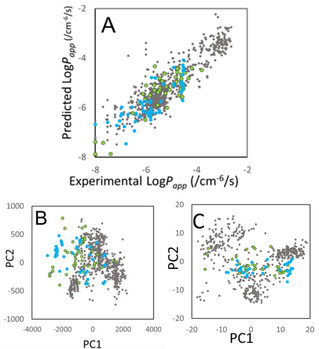
Predicted and experimental Log*P*_app_ (A) and chemical spaces (B) based on the descriptors in eq. 23 and (C) based on the Mordred descriptors, respectively. The model used is Model B at *N*_struct_=100. Gray, green, and blue spheres represent the molecules with *N_ring_* C<12, 12<*N_ring_*<20, and 20<*N_ring_*, respectively.

We examined how Models A and B worked in the prediction of the Log*P*_app_ of middle molecules. If the prediction model was adequately constructed, the predicted results should not depend on the molecular size. Since molecules with *MW*>500 Da and those with *N*_ring_ (the number of ring‐member atoms of the biggest ring) >12 are so‐called middle molecules and macrocyclic molecules, respectively, we examined the *MW* and *N*_ring_ dependences of the predicted data distributions. Figures 3 and 4 show the prediction results obtained by the 4‐fold CV of Model B at *N*_structure_=100. Figure 3A and 4 A show the correlations between the predicted and experimental Log*P*_app_ values, and Figure 3B and 4B show the principal component analysis results as the chemical space of the data points. Since Models A and AB showed the same trends as Model B, these results were omitted.

The colors in Figure 3 represent molecular weight (*MW*). The overall trend was that the set of small molecules showed high permeability and that of large molecules showed low permeability, but the overlaps among different color points were spread across a wide area. The overlap among the smaller and bigger molecules showed wide distribution in the chemical space. The RMSE did not depend on the *MW* so much. These results suggested that the present prediction model should be robust against the *MW* difference.

The colors in Figure 4 represent the number of ring‐member atoms of the biggest ring (*N*_ring_). The distributions of the same color data points were not localized in the Log*P*_app_ prediction and chemical space, or the data points of similar size compounds spread across a wide area. The distributions of middle molecules and macrocycles overlapped that of small molecules. The RMSE did not depend on the *N*_ring_ so much. In addition, Table 4 shows the dependence of the RMSE on the number of rotational bonds (*N*_rot_). *N*_rot_ represents the flexibility of a molecule. As with the *MW* and *N*_ring_, the *N*_rot_ dependence of Log*P*_app_ was weak. These results suggested that the prediction model worked well in the wide molecular‐size range and should be robust against various molecular sizes and shapes.

**Table 4:** RMSE and Q^2^ values in terms of molecular size at *N*_structure_=100.

Molecular features	No. of mols	Model A		Model B^b^		Model AB	
		RMSE	Q^2^	RMSE	Q^2^	RMSE	Q^2^
*MW*<150	37	0.47	0.36	0.45	0.43	0.46	0.40
150<*MW*<500	571	0.52	0.78	0.50	0.80	0.49	0.80
500<*MW*	187	0.40	0.77	0.39	0.78	0.38	0.78
*N_rot_*<10	242	0.48	0.73	0.45	0.76	0.45	0.77
10<*N_rot_*<20	448	0.50	0.81	0.49	0.82	0.48	0.82
20<*N_rot_*	99	0.46	0.84	0.43	0.86	0.44	0.85
*N_ring_*<6	511	0.52	0.81	0.50	0.82	0.50	0.82
6<*N_ring_*<11	171	0.41	0.71	0.39	0.73	0.37	0.76
11<*N_ring_*	111	0.47	0.80	0.42	0.84	0.44	0.82

We depicted the PCA plots based on Mordred descriptors to examine the chemical space of the collected molecules in the present study.^61^ Mordred consists of 1826 molecular descriptors and it has been widely used in the chemoinformatics. Figures 3C and 4 C show the results. As same as Figures 3B and 3 C, the distributions of molecules were widely spread, and the groups of molecules colored according to their size and shape distributed contiguously except very small molecules <150 Da (colored in red). Thus, most of the data should be suitable for the present analysis..

### 4.5 Verifications of Models A and B

To verify Models A, B and AB, we also examined the L1 regularization (eq. 24) instead of the L2 regularization in eq. 16.

The L1 regularization could show the descriptors that show the major contributions to the results. The accuracy was almost equivalent to that obtained by the L2 regularization. Namely, Models A, B, and AB showed the RMSE and Q^2^ values of 0.73 and 0.60, respectively, in the 4‐fold cross validation tests (Table 5). The important descriptors suggested by the L1 regularization were the <*R*>, Log<*R*>, <*R*>^2^, some ASA, mainly sub‐structures including O and N atoms, and *q(OH)*/*q(NH)* among 324 descriptors including 166 MACCS keys. The results were summarized in Table S5 and Figure S1 in the supporting information. The results supported the results obtained by the present regression models.

**Table 5:** RMSE and Q^2^ values obtained by eq. 24 (L1 regularization).

	Model AB	Model A	Model B
RMSE	0.59	0.59	0.59
Q^2^	0.72	0.73	0.73

We examined the robustness of the present method by using hold‐out tests and compared the results obtained by the Mordred‐descriptor set as an alternative method. We examined five hold‐out tests and compared the coefficient sets {***c***} of generated regression models, in addition to the comparisons of predicted Log*P*_app_ values. In each hold‐out test, the molecules were sorted by the molecular features and the top 25 % bigger molecules form the hold‐out set and the other smaller 75 % of molecules were used for the prediction model construction. The considered molecular features were the molecular weight (*MW*), number of atoms (*N_atom_*), number of ring structures (*N_cycle_*), number of rotational bonds (*N_rot_*), and the number of member atoms of the maximum ring system of molecule (*N_ring_*). In addition to these biased sets, we prepared a non‐biased set (None) by a random selection of molecules to make a reference prediction model.

In each hold‐out test, we applied the 3‐fold cross validations to the teaching sets (the smaller molecules) and generated the prediction models. The prediction models estimated the Log*P*_app_ values of the molecules in the hold‐out set (the bigger molecules). Table 6 summarizes the correlation coefficients (*R*) and the RMSE between the predicted and experimental Log*P*_app_ values of the hold‐out set. These results show that both the present and Mordred descriptors worked well (see Figure S2 in the supporting information).

**Table 6:** RMSE and *R* values obtained by the hold‐out tests.

Molecular feature	The present descriptors (eq. 23)			
	L1 (eq. 24)^a^		L2 (eq. 16)^a^	
	R	RMSE	R	RMSE
None^b^	0.84	0.64	0.89	0.54
*MW*	0.43	0.73	0.78	0.54
*N_atom_*	0.81	0.78	0.90	0.58
*N_cycle_*	0.45	0.62	0.59	0.53
*N_rot_*	0.82	0.80	0.89	0.63
*N_ring_*	0.68	0.71	0.84	0.51

Molecular feature	Mordred descriptors			
	L1		L2	
	R	RMSE	R	RMSE
None^b^	0.81	0.68	0.89	0.53
*MW*	0.58	0.72	0.83	0.47
*N_atom_*	0.84	0.74	0.91	0.55
*N_cycle_*	0.48	0.59	0.66	0.49
*N_rot_*	0.84	0.76	0.90	0.61
*N_ring_*	0.71	0.68	0.81	0.55

^a^: Model B was used. ^b^: The reference models used in Table 7.

Then, we examined the robustness of the model construction. Table 7 shows the correlation coefficients between the coefficients {***c***} of the prediction model obtained by the hold‐out test and that of the reference model. The {***c***} based on the present descriptors did not depend on the difference of the teaching sets so much. On the other hand, the {***c***} based on the Mordred descriptors depended on the difference of the teaching sets strongly. The PAMPA systems represent a passive permeability only. It means that the {***c***} does not depend on the choice of the teaching sets. Thus, the present method should be realistic and useful rather than the collection of many descriptors.

**Table 7:** Correlation coefficients (*R*) among {***c***} of the defferent prediction models in the hold‐out tests.

Molecular feature	The present descriptors (eq. 23)		Mordred descriptors	
	L1 (eq. 24)	L2 (eq. 23)	L1	L2
*NW*	1.00	0.94	0.70	0.90
*N_atom_*	1.00	0.95	0.71	0.84
*N_cycle_*	1.00	0.62	0.33	0.60
*N_rot_*	1.00	0.90	0.75	0.86
*N_ring_*	1.00	0.88	0.81	0.63

## 5 Conclusion

We proposed a QSPR method for for evaluating the apparent membrane permeability (*P*_app_) based on an analysis of the diffusion process and the partition function calculation with conformer sampling. This method generated conformers of the solute by a random structural sampling and the following structure optimization. The molecular descriptors were calculated based on the structural ensemble of the solute. Namely, the descriptors were the average and deviation values of the calculated ASA, the Log*D* and number of hydrogen bonds in water and in membrane, and the MACCS key. In addition, the descriptor set included a diffusion coefficient that is the inverse of the resistance in diffusion. To estimate the diffusion of solute, we examined the inertial resistance in the diffusion of solute in addition to the viscous resistance. We assumed two types of permeability models of solute with multiple conformers. One was the fast diffusion process (Model A), in which the solute diffused in the membrane with a fixed conformer, whose fractions were the same as those in water. The other was the slow diffusion process (Model B), in which the solute changed the conformer in the diffusion process and the fractions of conformers followed the most stable distribution in the membrane. The present QSPR models represented Models A and B based on the molecular descriptors mentioned above.

This prediction method worked in the *P*_app_ prediction of the middle molecules and macrocycles, the same as with that of the small molecules. The results suggested that the inertial resistance should be important in the diffusion, as is the viscous resistance known as the Einstein‐Stokes equation. The ensemble of conformers improved the prediction accuracy. This study supported both Models A and B, and the permeability process could be a combination of Models A and B.

## Conflict of Interest

None declared.

## Supporting Information

The appendices, Tables S1–S5, Figures S1 and S2 were supplied as described in the Supporting Information.

## Supporting information

As a service to our authors and readers, this journal provides supporting information supplied by the authors. Such materials are peer reviewed and may be re‐organized for online delivery, but are not copy‐edited or typeset. Technical support issues arising from supporting information (other than missing files) should be addressed to the authors.

Supplementary
