# Supplementary material for: Prediction of Passive Membrane Permeability by Semi‐Empirical Method Considering Viscous and Inertial Resistances and Different Rates of Conformational Change and Diffusion
Source: Mol Inform. 2019 Oct 14;39(1-2):1900071. doi: 10.1002/minf.201900071 (PMC7050510; doi:10.1002/minf.201900071)
Supplement: Supplementary file 1 — Supplementary [file MINF-39-1900071-s001.pdf]

molecular informatics

Supporting Information

© Copyright Wiley-VCH Verlag GmbH & Co. KGaA, 69451 Weinheim, 2020

Prediction of Passive Membrane Permeability by Semi-Empirical Method Considering Viscous and Inertial Resistances and Different Rates of Conformational Change and Diffusion

Yoshifumi Fukunishi,* Tadaaki Mashimo, Takashi Kurosawa, Yoshinori Wakabayashi, Hironori K. Nakamura, and Koh Takeuchi© 2019 The Authors. Published by Wiley-VCH Verlag GmbH & Co. KGaA. This is an open access article under the terms of the Creative Commons Attribution License, which permits use, distribution and reproduction in any medium, provided the original work is properly cited.

Prediction of passive membrane permeability by semi-empirical method considering viscous and inertial resistances and different rates of conformational change and diffusion

Yoshifumi Fukunishi *[a], Tadaaki Mashimo [b, c], Takashi Kurosawa [b, d], Yoshinori Wakabayashi [e], Hironori K. Nakamura [f], Koh Takeuchi [a]

[a] Molecular Profiling Research Center for Drug Discovery (molprof), National Institute of Advanced Industrial Science and Technology (AIST), 2-3-26, Aomi, Koto-ku, Tokyo 135-0064, Japan

[b] Technology Research Association for Next-Generation Natural Products Chemistry, 2-3-26, Aomi, Koto-ku, Tokyo 135-0064, Japan

[c] IMSBIO Co., Ltd., Owl Tower, 4-21-1, Higashi-Ikebukuro, Toshima-ku, Tokyo 170-0013, Japan

[d] Hitachi Solutions East Japan, 12-1 Ekimaehoncho, Kawasaki-ku, Kawasaki, Kanagawa 210-0007, Japan

[e] BY-HEX LLP, 1-19-14, Shimizu, Suginami-ku, Tokyo 167-0033, Japan

[f] Biomodeling Research Co., Ltd., 1-704-2 Uedanishi, Tenpaku-ku, Nagoya, Aichi 468-0058, Japan

APPENDIX A: Estimation of fractions of conformers

The following procedure generated the molecular structures used in the present study.

In equations 4 and 11, the fraction of the a-th conformer in a solvent (=*d*(*a*)) is given by equations 6 and 12 as follows.

$$d(a) = \frac{n(a)\exp(-E(a)/(k_B T)}{\sum_{b=1}^{N_{conformer}} n(b)\exp(-E(b)/(k_B T)}$$ eq. S1

In the classical force field, E is the energy of the molecule in a solvent that is the summation of the intra-molecular chemical bond, angle, torsion, van der Waals, electrostatic potential energies of all atom-pairs of the molecule, and solvation energy in the solvent. The partial atomic charges are fixed to constant values. The solvation affects only the electrostatic potential energy of the molecule and the solvation energy. This energy decomposition shows that E is the summation of the solvent-independent energy and the solvent-dependent energy.

Let the number of atoms of the molecule is N and {x_1, x_2, ..., x_{3N}} is the coordinates in the Cartesian coordinates. "$x_{3*(m-1)+1}$", " $x_{3*(m-1)+2}$", "$x_{3*(m-1)+3}$" represent the x, y, z coordinates of the m-th atom of the molecule, respectively. Define the a-th conformer as the set of coordinates {x_1, x_2, ..., x_{3N}} in a region Va, the statistical dynamics shows that d(a) is given by the following integral eq. S2.

$$d(a) = \frac{\int_{Va}\cdots\int_{Va} \exp\left(-\frac{E(x_1, x_2, \cdots, x_{3N})}{k_B T}\right) dx_1 dx_2 \cdots dx_{3N}}{\int\cdots\int \exp\left(-\frac{E(x_1, x_2, \cdots, x_{3N})}{k_B T}\right) dx_1 dx_2 \cdots dx_{3N}}$$ eq. S2

The random sampling of the set of coordinates {x_1, x_2, ..., x_{3N}} could approximate eq. S2 as follows.

$$d(a) \approx \frac{\sum_{\{x_1, x_2, \cdots x_{3N}\} \in a-th\ conformer} \exp\left(-\frac{E(x_1, x_2, \cdots, x_{3N})}{k_B T}\right)}{\sum_{\{x_1, x_2, \cdots x_{3N}\} \in all\ possible\ coordinates} \exp\left(-\frac{E(x_1, x_2, \cdots, x_{3N})}{k_B T}\right)}$$ eq. S3

Only when the set of coordinates {x_1, x_2, ..., x_{3N}} satisfies the regular bond lengths, angles, torsions without inter-atomic collisions, the term

$\exp\left(-\frac{E(x_1, x_2, \cdots, x_{3N})}{k_B T}\right)$ could be > 0, otherwise, $\exp\left(-\frac{E(x_1, x_2, \cdots, x_{3N})}{k_B T}\right) \approx 0$, since the violations of these constraints cause high E values. Thus, we can calculate eq. S3 using the randomly selected {x_1, x_2, ..., x_{3N}} that satisfy the possible molecular structures of the given molecule.

Since the bond/angle energies and the atomic collision energy (van der Waals energy term) are solvent-independent, the molecular structures with fixed bond lenths and agles could be acceptable in any solvent. In the present study, we approximated the sets of $\{x_1, x_2, ..., x_{3N}\}$ obtained from the randomly rotated conformers with fixing the bond lengths and the bond angles without atomic collisions. Then, we calculated the solvent-dependent energies of based on the $\{x_1, x_2, ..., x_{3N}\}$ the generalized-Born solvent-acceptable surface area method.

APPENDIX B: Preparation of 3-dimensional molecular structures

The following procedure generated the molecular structures used in the present study.

Step 1. Generation of rough 3-dimensional (3D) molecular structures from the initial 2D molecular structures
The molecular structure files in 2D SDF format were extracted from the ChEMBL home page. Hgene/myPresto software generated the electrically neutral structure by addition of hydrogen atoms to the molecule. At the same time, Hgene roughly generated the 3D structure with the Gasteiger atomic charges as follows.

```
Hgene -imdl input.sdf -3D -h -omol2 temp.mol2
```

Step 2. Assignment of the force field parameters to the molecule
Energy minimization calculations require force filed parameters. TplgeneL/myPresto software assigned the GAFF force field to the molecule as follows.

```
tplgeneL -i temp.mol2 -ft 2 -r LIG -f no -p 3 -d gaff21.db
```

This process generates two output files. One is “temp.tpl” as a molecular topology file including the force field parameters and the other is “temp_tplL.pdb” as an initial coordinates file in the PDB format.

Step 3. Energy minimization
Cosgene/myPresto software performed energy minimization using these two files. The following example is a c-shell type command on Linux for energy minimization of 2000 steps of steepest descent method with distance-dependent dielectric constant.

```
cosgene << EOF
EXE> INPUT
  COORDI = PDB
  NAMECO = temp_tplL.pdb
  TOPOLO = FORM
  NAMETO = temp.tpl
  QUIT
EXE> MIN
  LOOPLI=2000
  CUTLEN=100.0
  DIFUN= DIST   DIEVAL = 8.0
  UPDATE=10
```

```
   QUIT
EXE> OUTPUT
   COORDI = PDB
   NAMECO = minimized.pdb
EOF
```

The energy minimized coordinates are stored in the PDB format. Tpl2mol2/myPresto software transforms the energy-minimized structure in the PDB file format into the MOL2 format.

```
tpl2mol2 -ipdb minimized.pdb -itpl lig_tplL.pdb -omol2 minimized.mol2
```

Step 4. Atomic partial charges by semi-empirical quantum-chemistry calculation
Hgene/myPresto software performed the AM1-BCC atomic charges by MOPAC7. This step gives the final 3D molecular structure files in Sybyl mol2 format. The AM1-BCC calculation can give the almost equivalent results to that obtained by the RESP RHF/6-31G* ab-initio quantum chemistry calculation.

```
Hgene -imol2 minimized.mol2 -mop AM1BCC -omol2 final_structure.mol2
```

After these steps, Discgene/myPresto software generated the ensemble of conformers and calculates the descriptors of the molecule. Finally, we can apply the regression and prediction calculations.

Table S1 Compound IDs, the structures in SMILES format and the experimental LogP_{app} values

No.	Compound ID	SMILES	Assay ID	LogP_{app}
1	CHEMBL121893	[N@H+]2(CCCCC2)CCC(=O)c1ccc(OCCCCCC)cc1	CHEMBL1034536	-2.67
2	CHEMBL538150	C(=O)(c1cc2c(cc(OCCCCCC)cc2)cc1)CC[N@H+](C)C	CHEMBL1034536	-2.85
3	CHEMBL539139	C(=O)(c1ccc(OCCOCC)cc1)CC[N@@H+](C)C	CHEMBL1034536	-3.80
4	CHEMBL539393	C(=O)(c1cc(C)c(OCCCCCC)cc1)CC[N@H+](C)C	CHEMBL1034536	-2.91
5	CHEMBL539718	C(=O)(c1c(Cl)cc(SCCCCCC)cc1)CC[N@@H+](C)C	CHEMBL1034536	-3.22
6	CHEMBL540227	C(=O)(c1ccc(OCCCCCC)cc1)CCNc2cc(Cl)ccc2	CHEMBL1034536	-5.70
7	CHEMBL540471	C(=O)(c2c(Cl)cc(OCCCCCC)c(Cl)c2)CC[N@@H+]1CCOCC1	CHEMBL1034536	-2.75
8	CHEMBL541478	N1C(=O)C[N@H+](CCC(c2c(C)cc(S(=O)(CCCCCC)=O)cc2C)=O)CC1	CHEMBL1034536	-3.32
9	CHEMBL541481	C(=O)(c2c(Cl)cc(SCCCCCC)cc2)CC[N@@H+]1CCOCC1	CHEMBL1034536	-2.69
10	CHEMBL549482	C(=O)(c1cc(C)c(OCCCCCC)c(C)c1)CC[N@H+](C)C	CHEMBL1034536	-3.14
11	CHEMBL549483	C(=O)(c1c(C)cc(OCCCCCC)cc1C)CC[N@H+](C)C	CHEMBL1034536	-3.19
12	CHEMBL549557	C(=O)(c1cc(S(=O)(C)=O)c(OCCCCCC)cc1)CC[N@H+](C)C	CHEMBL1034536	-3.14
13	CHEMBL549561	C(=O)(c1c(C)cc(S(=O)(CCCCCC)=O)cc1C)CC[N@@H+]2CCN(S(=O)(CC)=O)CC2	CHEMBL1034536	-3.00
14	CHEMBL549562	C(=O)(c1c(Cl)c(Cl)c(S(=O)(CCCCCC)=O)cc1)CC[N@H+](C)C	CHEMBL1034536	-3.66
15	CHEMBL549685	C(=O)(c1cc(Cc2ccccc2)c(OCCCCCC)cc1)CC[N@@H+](C)C	CHEMBL1034536	-3.64
16	CHEMBL549889	C(=O)(c1c(Cl)cc(S(=O)(CCCCCC)=O)cc1Cl)CC[N@H+]2CCN(C(=O)C)CC2	CHEMBL1034536	-3.32
17	CHEMBL550090	C(=O)(c2c(C)cc(S(=O)(CCCCCC)=O)cc2C)CC[N@@H+]1CCOCC1	CHEMBL1034536	-2.94
18	CHEMBL550094	C2[C@](C[N@H+](C)C)C(=O)c1ccc(OCCCCCC)cc1O2	CHEMBL1034536	-3.16
19	CHEMBL550295	N1C(=O)C[N@H+](CCC(c2c(Cl)c(Cl)c(S(=O)(CCCCCC)=O)cc2)=O)CC1	CHEMBL1034536	-2.96
20	CHEMBL550497	C(=O)(c2c(Cl)c(Cl)c(OCCCCCC)cc2)CC[N@@H+]1CCOCC1	CHEMBL1034536	-3.03
21	CHEMBL550752	C(=O)(c1c(Cl)cc(SCCCCCC)cc1Cl)CC[N@@H+]2CCN(C(C)=O)CC2	CHEMBL1034536	-2.98
22	CHEMBL550758	C(=O)(c1c(Cl)cc(OCCCCCC)cc1Cl)CC[N@H+](C)C	CHEMBL1034536	-3.16
23	CHEMBL550760	C(=O)(c1c(C)cc(SCCCCCC)cc1C)CC[N@@H+]2CCN(S(=O)(CC)=O)CC2	CHEMBL1034536	-3.80
24	CHEMBL550761	C(=O)(c1c(Cl)cc(S(=O)(CCCCCC)=O)cc1)CC[N@@H+](C)C	CHEMBL1034536	-3.42
25	CHEMBL550765	C(=O)(c1ccc(OCCCCCC)cc1)[C@@](C[N@@H+](C)C)[C@@](C)C	CHEMBL1034536	-2.90
26	CHEMBL550766	C(=O)(c2ccc(OCCCCCC)cc2)[C@@](c1ccccc1)C[N@@H+](C)C	CHEMBL1034536	-3.04
27	CHEMBL550767	C(=O)(c2c(Cl)cc(S(=O)(CCCCCC)=O)cc2)CC[N@H+]1CCOCC1	CHEMBL1034536	-3.12
28	CHEMBL550768	C(=O)(c1c(Cl)cc(S(=O)(CCCCCC)=O)cc1)CC[N@H+]2CCN(C(C)=O)CC2	CHEMBL1034536	-3.44
29	CHEMBL550841	C(=O)(c2ccc(OCCCCCC)cc2)CCNc1ccccc1	CHEMBL1034536	-6.70
30	CHEMBL550845	C(=O)(c1c(Cl)cc(OCCCCCC)c(Cl)c1)CC[N@H+](C)C	CHEMBL1034536	-2.98
31	CHEMBL550905	C(=O)(c2c(Cl)cc(OCCCCCC)cc2Cl)CC[N@H+]1CCOCC1	CHEMBL1034536	-6.00
32	CHEMBL550954	C(=O)(c2c(Cl)c(Cl)c(S(=O)(CCCCCC)=O)cc2)CC[N@@H+]1CCOCC1	CHEMBL1034536	-3.25
33	CHEMBL551046	N1(CCC(c2ccc(OCCCCCC)cc2)=O)C(=O)CCC1=O	CHEMBL1034536	-2.89
34	CHEMBL551106	C(=O)(c1c(C)cc(OCCCCCC)cc1C)CC[N@@H+]2CCN(C(C)=O)CC2	CHEMBL1034536	-2.81
35	CHEMBL551184	C(=O)(c1cc(OC)c(OCCCCCC)cc1)CC[N@H+](C)C	CHEMBL1034536	-3.02

36	CHEMBL551185	C(=O)(c1c(OC)cc(OCCCCCC)cc1)CC[N@H+](C)C	CHEMBL1034536	-2.98
37	CHEMBL551244	C(=O)(c1ccc(C[C@]2CC[N@@H+](CC)CC2)cc1)CC[N@@H+](C)C	CHEMBL1034536	-3.85
38	CHEMBL551385	C(=O)(c2c(Cl)c(Cl)c(SCCCCCC)cc2)CC[N@@H+]1CCN(S(=O)(CC)=O)CC1	CHEMBL1034536	-4.52
39	CHEMBL551450	C(=O)(c2ccc(OCCCCCC)cc2)CC[N@@H+]1CC[N@@H+](C)CC1	CHEMBL1034536	-2.64
40	CHEMBL551710	C(=O)(c1cc(OCCCCCC)ccc1)CC[N@H+](C)C	CHEMBL1034536	-3.08
41	CHEMBL551791	C(=O)(c1c(Br)cc(OCCCCCC)cc1)CC[N@@H+](C)C	CHEMBL1034536	-4.10
42	CHEMBL551962	C(=O)(c2c(Cl)cc(OCCCCCC)cc2Cl)CC[N@H+]1[C@](C)C1	CHEMBL1034536	-6.00
43	CHEMBL551975	N1C(=O)C[N@@H+](CCC(c2c(Cl)cc(SCCCCCC)cc2)=O)CC1	CHEMBL1034536	-2.87
44	CHEMBL551976	C(=O)(c2c(Cl)cc(SCCCCCC)cc2)CC[N@@H+]1CCN(S(=O)(CC)=O)CC1	CHEMBL1034536	-3.13
45	CHEMBL551979	C(=O)(c1cc([C@](C)(C)C)c(OCCCCCC)cc1)CC[N@@H+](C)C	CHEMBL1034536	-3.72
46	CHEMBL552048	C(=O)(c1c(Cl)cc(OCCCCCC)c(Cl)c1)CC[N@@H+]2CCN(C(C)=O)CC2	CHEMBL1034536	-2.81
47	CHEMBL552105	C(=O)(c1cc(SC)c(OCCCCCC)cc1)CC[N@H+](C)C	CHEMBL1034536	-2.90
48	CHEMBL552106	C(=O)(c1c(SC)cc(OCCCCCC)cc1)CC[N@H+](C)C	CHEMBL1034536	-2.84
49	CHEMBL552158	C(=O)(c1cc(F)c(OCCCCCC)cc1)CC[N@H+](C)C	CHEMBL1034536	-3.16
50	CHEMBL552185	C(=O)(c1ccc(OCCCCCC)c2ccccc12)CC[N@@H+](C)C	CHEMBL1034536	-3.18
51	CHEMBL552392	C1(=O)[C@](C[N@H+](C)C)CCc2cc(OCCCCCC)ccc12	CHEMBL1034536	-3.15
52	CHEMBL552517	C(=O)(c2c(Cl)cc(SCCCCCC)c(Cl)c2)CC[N@H+]1[C@](C)C1	CHEMBL1034536	-3.85
53	CHEMBL552742	C(=O)(c1c(N(C)C)cc(OCCCCCC)cc1)CC[N@H+](C)C	CHEMBL1034536	-3.77
54	CHEMBL553190	C(=O)(c1ccc(SCCCCCC)cc1)CC[N@H+](C)C	CHEMBL1034536	-2.83
55	CHEMBL553624	C(=O)(c2c(C)cc(OCCCCCC)cc2C)CC[N@H+]1[C@](C)C1	CHEMBL1034536	-3.03
56	CHEMBL553652	C(=O)(c2ccc(OCCCCCC)cc2)CC[N@H+]1CCCC1	CHEMBL1034536	-2.89
57	CHEMBL555065	C(=O)(c2c(Cl)cc(OCCCCCC)c(Cl)c2)CC[N@H+]1[C@](C)C1	CHEMBL1034536	-3.09
58	CHEMBL555726	C(S(=O)(Nc1ccc(C(=O)CC[N@@H+](C)C)cc1)=O)CCCC	CHEMBL1034536	-4.30
59	CHEMBL555749	C1(=O)[C@](C[N@H+](C)C)Cc2cc(OCCCCCC)ccc12	CHEMBL1034536	-3.64
60	CHEMBL556270	C(=O)(c2c(Cl)c(Cl)c(OCCCCCC)cc2)CC[N@@H+]1CCN(S(=O)(CC)=O)CC1	CHEMBL1034536	-6.00
61	CHEMBL557905	C(=O)(c1c(S(=O)(C)=O)cc(OCCCCCC)cc1)CC[N@H+](C)C	CHEMBL1034536	-3.07
62	CHEMBL557906	C(=O)(c2c(Cl)cc(SCCCCCC)cc2Cl)CC[N@@H+]1CCOCC1	CHEMBL1034536	-5.00
63	CHEMBL558106	C(=O)(c1c([C@](F)(F)F)cc(OCCCCCC)cc1)CC[N@H+](C)C	CHEMBL1034536	-3.30
64	CHEMBL558107	C(=O)(c1cc([N+]([O-])=O)c(OCCCCCC)cc1)CC[N@@H+](C)C	CHEMBL1034536	-2.87
65	CHEMBL558297	C(=O)(c2c(Cl)c(Cl)c(S(=O)(CCCCCC)=O)cc2)CC[N@@H+]1CCN(S(=O)(CC)=O)CC1	CHEMBL1034536	-3.42
66	CHEMBL558298	C(=O)(c2c(Cl)cc(SCCCCCC)cc2Cl)CC[N@@H+]1CCN(S(=O)(CC)=O)CC1	CHEMBL1034536	-4.30
67	CHEMBL558889	C(=O)(Nc1ccc(C(=O)CC[N@@H+](C)C)cc1)CCCCC	CHEMBL1034536	-3.25
68	CHEMBL559520	C(=O)(c1c(C)cc(OCCCCCC)cc1)CC[N@H+](C)C	CHEMBL1034536	-3.13
69	CHEMBL559521	C(=O)(c2c(Cl)c(Cl)c(SCCCCCC)cc2)CC[N@H+]1[C@](C)C1	CHEMBL1034536	-4.16
70	CHEMBL559522	C(=O)(c2c(Cl)c(Cl)c(SCCCCCC)cc2)CC[N@@H+]1CCOCC1	CHEMBL1034536	-3.77
71	CHEMBL559716	C(=O)(c1cc(Br)c(OCCCCCC)cc1)CC[N@@H+](C)C	CHEMBL1034536	-3.34
72	CHEMBL559717	C(=O)(c1c(Cl)cc(SCCCCCC)c(Cl)c1)CC[N@H+](C)C	CHEMBL1034536	-3.48
73	CHEMBL559718	N1C(=O)C[N@H+](CCC(c2c(Cl)cc(SCCCCCC)c(Cl)c2)=O)CC1	CHEMBL1034536	-3.09
74	CHEMBL560108	N1C(=O)C[N@H+](CCC(c2c(Cl)cc(S(=O)(CCCCCC)=O)c(Cl)c2)=O)CC1	CHEMBL1034536	-3.27

75	CHEMBL560114	C(=O)(c1c([C@@](C)C)cc(OCCCCCC)c(C)c1)CC[N@H+](C)C	CHEMBL1034536	-3.07
76	CHEMBL560148	C(=O)(c1c(OCCCCCC)cc(C)cc1)CC[N@H+](C)C	CHEMBL1034536	-2.96
77	CHEMBL560172	C(=O)(c1ccc(OCCCCCC)cc1)CC[N@@H+](C)C	CHEMBL1034536	-2.98
78	CHEMBL560309	C(=O)(c2ccc(OCCCCCC)cc2)CCn1cncc1	CHEMBL1034536	-2.83
79	CHEMBL560310	C(=O)(c1ccc(N2CC[N@H+](CCC)CC2)cc1)CC[N@@H+](C)C	CHEMBL1034536	-3.50
80	CHEMBL560311	C(=O)(c1c(Cl)c(Cl)c(OCCCCCC)cc1)CC[N@H+](C)C	CHEMBL1034536	-3.22
81	CHEMBL560369	C(=O)(c1c(Cl)cc(SCCCCCC)cc1)CC[N@@H+]2CCN(C(C)=O)CC2	CHEMBL1034536	-2.85
82	CHEMBL560510	C(=O)(c1ccc(S(=O)(CCCCCC)=O)cc1)CC[N@H+](C)C	CHEMBL1034536	-3.11
83	CHEMBL560512	C(=O)(c1cc(c2ccccc2)c(OCCCCCC)cc1)CC[N@H+](C)C	CHEMBL1034536	-3.08
84	CHEMBL560568	C(=O)(c1c(Cl)c(Cl)c(SCCCCCC)cc1)CC[N@H+](C)C	CHEMBL1034536	-3.85
85	CHEMBL560569	C(=O)(c1c(Cl)c(Cl)c(SCCCCCC)cc1)CC[N@@H+]2CCN(C(C)=O)CC2	CHEMBL1034536	-2.64
86	CHEMBL560675	N(CCCC)C(Nc1ccc(C(=O)CC[N@H+](C)C)cc1)=O	CHEMBL1034536	-5.00
87	CHEMBL560911	C(=O)(c1ccc(OCCCCCC)cc1)CC[N@H+](CCCC)CCCC	CHEMBL1034536	-3.25
88	CHEMBL560972	C(=O)(c1cc([C@](F)(F)F)c(OCCCCCC)cc1)CC[N@H+](C)C	CHEMBL1034536	-3.03
89	CHEMBL561050	C(=O)(c1cc(I)c(OCCCCCC)cc1)CC[N@H+](C)C	CHEMBL1034536	-3.27
90	CHEMBL561051	C(=O)(c2c(Cl)cc(SCCCCCC)c(Cl)c2)CC[N@@H+]1CCN(S(=O)(CC)=O)CC1	CHEMBL1034536	-4.70
91	CHEMBL561052	C(=O)(c1c(Cl)cc(SCCCCCC)cc1Cl)CC[N@H+](C)C	CHEMBL1034536	-3.46
92	CHEMBL561170	C(=O)(c2c(Cl)cc(SCCCCCC)cc2Cl)CC[N@H+]1[C@](C)C1	CHEMBL1034536	-3.72
93	CHEMBL561245	C(=O)(c2ccc(OCCCCCC)cc2)CC[N@@H+]1CCOCC1	CHEMBL1034536	-2.75
94	CHEMBL561446	C(=O)(c1c(Cl)cc(S(=O)(CCCCCC)=O)cc1Cl)CC[N@@H+](C)C	CHEMBL1034536	-3.77
95	CHEMBL561569	C(=O)(c1c(Cl)cc(S(=O)(CCCCCC)=O)c(Cl)c1)CC[N@@H+](C)C	CHEMBL1034536	-4.05
96	CHEMBL561570	C(=O)(c2c(Cl)cc(S(=O)(CCCCCC)=O)c(Cl)c2)CC[N@@H+]1CCOCC1	CHEMBL1034536	-3.17
97	CHEMBL561573	C(=O)(c2c(Cl)cc(S(=O)(CCCCCC)=O)cc2)CC[N@H+]1[C@](C)C1	CHEMBL1034536	-3.80
98	CHEMBL561574	C(=O)(c2c(Cl)cc(S(=O)(CCCCCC)=O)cc2)CC[N@@H+]1CCN(S(=O)(CC)=O)CC1	CHEMBL1034536	-3.52
99	CHEMBL561648	C(=O)(NCCCCCC)c1ccc(C(=O)CC[N@@H+](C)C)cc1	CHEMBL1034536	-3.11
100	CHEMBL561649	C(=O)(c1c(C)cc(S(=O)(CCCCCC)=O)cc1C)CC[N@H+](C)C	CHEMBL1034536	-2.73
101	CHEMBL561650	C(=O)(c2c(Cl)c(Cl)c(OCCCCCC)cc2)CC[N@H+]1[C@](C)C1	CHEMBL1034536	-3.43
102	CHEMBL561653	C(=O)(c1ccc(c2ccc(OCCCCCC)cc2)cc1)CC[N@@H+](C)C	CHEMBL1034536	-3.82
103	CHEMBL561850	c1c(CCCCCCC)ccc(C(=O)CC[N@H+](C)C)c1	CHEMBL1034536	-2.81
104	CHEMBL561975	C(=O)(c1c(Cl)c(Cl)c(S(=O)(CCCCCC)=O)cc1)CC[N@@H+]2CCN(C(C)=O)CC2	CHEMBL1034536	-3.11
105	CHEMBL562173	C(=O)(c2c(C)cc(S(=O)(CCCCCC)=O)cc2C)CC[N@H+]1[C@](C)C1	CHEMBL1034536	-2.92
106	CHEMBL562250	C(=O)(c2ccc(OCCCCCC)cc2)CC[N@H+]1[C@](C)C1	CHEMBL1034536	-2.78
107	CHEMBL562251	C(=O)(c2c(Cl)c(Cl)c(S(=O)(CCCCCC)=O)cc2)CC[N@H+]1[C@](C)C1	CHEMBL1034536	-3.36
108	CHEMBL562507	C(=O)(c2c(Cl)cc(SCCCCCC)cc2)CC[N@H+]1[C@](C)C1	CHEMBL1034536	-3.30
109	CHEMBL563032	C(=O)(c1c(C)cc(S(=O)(CCCCCC)=O)cc1C)CC[N@@H+]2CCN(C(C)=O)CC2	CHEMBL1034536	-3.33
110	CHEMBL563154	C(=O)(c2c(Cl)cc(SCCCCCC)c(Cl)c2)CC[N@@H+]1CCOCC1	CHEMBL1034536	-2.88
111	CHEMBL563175	C(=O)(c1c(Cl)cc(S(=O)(CCCCCC)=O)c(Cl)c1)CC[N@@H+]2CCN(C(C)=O)CC2	CHEMBL1034536	-3.17
112	CHEMBL563181	C(=O)(c1c(Cl)cc(OCCCCCC)cc1)CC[N@@H+](C)C	CHEMBL1034536	-2.71
113	CHEMBL563566	C(=O)(c2c(Cl)cc(S(=O)(CCCCCC)=O)c(Cl)c2)CC[N@@H+]1CCN(S(=O)(CC)=O)CC1	CHEMBL1034536	-3.14

114	CHEMBL563804	C(=O)(c1c(Cl)cc(SCCCCCC)c(Cl)c1)CC[N@H+]2CCN(C(C)=O)CC2	CHEMBL1034536	-3.00
115	CHEMBL563816	C(=O)(c1c(Cl)cc(S(=O)(CCCCCC)=O)cc1Cl)CC[N@@H+]2CCOCC2	CHEMBL1034536	-3.33
116	CHEMBL563871	C(=O)(c2c(Cl)cc(OCCCCCC)cc2Cl)CC[N@@H+]1CCN(S(=O)(CC)=O)CC1	CHEMBL1034536	-3.07
117	CHEMBL563943	C(=O)(c1c(Cl)cc(S(=O)(CCCCCC)=O)cc1Cl)CC[N@H+]2[C@](C)C2	CHEMBL1034536	-3.01
118	CHEMBL564112	N1C(=O)C[N@H+](CCC(c2c(Cl)cc(S(=O)(CCCCCC)=O)cc2)=O)CC1	CHEMBL1034536	-3.62
119	CHEMBL564464	C(=O)(c1c(Cl)cc(S(=O)(CCCCCC)=O)cc1Cl)CC[N@H+]2CC(=O)NCC2	CHEMBL1034536	-3.37
120	CHEMBL565150	C(=O)(c1c(I)cc(OCCCCCC)cc1)CC[N@@H+](C)C	CHEMBL1034536	-3.57
121	CHEMBL569845	N1C(=O)C[N@H+](CCC(c2c(Cl)c(Cl)c(SCCCCCC)cc2)=O)CC1	CHEMBL1034536	-2.80
122	CHEMBL570307	C(=O)(c1ccc(OCCCCCC)cc1)[C@@](C)C[N@H+](C)C	CHEMBL1034536	-2.87
123	CHEMBL570776	C(=O)(c2c(Cl)cc(OCCCCCC)cc2)CC[N@H+]1[C@](C)C1	CHEMBL1034536	-3.06
124	CHEMBL571226	N1C(=O)C[N@@H+](CCC(c2c(Cl)cc(OCCCCCC)cc2Cl)=O)CC1	CHEMBL1034536	-2.86
125	CHEMBL572341	C(=O)(c2c(Cl)cc(S(=O)(CCCCCC)=O)c(Cl)c2)CC[N@H+]1[C@](C)C1	CHEMBL1034536	-3.59
126	CHEMBL572342	C(=O)(c2c(C)cc(SCCCCCC)cc2C)CC[N@H+]1[C@](C)C1	CHEMBL1034536	-3.11
127	CHEMBL572348	C(=O)(c1cc(Cl)c(OCCCCCC)cc1)CC[N@H+](C)C	CHEMBL1034536	-2.99
128	CHEMBL1294	[N@H+]12[C@](C[C@@](CC2)[C@@](C=C)C1)[C@@](O)c4c3c(ccc(OC)c3)ncc4	CHEMBL3430218	-5.48
129	CHEMBL24	C(Cc1ccc(cc1)OC[C@](O)C[NH2+][C@@](C)C)(N)=O	CHEMBL3430218	-7.52
130	CHEMBL3221410	N1[C@@](C[C@@](C)C)C(=O)N2[C@@](C(=O)N(C)[C@@](Cc3ccc(O)cc3)C(=O)N[C@@](C[C@@](C)C)C(=O)N(C)[C@](C[C@@](C)C)C(=O)N(C)[C@@](C[C@@](C)C)C1=O)CCC2	CHEMBL3430218	-6.24
131	CHEMBL3342578	N3[C@@](C[C@@](C)C)C(N[C@](C[C@@](C)C)C(N[C@@](C[C@@](C)C)C(N[C@@](C[C@@](C)C)C(N1[C@](CCC1)C(N[C@@](Cc2ccc(O)cc2)C3=O)=O)=O)=O)=O)=O	CHEMBL3430218	-5.91
132	CHEMBL3403637	[C@]2(C[C@@](C)C)C(=O)N[C@@](C[C@@](C)C)C(=O)N1[C@](C(=O)N[C@@](Cc3ccc(O)cc3)C(=O)N[C@](C[C@@](C)C)C(=O)N[C@@](C[C@@](C)C)C(=O)N2)CCC1	CHEMBL3430218	-6.23
133	CHEMBL3425512	[C@]2(CCCC[NH3+])NC(=O)[C@](NC(=O)[C@@](NC(=O)[C@@]1CCCN1C(=O)[C@](NC(=O)[C@]([C@](O)C)NC2=O)Cc5ccccc5)Cc6ccccc6)Cc3cnc4c3cccc4	CHEMBL3430218	-7.05
134	CHEMBL3425617	[C@]3(C[C@@](C)C)N(C)C(=O)[C@](N(C)C(=O)[C@@](NC(=O)[C@@]2CCCN2C(=O)[C@](NC(=O)[C@](C[C@@](C)C)NC3=O)C[C@@](C)C)Cc1ccc(O)cc1)C[C@@](C)C	CHEMBL3430218	-6.17
135	CHEMBL3425618	[C@@]3(C[C@@](C)C)N(C)C(=O)[C@](N(C)C(=O)[C@@](NC(=O)[C@@]2CCCN2C(=O)[C@@](N(C)C(=O)[C@](C[C@@](C)C)NC3=O)C[C@@](C)C)Cc1ccc(O)cc1)C[C@@](C)C	CHEMBL3430218	-5.64
136	CHEMBL3425619	[C@]3(C[C@@](C)C)NC(=O)[C@@](NC(=O)[C@@](NC(=O)[C@]2CCCN2C(=O)[C@](NC(=O)[C@](C[C@@](C)C)NC3=O)C[C@@](C)C)Cc1ccc(O)cc1)C[C@@](C)C	CHEMBL3430218	-6.96
137	CHEMBL3425620	[C@]2(C[C@@](C)C)NC(=O)[C@@](NC(=O)[C@@](NC(=O)[C@]1CCCN1C(=O)[C@](NC(=O)[C@@](C[C@@](C)C)NC2=O)C[C@@](C)C)Cc3cnc4c3cccc4)C[C@@](C)C	CHEMBL3430218	-5.48
138	CHEMBL3425621	[C@@]2(C[C@@](C)C)NC(=O)[C@](C[C@@](C)C)NC(=O)[C@@](NC(=O)[C@@]1CCCN1C(=O)[C@@]3N(CCC3)C(=O)[C@@](C[C@@](C)C)NC2=O)Cc4cnc5c4cccc5	CHEMBL3430218	-6.72
139	CHEMBL3425622	[C@]2(C[C@@](C)C)NC(=O)[C@](NC(=O)[C@@](NC(=O)[C@@]1CCCN1C(=O)[C@@](NC(=O)[C@@](C[C@@](C)C)NC2=O)C[C@@](C)C)Cc3cnc4c3cccc4)C[C@@](C)C	CHEMBL3430218	-6.55

140	CHEMBL3425623	[C@]2(C[C@@](C)C)NC(=O)[C@](NC(=O)[C@@](NC(=O)[C@@]1CCCN1C(=O)[C@](NC(=O)[C@](C[C@@](C)C)NC2=O)C[C@@](C)C)Cc3cnc4c3cccc4)C[C@@](C)C	CHEMBL3430218	-5.60
141	CHEMBL3425624	[C@]2(C[C@@](C)C)NC(=O)[C@@](NC(=O)[C@@](NC(=O)[C@]1CCCN1C(=O)[C@@](NC(=O)[C@](C[C@@](C)C)NC2=O)C[C@@](C)C)Cc3cnc4c3cccc4)C[C@@](C)C	CHEMBL3430218	-5.78
142	CHEMBL3425625	[C@]2(C[C@@](C)C)NC(=O)[C@@](NC(=O)[C@@](NC(=O)[C@]1CCCN1C(=O)[C@@](NC(=O)[C@]([C@](CC)C)NC2=O)[C@](CC)C)Cc3cnc4c3cccc4)[C@@](C)CC	CHEMBL3430218	-6.39
143	CHEMBL3425626	C2NC(=O)[C@@](NC(=O)[C@@](NC(=O)[C@]1CCCN1C(=O)[C@@](NC(=O)[C@](Cc3ccccc3)NC2=O)C[C@@](C)C)Cc4ccc(O)cc4)C	CHEMBL3430218	-8.00
144	CHEMBL3425627	C2NC(=O)[C@](NC(=O)[C@@](NC(=O)[C@@]1CCCN1C(=O)[C@](NC(=O)[C@](Cc3ccccc3)NC2=O)C[C@@](C)C)Cc4ccc(O)cc4)C	CHEMBL3430218	-7.16
145	CHEMBL3425628	C1NC(=O)[C@](NC(=O)[C@@](NC(=O)[C@@](NC(=O)[C@](NC(=O)[C@](Cc2ccccc2)NC1=O)C[C@@](C)C)C[C@@](C)C)Cc3ccc(O)cc3)C	CHEMBL3430218	-7.70
146	CHEMBL3425629	C2NC(=O)[C@@](NC(=O)[C@@](NC(=O)[C@@]1CCCN1C(=O)[C@](NC(=O)[C@@](Cc3ccccc3)NC2=O)C[C@@](C)C)Cc4ccc(O)cc4)C	CHEMBL3430218	-8.00
147	CHEMBL3425630	C2NC(=O)[C@](NC(=O)[C@@](NC(=O)[C@@]1CCCN1C(=O)[C@@](NC(=O)[C@@](Cc3ccccc3)NC2=O)C[C@@](C)C)Cc4ccc(O)cc4)C	CHEMBL3430218	-8.00
148	CHEMBL3425631	C2NC(=O)[C@](NC(=O)[C@@](NC(=O)[C@@]1CCCN1C(=O)[C@@](NC(=O)[C@](Cc3ccccc3)NC2=O)C[C@@](C)C)Cc4ccc(O)cc4)C	CHEMBL3430218	-7.52
149	CHEMBL3425632	C2N(C)C(=O)[C@@](NC(=O)[C@@](N(C)C(=O)[C@]1CCCN1C(=O)[C@@](NC(=O)[C@](Cc3ccccc3)N(C)C2=O)C[C@@](C)C)Cc4ccc(O)cc4)	CHEMBL3430218	-6.46
150	CHEMBL3425633	C2NC(=O)[C@@](NC(=O)[C@@](N(C)C(=O)[C@]1CCCN1C(=O)[C@@](N(C)C(=O)[C@](Cc3ccccc3)N(C)C2=O)C[C@@](C)C)Cc4ccc(O)cc4)	CHEMBL3430218	-6.80
151	CHEMBL3425634	C2N(C)C(=O)[C@](N(C)C(=O)[C@@](NC(=O)[C@@]1CCCN1C(=O)[C@](NC(=O)[C@](Cc3ccccc3)NC2=O)C[C@@](C)C)Cc4ccc(O)cc4)C	CHEMBL3430218	-7.16
152	CHEMBL3425635	C1N(C)C(=O)[C@](N(C)C(=O)[C@@](NC(=O)[C@](C[C@@](C)C)N(C)C(=O)[C@](N(C)C(=O)[C@](Cc2ccccc2)NC1=O)C[C@@](C)C)Cc3ccc(O)cc3)C	CHEMBL3430218	-6.03
153	CHEMBL3425636	C2N(C)C(=O)[C@@](N(C)C(=O)[C@@](NC(=O)[C@@]1CCCN1C(=O)[C@](NC(=O)[C@@](Cc3ccccc3)NC2=O)C[C@@](C)C)Cc4ccc(O)cc4)	CHEMBL3430218	-7.52
154	CHEMBL3425637	C2N(C)C(=O)[C@](N(C)C(=O)[C@@](NC(=O)[C@@]1CCCN1C(=O)[C@@](N(C)C(=O)[C@@](Cc3ccccc3)NC2=O)C[C@@](C)C)Cc4ccc(O)cc4)C	CHEMBL3430218	-6.85
155	CHEMBL3425639	C2N(C)C(=O)[C@@](N(C)C(=O)[C@@](NC(=O)[C@@]1CCCN1C(=O)[C@@](NC(=O)[C@](Cc3ccccc3)NC2=O)C[C@@](C)C)Cc4ccc(O)cc4)C[C@@](C)C	CHEMBL3430218	-7.00
156	CHEMBL3425640	[C@]2(C[C@@](C)C)N(C)C(=O)[C@@](N(C)C(=O)[C@@](NC(=O)[C@@]1CCCN1C(=O)[C@@](NC(=O)[C@](Cc3ccccc3)NC2=O)C[C@@](C)C)Cc4ccc(O)cc4)C[C@@](C)C	CHEMBL3430218	-6.39
157	CHEMBL3425642	[C@@]1(C)NC(=O)[C@](NC(=O)[C@@](NC(=O)[C@@](N(C)C(=O)[C@@](NC(=O)[C@](C)N(C)C1=O)C)C)C)C	CHEMBL3430218	-8.00
158	CHEMBL3425643	[C@@]1(Cc2ccccc2)NC([C@]6N(CCC6)C(=O)[C@@](NC(=O)[C@@](NC(=O)[C@@](NC(=O)[C@](Cc3ccccc3)NC1=O)Cc4cnc5c4cccc5)[C@@](C)C)C[C@@](C)C)=O	CHEMBL3430218	-6.43
159	CHEMBL3425644	[C@]12CCCN2C(=O)[C@](Cc4ccc(O)cc4)NC(=O)[C@](Cc3ccccc3)NC([C@]5N(CCC5)C(=O)[C@@](NC(=O)CNC1=O)C[C@@](C)C)=O	CHEMBL3430218	-8.00
160	CHEMBL3425646	[C@]1(C[C@@](C)C)C(=O)N[C@@](Cc3ccc(O)cc3)C(=O)N[C@@]([C@@](C)C)C(NCC(=O)N[C@@]([C@](O)C)C(N[C@@](Cc2ccccc2)C(N1)=O)=O)=O	CHEMBL3430218	-7.40
161	CHEMBL3425647	[C@]1(Cc3ccccc3)C(=O)N[C@@](Cc2ccc(O)cc2)C(=O)N[C@]([C@](CC)C)C(NCC(=O)N[C@@]([C@](C)C)C(NCC(N1)=O)=O)=O	CHEMBL3430218	-8.00
162	CHEMBL3425648	[C@]1(C[C@@](C)C)C(=O)N[C@@](Cc2ccc(O)cc2)C(=O)N[C@@](C)C(NCC(=O)N[C@@]([C@](O)C)C(N[C@@](Cc3ccccc3)C(N1)=O)=O)=O	CHEMBL3430218	-7.70
163	CHEMBL3425649	[C@]1(Cc3ccccc3)C(=O)NCC(=O)N[C@@](C[C@@](C)C)C(N[C@@](C)C(=O)N[C@@](Cc2ccc(O)cc2)C(N[C@@](CC(=O)N)C(N1)=O)=O)=O	CHEMBL3430218	-8.00
164	CHEMBL3425650	[C@]1(C[C@@](C)C)C(=O)N[C@@](Cc3ccc(O)cc3)C(=O)N[C@]([C@](O)C)C(NCC(=O)N[C@@]([C@](O)C)C(N[C@@](Cc2ccccc2)C(N1)=O)=O)=O	CHEMBL3430218	-7.70

165	CHEMBL3425653	C2C(=O)N[C@@]([C@](O)C)C(N[C@@](Cc1ccccc1)C(N[C@@](C[C@@](C)C)C(=O)N[C@@](Cc3ccc(O)cc3)C(=O)N[C@@]([C@](O)C)C(N2C)=O)=O)=O	CHEMBL3430218	-7.70
166	CHEMBL3425654	C2C(=O)N(C)[C@]([C@](O)C)C(N[C@@](Cc1ccccc1)C(N[C@@](C[C@@](C)C)C(=O)N[C@@](Cc3ccc(O)cc3)C(=O)N[C@@]([C@](O)C)C(N2C)=O)=O)=O	CHEMBL3430218	-8.00
167	CHEMBL3427784	[C@]3(C[C@@](C)C)NC(=O)[C@](C[C@@](C)C)NC(=O)[C@@](NC(=O)[C@@]2CCCN2C(=O)[C@](NC(=O)[C@@](C[C@@](C)C)NC3=O)C[C@@](C)C)Cc1ccc(O)cc1	CHEMBL3430218	-5.79
168	CHEMBL3427785	C[C@@](C)C[C@@]3NC(=O)[C@](C[C@@](C)C)NC(=O)[C@@](NC(=O)[C@@]2CCCN2C(=O)[C@@]4N(CCC4)C(=O)[C@@](C[C@@](C)C)NC3=O)Cc1ccc(O)cc1	CHEMBL3430218	-7.05
169	CHEMBL3427786	[C@]3(C[C@@](C)C)NC(=O)[C@@](C[C@@](C)C)NC(=O)[C@@](NC(=O)[C@@]2CCCN2C(=O)[C@@](NC(=O)[C@@](C[C@@](C)C)NC3=O)C[C@@](C)C)Cc1ccc(O)cc1	CHEMBL3430218	-6.96
170	CHEMBL3427787	[C@]3(C[C@@](C)C)NC(=O)[C@@](C[C@@](C)C)NC(=O)[C@@](NC(=O)[C@@]2CCCN2C(=O)[C@](NC(=O)[C@](C[C@@](C)C)NC3=O)C[C@@](C)C)Cc1ccc(O)cc1	CHEMBL3430218	-5.46
171	CHEMBL3427788	[C@]3(C[C@@](C)C)N(C)C(=O)[C@](C[C@@](C)C)NC(=O)[C@@](NC(=O)[C@]2CCCN2C(=O)[C@@](NC(=O)[C@](C[C@@](C)C)NC3=O)C[C@@](C)C)Cc1ccc(O)cc1	CHEMBL3430218	-6.11
172	CHEMBL3427789	[C@]3(C[C@@](C)C)NC(=O)[C@](C[C@@](C)C)NC(=O)[C@@](NC(=O)[C@]2CCCN2C(=O)[C@@](NC(=O)[C@](C[C@@](C)C)N(C)C3=O)C[C@@](C)C)Cc1ccc(O)cc1	CHEMBL3430218	-6.12
173	CHEMBL3427790	[C@]3(C[C@@](C)C)NC(=O)[C@](C[C@@](C)C)NC(=O)[C@@](N(C)C(=O)[C@]2CCCN2C(=O)[C@@](NC(=O)[C@](C[C@@](C)C)NC3=O)C[C@@](C)C)Cc1ccc(O)cc1	CHEMBL3430218	-5.89
174	CHEMBL3427791	[C@]3(C[C@@](C)C)N(C)C(=O)[C@@](NC(=O)[C@@](NC(=O)[C@]2CCCN2C(=O)[C@@](NC(=O)[C@](C[C@@](C)C)N(C)C3=O)C[C@@](C)C)Cc1ccc(O)cc1)C[C@@](C)C	CHEMBL3430218	-6.02
175	CHEMBL3427792	[C@]3(C[C@@](C)C)N(C)C(=O)[C@@](NC(=O)[C@@](N(C)C(=O)[C@]2CCCN2C(=O)[C@@](NC(=O)[C@](C[C@@](C)C)NC3=O)C[C@@](C)C)Cc1ccc(O)cc1)C[C@@](C)C	CHEMBL3430218	-5.82
176	CHEMBL3427793	[C@]3(C[C@@](C)C)NC(=O)[C@@](NC(=O)[C@@](N(C)C(=O)[C@]2CCCN2C(=O)[C@@](NC(=O)[C@](C[C@@](C)C)N(C)C3=O)C[C@@](C)C)Cc1ccc(O)cc1)C[C@@](C)C	CHEMBL3430218	-5.88
177	CHEMBL3427794	[C@]3(C[C@@](C)C)N(C)C(=O)[C@@](NC(=O)[C@@](NC(=O)[C@@]2CCCN2C(=O)[C@](NC(=O)[C@@](C[C@@](C)C)N(C)C3=O)C[C@@](C)C)Cc1ccc(O)cc1)C[C@@](C)C	CHEMBL3430218	-5.42
178	CHEMBL3427795	[C@@]3(C[C@@](C)C)N(C)C(=O)[C@](C[C@@](C)C)N(C)C(=O)[C@@](NC(=O)[C@@]2CCCN2C(=O)[C@@]4N(CCC4)C(=O)[C@@](C[C@@](C)C)NC3=O)Cc1ccc(O)cc1	CHEMBL3430218	-5.96
179	CHEMBL3427796	[C@]3(C[C@@](C)C)N(C)C(=O)[C@](N(C)C(=O)[C@@](NC(=O)[C@@]2CCCN2C(=O)[C@@](N(C)C(=O)[C@@](C[C@@](C)C)NC3=O)C[C@@](C)C)Cc1ccc(O)cc1)C[C@@](C)C	CHEMBL3430218	-5.73
180	CHEMBL3427797	[C@]3(C[C@@](C)C)N(C)C(=O)[C@](N(C)C(=O)[C@@](NC(=O)[C@@]2CCCN2C(=O)[C@](N(C)C(=O)[C@](C[C@@](C)C)NC3=O)C[C@@](C)C)Cc1ccc(O)cc1)C[C@@](C)C	CHEMBL3430218	-7.00
181	CHEMBL3427798	[C@]3(C[C@@](C)C)N(C)C(=O)[C@@](N(C)C(=O)[C@@](NC(=O)[C@]2CCCN2C(=O)[C@@](N(C)C(=O)[C@](C[C@@](C)C)N(C)C3=O)C[C@@](C)C)Cc1ccc(O)cc1)C[C@@](C)C	CHEMBL3430218	-5.82

182	CHEMBL421362	N13C(=O)[C@](Cc5ccccc5)NC(=O)[C@]([C@@](C)O)NC(=O)[C@](CCCC[NH3+])NC(=O)[C@@](Cc2cnc4c2cccc4)NC(=O)[C@](Cc6ccccc6)NC(=O)[C@@]1CCC3	CHEMBL3430218	-7.10
183	CHEMBL121	N1C(=O)S[C@@](Cc2ccc(OCCN(C)c3ncccc3)cc2)C1=O	CHEMBL3431937	-5.12
184	CHEMBL2216774	c1cccc(OCC(N2[C@@](c3scc(c4ncccc4)n3)CCCC2)=O)c1	CHEMBL3431937	-5.04
185	CHEMBL2216778	c1c(C(=O)Nc3cc(F)ccc3)cc2c(O[C@@](C(=O)[O-])CN2S(=O)(=O)c4cc(Cl)ccc4OC)c1	CHEMBL3431937	-6.85
186	CHEMBL2216779	c1c(C(=O)[O-])ccc(NC(=O)c2cc3c(CCN3S(=O)(=O)c4cc(Cl)ccc4OC)cc2)c1	CHEMBL3431937	-5.56
187	CHEMBL3431460	c1ccc(OCC(N2CCCC[C@@]2c3nc(c4ccc5c(NC(C5)=O)c4)no3)=O)cc1	CHEMBL3431937	-5.84
188	CHEMBL3431462	c1ccc(OCC(N2CCCC[C@@]2C(N4Cc3ccc(Cl)cc3C4)=O)=O)cc1	CHEMBL3431937	-5.30
189	CHEMBL3431464	Cc1c(c2nc([C@]3CCCCN3C(COc4ccccc4)=O)on2)snn1	CHEMBL3431937	-5.23
190	CHEMBL3431465	CCn1c(C)nc(Cl)c1c2nc([C@]3CCCCN3C(COc4ccccc4)=O)on2	CHEMBL3431937	-5.40
191	CHEMBL3431466	Cn1cnc(Br)c1c2nc([C@]3CCCCN3C(COc4ccccc4)=O)on2	CHEMBL3431937	-5.25
192	CHEMBL3431467	Cc1cc(c2nc([C@]3CCCCN3C(COc4ccccc4)=O)on2)n(C)n1	CHEMBL3431937	-5.36
193	CHEMBL3431468	Cc1c(Cl)c(c2nc([C@]3CCCCN3C(COc4ccccc4)=O)on2)n(C)n1	CHEMBL3431937	-5.78
194	CHEMBL3431469	Cc1cc(c2nc([C@]3CCCCN3C(COc4ccccc4)=O)on2)no1	CHEMBL3431937	-5.41
195	CHEMBL3431470	COc1ccc(Cl)cc1S(=O)(N2COc3c2cc(C(Nc4nc(CC([O-])=O)cs4)=O)cc3)=O	CHEMBL3431937	-5.53
196	CHEMBL3431471	COc1ccc(Cl)cc1S(=O)(N2COc3c2cc(C(Nc4ccc(CC([O-])=O)cc4)=O)cc3)=O	CHEMBL3431937	-5.52
197	CHEMBL3431473	c1ccc(OCC(N2CCCC[C@@]2c3nc([C@@]4CCCCC4)on3)=O)cc1	CHEMBL3431937	-6.02
198	CHEMBL3431474	CO[C@]1CC[C@@](c2nc([C@]3CCCCN3C(COc4ccccc4)=O)no2)CC1	CHEMBL3431937	-5.46
199	CHEMBL3431476	COC([C@]1CCCC[C@@]1c2nc([C@]3CCCCN3C(COc4ccccc4)=O)no2)=O	CHEMBL3431937	-5.65
200	CHEMBL3431477	CCn1cc(Sc4cc(Cl)ccc4OC)c2c1ccc(C(Nc3ccc(C([O-])=O)cc3)=O)c2	CHEMBL3431937	-6.15
201	CHEMBL3431478	CCn1cc(S(=O)(c4cc(Cl)ccc4OC)=O)c2c1ccc(C(Nc3ccc(C([O-])=O)cc3)=O)c2	CHEMBL3431937	-6.17
202	CHEMBL3431479	C[C@@]1(C)C(N(c2ccc(Cl)c(c3nc([C@]4CCCCN4C(COc5ccccc5)=O)no3)c2)C(N1)=O)=O	CHEMBL3431937	-6.31
203	CHEMBL3431480	c1ccc(OCC(N2CCCC[C@@]2c3nc(c4c(Cl)cnc(N)n4)on3)=O)cc1	CHEMBL3431937	-5.45
204	CHEMBL3431481	c1ccc(OCC(N2CCCC[C@@]2c3nc(c4ccc(n5cncn5)cc4)on3)=O)cc1	CHEMBL3431937	-5.65
205	CHEMBL3431482	c1ccc(OCC(N2CCCC[C@@]2c3nc(c4ccc(n5cnnn5)cc4)on3)=O)cc1	CHEMBL3431937	-5.77
206	CHEMBL3431483	c1ccc(OCC(N2CCCC[C@@]2c3nc(c4cccc5c4ncn5)on3)=O)cc1	CHEMBL3431937	-5.78
207	CHEMBL3431484	CC(c1ccc(c2nc([C@]3CCCCN3C(COc4ccccc4)=O)no2)cc1)=O	CHEMBL3431937	-5.81
208	CHEMBL3431485	c1ccc(OCC(N2CCCC[C@@]2c3nc(c5cnc4ccccc4n5)on3)=O)cc1	CHEMBL3431937	-5.38
209	CHEMBL3431486	CS(=O)(c1cccc(c2nc([C@]3CCCCN3C(COc4ccccc4)=O)no2)c1)=O	CHEMBL3431937	-5.53
210	CHEMBL3431487	c1ccc(OCC(N2CCCC[C@@]2c3nc(c4ccc(Cl)nc4)on3)=O)cc1	CHEMBL3431937	-5.29
211	CHEMBL3431488	c1ccc(OCC(N2CCCC[C@@]2c3nc(c4ccc5c(scn5)c4)on3)=O)cc1	CHEMBL3431937	-5.62
212	CHEMBL3431490	c1ccc(OCC(N2CCCC[C@@]2c3nc(c4ccc([C@](F)(F)F)nc4)on3)=O)cc1	CHEMBL3431937	-5.66
213	CHEMBL3431491	c1ccc(OCC(N2CCCC[C@@]2c3nc(c4ccc5c(nns5)c4)on3)=O)cc1	CHEMBL3431937	-5.62
214	CHEMBL3431492	c1ccc(OCC(N2CCCC[C@@]2c3nc(c5ccc4cccnc4n5)on3)=O)cc1	CHEMBL3431937	-5.27
215	CHEMBL3431493	c1ccc(OCC(N2CCCC[C@@]2c3nc(c5ccc4cnccc4n5)on3)=O)cc1	CHEMBL3431937	-5.35
216	CHEMBL3431494	c1ccc(OCC(N2CCCC[C@@]2c3nc(c4cnnc5c4cccc5)on3)=O)cc1	CHEMBL3431937	-5.44
217	CHEMBL3431495	c1ccc(OCC(N2CCCC[C@@]2c3nc(c4ccc5c(nnn5)c4)on3)=O)cc1	CHEMBL3431937	-5.67
218	CHEMBL3431496	c1ccc(OCC(N2CCCC[C@@]2c3nnc(c4cccc(N5C(CCC5=O)=O)c4)n3)=O)cc1	CHEMBL3431937	-5.48
219	CHEMBL3431497	COc1ccc(Cl)cc1S(=O)(N2CCOc3c2cc(C(Nc4ccccc4)=O)cn3)=O	CHEMBL3431937	-5.36

220	CHEMBL3431498	C[C@]1CN(S(=O)(c4cc(Cl)ccc4OC)=O)c2c1ccc(C(Nc3ccc(C([O-])=O)cc3)=O)c2	CHEMBL3431937	-5.55
221	CHEMBL3431499	C[C@@]1CN(S(=O)(c4cc(Cl)ccc4OC)=O)c2c1ccc(C(Nc3ccc(C([O-])=O)cc3)=O)c2	CHEMBL3431937	-5.54
222	CHEMBL3431500	c1ccc(OCC(N2CCOC[C@@]2c3nc(c4ccc5c(ncn5)c4)no3)=O)cc1	CHEMBL3431937	-5.55
223	CHEMBL3431501	COc1ccc(Cl)cc1S(=O)(N2CCOc3c2cc(C(Nc4ccc(C([O-])=O)cc4)=O)cn3)=O	CHEMBL3431937	-6.13
224	CHEMBL3431502	c1ccc(OCC(N2CCCC[C@@]2c3nc(c4ccc(N)nc4)no3)=O)cc1	CHEMBL3431937	-5.35
225	CHEMBL3431503	c1ccc(OCC(N2CCCC[C@@]2c3nc(c4ccc5c(ncn5)c4)on3)=O)cc1	CHEMBL3431937	-5.52
226	CHEMBL3431504	COc1ccc(Cl)cc1S(=O)(N2CCS(=O)(c3c2cc(C(Nc4ccc(C([O-])=O)cc4)=O)cc3)=O)=O	CHEMBL3431937	-5.72
227	CHEMBL3431505	COc1ccc(Cl)cc1S(=O)(N2CCOc3c2cc(C(Nc4ccccc4)=O)cc3)=O	CHEMBL3431937	-5.90
228	CHEMBL3431506	C[C@@]2(C)c1ccc(C(Nc4ccc(C([O-])=O)cc4)=O)cc1N(Cc3cc(Cl)ccc3OC)C(O2)=O	CHEMBL3431937	-5.74
229	CHEMBL3431507	C[C@@]2(C)c1ccc(C(Nc4nc(CC([O-])=O)cs4)=O)cc1N(Cc3cc(Cl)ccc3OC)C(O2)=O	CHEMBL3431937	-5.71
230	CHEMBL3431508	CC(Nc1cccc(n2nc([C@]3CCCCN3C(COc4ccccc4)=O)nn2)c1)=O	CHEMBL3431937	-5.46
231	CHEMBL3431509	CC(NCc1cccc(c2nc([C@]3CCCCN3C(COc4ccccc4)=O)no2)c1)=O	CHEMBL3431937	-5.53
232	CHEMBL3431510	CS(=O)(NCc1cccc(c2nc([C@]3CCCCN3C(COc4ccccc4)=O)no2)c1)=O	CHEMBL3431937	-5.75
233	CHEMBL3431511	COc1ccc(Cl)cc1S(=O)(N2CCOc3c2cc(C(Nc4ccc(CC([O-])=O)cc4)=O)cc3F)=O	CHEMBL3431937	-5.55
234	CHEMBL3431512	COc1ccc(Cl)cc1S(=O)(N2CCOc3c2cc(C(Nc4nc(CC([O-])=O)cs4)=O)cc3F)=O	CHEMBL3431937	-5.61
235	CHEMBL3431513	COc1ccc2c(c(C(COc3ccncc3)=O)cn2)c1	CHEMBL3431937	-7.70
236	CHEMBL3431514	c1cc(C(Nc3ccc(c4nnnn4)cc3)=O)cc(NS(=O)(c2cccc(Cl)c2)=O)c1	CHEMBL3431937	-7.56
237	CHEMBL3431515	c1ccc(OCC(N2CCCC[C@@]2c3nc(c4ccc(C(N)=O)cc4)no3)=O)cc1	CHEMBL3431937	-5.29
238	CHEMBL3431516	c1ccc(OCC(N2CCCC[C@@]2c3nc(c4cccc(S(=O)(N)=O)c4)no3)=O)cc1	CHEMBL3431937	-5.79
239	CHEMBL3431517	c1ccc(OCC(N2CCCC[C@@]2c3nc(c4cccc(C([O-])=O)c4)no3)=O)cc1	CHEMBL3431937	-5.72
240	CHEMBL3431518	c1cc(C(Nc4ccc(C([O-])=O)cc4)=O)cc(S(=O)(N2CCCc3c2cc([C@](F)(F)F)cc3)=O)c1	CHEMBL3431937	-5.44
241	CHEMBL3431519	CCN(C(c1ccc(c2nc([C@]3CCCCN3C(COc4ccccc4)=O)on2)cc1)=O)CC	CHEMBL3431937	-5.83
242	CHEMBL3431520	Cc1cc(C(Nc3ccc(C([O-])=O)cc3)=O)cc(S(=O)(Nc2cccc([C@](F)(F)F)c2)=O)c1C	CHEMBL3431937	-6.08
243	CHEMBL3431521	c1cc(NC(c2ccc(Cl)c(NS(=O)(c3cc(Cl)cc(Cl)c3)=O)c2)=O)ccc1C([O-])=O	CHEMBL3431937	-5.65
244	CHEMBL3431522	COc1ccc(Cl)cc1S(=O)(Nc2cc(C(Nc3ccc(C([O-])=O)cc3)=O)cc(OC)c2OC)=O	CHEMBL3431937	-5.80
245	CHEMBL3431523	c1ccc(OCC(N2CCCC[C@@]2c3nc(c4ccc(Cl)cc4)no3)=O)cc1	CHEMBL3431937	-6.02
246	CHEMBL3431524	c1ccc(OCC(N2CCCC[C@@]2c3nc(c4cccc(C(N)=O)c4)no3)=O)cc1	CHEMBL3431937	-5.59
247	CHEMBL3431525	c1ccc(OCC(N2CCCC[C@@]2c3nc(c4ccc(S(=O)(NCCO)=O)cc4)no3)=O)cc1	CHEMBL3431937	-5.64
248	CHEMBL3431526	c1ccc(OCC(N2CCCC[C@@]2c3nc(c4ccnc(C(N5CCOCC5)=O)c4)no3)=O)cc1	CHEMBL3431937	-5.23
249	CHEMBL3431527	c1ccc(OCC(N2CCCC[C@@]2c3nc(c4ccc(c5cncn5)cc4)no3)=O)cc1	CHEMBL3431937	-6.58
250	CHEMBL3431528	c1ccc(OCC(N2CCCC[C@@]2c3nnc(c4cccc(N)c4)n3)=O)cc1	CHEMBL3431937	-5.27
251	CHEMBL3431529	CC(Nc1cccc(c2nc([C@]3CCCCN3C(COc4ccccc4)=O)nn2)c1)=O	CHEMBL3431937	-5.54
252	CHEMBL3431530	CS(=O)(Nc1cccc(c2nc([C@]3CCCCN3C(COc4ccccc4)=O)nn2)c1)=O	CHEMBL3431937	-5.59
253	CHEMBL3431531	c1ccc(OCC(N2CCCC[C@@]2c3nnc(c4cccc(C(N)=O)c4)n3)=O)cc1	CHEMBL3431937	-5.65
254	CHEMBL3431532	c1cc(O[C@@](F)F)ccc1Cn3c2ccc(F)cc2c(NC(c4ccc(C([O-])=O)cc4)=O)n3	CHEMBL3431937	-5.50
255	CHEMBL3431535	COc1ccc(Cl)cc1S(=O)(N2CCCc3c2cc(C(Nc4ccc(C([O-])=O)cc4)=O)cc3)=O	CHEMBL3431937	-5.50
256	CHEMBL3431536	COc1ccc(Cl)cc1S(=O)(N2CCOc3c2cc(C(Nc4ccc(C([O-])=O)cc4)=O)cc3)=O	CHEMBL3431937	-5.57
257	CHEMBL3431537	c1ccc(OCC(N2CCCC[C@@]2c3nc(c4ccc5c(NC(N5)=O)c4)no3)=O)cc1	CHEMBL3431937	-5.61
258	CHEMBL3431538	c1cc(O[C@@](F)F)ccc1Cn3c2ccc(F)cc2c(C(Nc4ccc(c5nnnn5)cc4)=O)n3	CHEMBL3431937	-6.34

259	CHEMBL3431539	C[N@@H+]1CCN(C(COc4ccccc4)=O)[C@@](c2nnc(c3cccc([C@](F)(F)F)c3)n2)C1	CHEMBL3431937	-5.64
260	CHEMBL3431540	c1ccc(OCC(N2CCCC[C@@]2c3nc(c4ccc5c(CC(N5)=O)c4)no3)=O)cc1	CHEMBL3431937	-5.58
261	CHEMBL3431541	COc1ccc(Cl)cc1S(=O)(Nc2cccc(C#Cc3ccc(C([O-])=O)cc3)c2)=O	CHEMBL3431937	-6.13
262	CHEMBL3431542	c1ccc(OCC(N2CCCC[C@@]2c3nc(c4ccc5c(ncn5)c4)no3)=O)cc1	CHEMBL3431937	-5.55
263	CHEMBL3431544	COc1ccc(Cl)cc1S(=O)(N2CCOc3c2cc(C(Nc4ccc(C5NC(ON5)=O)cc4)=O)cc3)=O	CHEMBL3431937	-6.03
264	CHEMBL3431545	c1ccc(OCC(N2CCCC[C@@]2c3nnc(c4ccc5c(NC(C5)=O)c4)n3)=O)cc1	CHEMBL3431937	-5.48
265	CHEMBL3431546	c1cc([C@](F)(F)F)cc(S(=O)(N2CCc3c2cc(C(Nc4ccc(C([O-])=O)c(F)c4)=O)cc3)=O)c1	CHEMBL3431937	-5.70
266	CHEMBL3431548	COc1ccc(Cl)cc1S(=O)(N2CCc3c2cc(C(Nc4ccc(C([O-])=O)c(F)c4)=O)cc3)=O	CHEMBL3431937	-5.78
267	CHEMBL3431549	COc1ccc(Cl)cc1S(=O)(N2CCc3c2cc(C(Nc4ccc(C([O-])=O)c(Cl)c4)=O)cc3)=O	CHEMBL3431937	-5.92
268	CHEMBL3431550	c1cc3c(N(S(=O)(c4cc(Cl)cc(Cl)c4)=O)CC3)cc1C(Nc2ccc(C([O-])=O)c(Cl)c2)=O	CHEMBL3431937	-5.72
269	CHEMBL3431551	Cc1ccc(OC)c(S(=O)(N2CCCc3c2cc(C(Nc4ccc(C([O-])=O)c(F)c4)=O)cc3)=O)c1	CHEMBL3431937	-5.79
270	CHEMBL3431552	COc1ccc(Cl)cc1S(=O)(N2CCCc3c2cc(C(Nc4ccc(C([O-])=O)c(F)c4)=O)cc3)=O	CHEMBL3431937	-5.75
271	CHEMBL3431553	c1cc3c(N(S(=O)(c4cc(Cl)cc(Cl)c4)=O)CCC3)cc1C(Nc2ccc(C([O-])=O)c(F)c2)=O	CHEMBL3431937	-5.32
272	CHEMBL3431554	c1cc([C@](F)(F)F)cc(S(=O)(N2CCCc3c2cc(C(Nc4ccc(C([O-])=O)c(F)c4)=O)cc3)=O)c1	CHEMBL3431937	-5.67
273	CHEMBL3431555	CN1C=C(C(Nc3ccc(C([O-])=O)cc3)=O)C=C(NS(=O)(c2cc(Cl)ccc2OC)=O)C1=O	CHEMBL3431937	-6.57
274	CHEMBL3431556	c1ccc(OCC(N2CC[NH2+]C[C@@]2c3nc(c4ccc5c(ncn5)c4)no3)=O)cc1	CHEMBL3431937	-5.82
275	CHEMBL3431557	C[N@@H+]1CCN(C(COc5ccccc5)=O)[C@@](c2nc(c3ccc4c(CC(N4)=O)c3)no2)C1	CHEMBL3431937	-5.47
276	CHEMBL3431558	c1ccc(OCC(N2CC[NH2+]C[C@@]2c3nc(c4ccc5c(CC(N5)=O)c4)no3)=O)cc1	CHEMBL3431937	-5.56
277	CHEMBL3431559	c1ccc(OCC(N2CCCC[C@@]2c3nc(C4C=CC(NC4)=O)no3)=O)cc1	CHEMBL3431937	-5.32
278	CHEMBL3431560	C[N@@H+]1CCN(C(COc5ccccc5)=O)[C@@](c2nc(c3ccc4c(nnn4)c3)no2)C1	CHEMBL3431937	-5.43
279	CHEMBL3431561	c1ccc(OCC(N2CCCC[C@@]2c3nc(c4ccc5c(nnc5C(N)=O)c4)no3)=O)cc1	CHEMBL3431937	-5.63
280	CHEMBL3431562	c1ccc(OCC(N2CCCC[C@@]2c3nc(c4cc(C([O-])=O)cnc4)no3)=O)cc1	CHEMBL3431937	-7.16
281	CHEMBL3431563	CCOC(c1cc(c2nc([C@]3CCCCN3C(COc4ccccc4)=O)on2)cnc1)=O	CHEMBL3431937	-5.81
282	CHEMBL3431564	c1cc(NC(c2ccc(NS(=O)(c3cc(Cl)cc(Cl)c3)=O)s2)=O)ccc1C([O-])=O	CHEMBL3431937	-8.00
283	CHEMBL3431565	c1ccc(OCC(N2CCCC[C@@]2c3nnc(c4ccc5c(cnn5)c4)n3)=O)cc1	CHEMBL3431937	-5.79
284	CHEMBL3431566	c1ccc(OCC(N2CCCC[C@@]2c3nc(c4cc(C(N)=O)cnc4)no3)=O)cc1	CHEMBL3431937	-5.42
285	CHEMBL3431567	CCOC(c2cn1ccc(c3nc([C@]4CCCCN4C(COc5ccccc5)=O)on3)cc1n2)=O	CHEMBL3431937	-5.70
286	CHEMBL3431568	c1ccc(OCC(N2CCCC[C@@]2c3nc(c5ccn4cc(C(N)=O)nc4c5)no3)=O)cc1	CHEMBL3431937	-5.26
287	CHEMBL3431569	c1ccc(OCC(N2CCCC[C@@]2c3nnc(c4cccc(C(NCCO)=O)c4)n3)=O)cc1	CHEMBL3431937	-6.29
288	CHEMBL3431570	COC[C@@]1CC[N@@H+](NC(c2cccc(c3nc([C@]4CCCCN4C(COc5ccccc5)=O)nn3)c2)=O)C1	CHEMBL3431937	-5.48
289	CHEMBL3431571	c1ccc(OCC(N2CCCC[C@@]2c3nnc(c4cccc(C#N)c4)n3)=O)cc1	CHEMBL3431937	-5.53
290	CHEMBL3431572	c1ccc(OCC(N2CCCC[C@@]2c3nc(c4ccnc(N)c4)no3)=O)cc1	CHEMBL3431937	-5.28
291	CHEMBL3431573	COc1ccc(Cl)cc1S(=O)(N2CCOc3c2cc(C(Nc4ccc(C([O-])=O)c(F)c4)=O)cc3)=O	CHEMBL3431937	-5.77
292	CHEMBL3431574	COc1ccc(Cl)cc1S(=O)(N2CCOc3c2cc(C(Nc4ccc(C([O-])=O)c(Cl)c4)=O)cc3)=O	CHEMBL3431937	-5.84
293	CHEMBL3431575	COc1ccc(Cl)cc1S(=O)(N2CCc3c2cc(C#Cc4ccc(C([O-])=O)cc4)cc3)=O	CHEMBL3431937	-6.59
294	CHEMBL3431576	COc1ccc(Cl)cc1S(=O)(N2CCNc3c2cc(C(Nc4ccc(C([O-])=O)cc4)=O)cc3)=O	CHEMBL3431937	-6.09
295	CHEMBL3431577	COc1ccc(Cl)cc1S(=O)(N2CCOc3c2cc(C(Nc4ccc(C([O-])=O)nc4)=O)cc3)=O	CHEMBL3431937	-6.24
296	CHEMBL3431578	CCC(Nc1cccc(c2nc([C@]3CCCCN3C(COc4ccccc4)=O)nn2)c1)=O	CHEMBL3431937	-5.54
297	CHEMBL3431579	C[C@@](C(Nc1cccc(c2nc([C@]3CCCCN3C(COc4ccccc4)=O)nn2)c1)=O)C	CHEMBL3431937	-5.59

298	CHEMBL3431580	Cc1cc(C(Nc3ccc(c4nnnn4)cc3)=O)nn1Cc2ccc(O[C@@](F)F)cc2	CHEMBL3431937	-5.91
299	CHEMBL3431581	CC(Nc1cccc(c2nc([C@]3CCCCN3C(COc4ccccc4)=O)no2)c1)=O	CHEMBL3431937	-5.59
300	CHEMBL3431582	CCNC(Nc1cccc(c2nc([C@]3CCCCN3C(COc4ccccc4)=O)nn2)c1)=O	CHEMBL3431937	-5.65
301	CHEMBL3431583	c1ccc(OCC(N2CCCC[C@@]2c3nnc(c4cccc(N5CCC5=O)c4)n3)=O)cc1	CHEMBL3431937	-5.70
302	CHEMBL3431584	c1ccc(c2cc([C@@]3CCCCN3C(COc4ccccc4)=O)nn2)cc1	CHEMBL3431937	-6.28
303	CHEMBL3431585	COc1ccc(Cl)cc1S(=O)(N2COc3c2cc(C(Nc4ccc(C([O-])=O)cc4)=O)cc3)=O	CHEMBL3431937	-5.53
304	CHEMBL3431586	COc1ccc(Cl)cc1S(=O)(N[C@@]2CCCN(C(COc3ccccc3)=O)C2)=O	CHEMBL3431937	-5.87
305	CHEMBL3431587	COc1ccc(Cl)cc1S(=O)(N2COc3c2cc(C(Nc4ccc(C([O-])=O)c(Cl)c4)=O)cc3)=O	CHEMBL3431937	-5.78
306	CHEMBL3431589	c1ccc(OCC(N2CCCC[C@]2c3ncc(c4cccc([N+]([O-])=O)c4)o3)=O)cc1	CHEMBL3431937	-6.18
307	CHEMBL3431591	c1ccc(OCC(N2CCCC[C@@]2c3nc(c4cccc(O)c4)no3)=O)cc1	CHEMBL3431937	-6.19
308	CHEMBL3431592	c1ccc(OCC(N2CCCC[C@@]2c3nc(C4C=CNC(C4)=O)no3)=O)cc1	CHEMBL3431937	-5.27
309	CHEMBL3431594	c1ccc(OCC(N2CCCC[C@@]2c3nnc(c4ccnc(N)c4)n3)=O)cc1	CHEMBL3431937	-5.56
310	CHEMBL3431595	COc1ccc(Cl)cc1S(=O)(N2COc3c2cc(C(Nc4ccc(C([O-])=O)c(F)c4)=O)cc3)=O	CHEMBL3431937	-5.72
311	CHEMBL3431596	COc1ccc(Cl)cc1S(=O)(N2CCCCc3c2cc(C(Nc4ccc(C([O-])=O)cc4)=O)cc3)=O	CHEMBL3431937	-5.54
312	CHEMBL3431597	c1ccc(OCC(N2CCCC[C@@]2c3nnc(c4cccc(C(N[NH3+])=O)c4)n3)=O)cc1	CHEMBL3431937	-5.70
313	CHEMBL3431598	c1ccc(OCC(N2CCCC[C@@]2c3nc(c4cccc([N+]([O-])=O)n4)no3)=O)cc1	CHEMBL3431937	-5.26
314	CHEMBL3431600	c1ccc(OCC(N2CCCC[C@@]2c3nnc(c4cccc(C5=NNC(O5)=O)c4)n3)=O)cc1	CHEMBL3431937	-5.77
315	CHEMBL3431601	c1ccc(OCC(N2CCCC[C@@]2c3nnc(c4cccc(c5nnco5)c4)n3)=O)cc1	CHEMBL3431937	-5.52
316	CHEMBL3431602	Cc1cc(C(Nc4ccc(C([O-])=O)c(F)c4)=O)cc2c1CCN2S(=O)(c3cc(Cl)ccc3OC)=O	CHEMBL3431937	-5.78
317	CHEMBL3431603	c1cc(S(=O)(N2CCOc3c2cc(C(Nc4ccc(C([O-])=O)c(F)c4)=O)cc3)=O)cc(Cl)c1	CHEMBL3431937	-5.66
318	CHEMBL3431605	Cc1cc(C(Nc4ccc(C([O-])=O)cc4)=O)cc2c1CCN2S(=O)(c3cc(Cl)ccc3OC)=O	CHEMBL3431937	-5.70
319	CHEMBL3431606	Cc1cc(C(Nc4ccc(C([O-])=O)c(Cl)c4)=O)cc2c1CCN2S(=O)(c3cc(Cl)ccc3OC)=O	CHEMBL3431937	-5.82
320	CHEMBL3431609	CN(C(c1ccc(c2nc([C@@]4[C@]3C[C@]3CN4C(COc5ccccc5)=O)on2)cc1)=O)C	CHEMBL3431937	-5.36
321	CHEMBL3431610	COc1ccc(Cl)cc1S(=O)(N2CCNc3c2cc(C(Nc4ccc(C([O-])=O)c(F)c4)=O)cc3)=O	CHEMBL3431937	-6.64
322	CHEMBL3431611	c1cc(S(=O)(N2CCOc3c2cc(C(Nc4ccc(C([O-])=O)cc4)=O)cc3)=O)cc(Cl)c1	CHEMBL3431937	-5.52
323	CHEMBL3431612	COc1ccc(Cl)cc1S(=O)(N2CCCCc3c2cc(C(Nc4ccc(C([O-])=O)c(F)c4)=O)cc3)=O	CHEMBL3431937	-5.67
324	CHEMBL3431613	COc1ccc(Cl)cc1S(=O)(N2CCCCc3c2cc(C(Nc4ccc(C([O-])=O)c(Cl)c4)=O)cc3)=O	CHEMBL3431937	-5.60
325	CHEMBL3431614	c1ccc(OCC(N2CCOC[C@@]2c3nc(c4cc(C(N)=O)cnc4)no3)=O)cc1	CHEMBL3431937	-5.54
326	CHEMBL3431615	c1ccc(OCC(N2CCCC[C@@]2c3nc(c4cc(C#N)cnc4)no3)=O)cc1	CHEMBL3431937	-5.20
327	CHEMBL3431616	CNC(c1cc(c2nc([C@]3CCCCN3C(COc4ccccc4)=O)on2)cnc1)=O	CHEMBL3431937	-5.42
328	CHEMBL3431617	CCN(C(c1cc(c2nc([C@]3CCCCN3C(COc4ccccc4)=O)on2)cnc1)=O)CC	CHEMBL3431937	-5.26
329	CHEMBL3431618	CN(C(c1cc(c2nc([C@]3CCCCN3C(COc4ccccc4)=O)on2)cnc1)=O)CCOC	CHEMBL3431937	-5.38
330	CHEMBL3431619	c1ccc(OCC(N2CCCC[C@@]2c3nc(c4cc(C(N[C@]5CC5)=O)cnc4)no3)=O)cc1	CHEMBL3431937	-5.50
331	CHEMBL3431620	c1ccc(OCC(N3C[C@@]2C[C@@]2[C@]3c4nc(c5ccc6c(CC(N6)=O)c5)no4)=O)cc1	CHEMBL3431937	-5.27
332	CHEMBL3431621	COc1ccc(Cl)cc1S(=O)(N2COc3c2cc(C(Nc4ccc(CC([O-])=O)cc4)=O)cc3F)=O	CHEMBL3431937	-5.55
333	CHEMBL3431622	COc1ccc(Cl)cc1S(=O)(N2COc3c2cc(C(Nc4nc(CC([O-])=O)cs4)=O)cc3F)=O	CHEMBL3431937	-5.65
334	CHEMBL3431623	COc1ccc(Cl)cc1S(=O)(N2CCSc3c2cc(C(Nc4ccc(CC([O-])=O)cc4)=O)cc3)=O	CHEMBL3431937	-5.29
335	CHEMBL3431624	COc1ccc(Cl)cc1S(=O)(N2CCSc3c2cc(C(Nc4nc(CC([O-])=O)cs4)=O)cc3)=O	CHEMBL3431937	-5.44
336	CHEMBL3431625	COc1ccc(Cl)cc1CN3c2cc(C(Nc4nc(CC([O-])=O)cs4)=O)ccc2OCC3=O	CHEMBL3431937	-5.95

337	CHEMBL3431626	COc1ccc(Cl)cc1S(=O)(N2CCc3c2cc(C(Nc4ccc(CC([O-])=O)cc4)=O)cc3)=O	CHEMBL3431937	-5.39
338	CHEMBL3431627	COc1ccc(Cl)cc1S(=O)(N2CCc3c2cc(C(Nc4nc(CC([O-])=O)cs4)=O)cc3)=O	CHEMBL3431937	-5.49
339	CHEMBL3431628	Cc1ccc(OC)c(S(=O)(n2ccc3c2cc(C(Nc4ccc(C([O-])=O)cc4)=O)cc3)=O)c1	CHEMBL3431937	-5.91
340	CHEMBL3431629	c1cc(S(=O)(n2ccc3c2cc(C(Nc4ccc(C([O-])=O)cc4)=O)cc3)=O)cc(Cl)c1	CHEMBL3431937	-6.22
341	CHEMBL3431630	Cc1cc(C)cc(S(=O)(n2ccc3c2cc(C(Nc4ccc(C([O-])=O)cc4)=O)cc3)=O)c1	CHEMBL3431937	-5.84
342	CHEMBL3431631	c1ccc(OCC(N2CCCC[C@@]2c3nc(c4ccc5c(NC(CO5)=O)c4)on3)=O)cc1	CHEMBL3431937	-5.43
343	CHEMBL3431632	C[C@@]2(C)c1ccc(C(Nc4nc(CC([O-])=O)cs4)=O)cc1N(Cc3cc(Cl)ccc3OC)C2=O	CHEMBL3431937	-6.02
344	CHEMBL3431633	C[C@@]2(C)c1ccc(C(Nc4ccc(C([O-])=O)cc4)=O)cc1N(Cc3cc(Cl)ccc3OC)C2=O	CHEMBL3431937	-6.03
345	CHEMBL3431635	COc1ccc(Cl)cc1S(=O)(N2CCOc3c2cc(C(Nc4ccc(CC([O-])=O)cc4)=O)cc3)=O	CHEMBL3431937	-5.73
346	CHEMBL3431636	CCN2c1cc(c3nc([C@]4CCCCN4C(COc5ccccc5)=O)no3)ccc1[C@@](C)(C)CC2=O	CHEMBL3431937	-6.40
347	CHEMBL3431637	c1ccc(OCC(N2CCCC[C@@]2c3nc([C@@]4CCCC4)on3)=O)cc1	CHEMBL3431937	-5.36
348	CHEMBL3431638	COc1ccc(Cl)cc1S(=O)(N2CCOc3c2cc(C(Nc4nc(CC([O-])=O)cs4)=O)cc3)=O	CHEMBL3431937	-5.79
349	CHEMBL3431639	c1cc(c2nc([C@]3CCCCN3C(COc4ccncc4)=O)nn2)cc([C@](F)(F)F)c1	CHEMBL3431937	-5.76
350	CHEMBL3431640	CC(Nc1cccc(c2nc([C@]3CCCCN3C(COc4ccccc4)=O)n(C)n2)c1)=O	CHEMBL3431937	-5.53
351	CHEMBL3431641	Cn1cc(Sc4cccc(Cl)c4)c2c1ccc(C(Nc3ccc(C([O-])=O)cc3)=O)c2	CHEMBL3431937	-6.89
352	CHEMBL3431642	Cn1cc(S(=O)(c4cccc(Cl)c4)=O)c2c1ccc(C(Nc3ccc(C([O-])=O)cc3)=O)c2	CHEMBL3431937	-6.00
353	CHEMBL3431643	c1ccc(OCC(N2CCCC[C@@]2c3nc(c5cc4c(nc5)ncn4)on3)=O)cc1	CHEMBL3431937	-5.45
354	CHEMBL3431644	CC(Nc1cccc(C#C[C@]2CCCCN2C(COc3ccccc3)=O)c1)=O	CHEMBL3431937	-5.75
355	CHEMBL3431645	CC(Nc1cc(c2nc([C@]3CCCCN3C(COc4ccccc4)=O)no2)ccn1)=O	CHEMBL3431937	-5.27
356	CHEMBL3431646	COc1ccc(Cl)cc1CN3c2cc(C(Nc4nc(CC([O-])=O)cs4)=O)ccc2SCC3=O	CHEMBL3431937	-5.82
357	CHEMBL3431647	COc1ccc(Cl)cc1S(=O)(N2COCc3c2cc(C(Nc4ccc(C([O-])=O)cc4)=O)cc3)=O	CHEMBL3431937	-5.67
358	CHEMBL3431648	COc1ccc(Cl)cc1S(=O)(N2COCc3c2cc(C(Nc4ccc(CC([O-])=O)cc4)=O)cc3)=O	CHEMBL3431937	-5.86
359	CHEMBL3431649	COc1ccc(Cl)cc1S(=O)(N2COCc3c2cc(C(Nc4nc(CC([O-])=O)cs4)=O)cc3)=O	CHEMBL3431937	-5.88
360	CHEMBL3431650	c1ccc(OCC(N2CCCC[C@@]2c3nc([C@@]4([C@](F)(F)F)CC4)on3)=O)cc1	CHEMBL3431937	-5.27
361	CHEMBL3431651	c1ccc(OCC(N2CCCC[C@@]2c3nc(c4cc(Cl)nnc4O)on3)=O)cc1	CHEMBL3431937	-5.21
362	CHEMBL3431652	C[C@@]2(C)c1ccc(C(Nc4nccs4)=O)cc1N(Cc3cc(Cl)ccc3OC)C(O2)=O	CHEMBL3431937	-6.52
363	CHEMBL3431653	c1ccc(OCC(N2CCCC[C@@]2c3nc(c4ccc5c(NC(C(N5)=O)=O)c4)on3)=O)cc1	CHEMBL3431937	-6.33
364	CHEMBL3431654	c1ccc(OCC(N2CCCC[C@@]2c3nc(c5ccc4nccn4c5)no3)=O)cc1	CHEMBL3431937	-5.20
365	CHEMBL3431655	c1ccc(OCC(N2CCCC[C@@]2c3nc(c4ccc5c(NC(CO5)=O)c4)no3)=O)cc1	CHEMBL3431937	-5.41
366	CHEMBL3431659	COc1ccc(Cl)cc1S(=O)(N2CCS(c3c2cc(C(Nc4ccc(C([O-])=O)cc4)=O)cc3)=O)=O	CHEMBL3431937	-6.92
367	CHEMBL3431660	c1ccc(OCC(N2CCCC[C@@]2c3nc(c4ccc5c(NC(CN5)=O)c4)on3)=O)cc1	CHEMBL3431937	-5.74
368	CHEMBL3431661	COc1ccc(Cl)cc1S(=O)(N2CCOc3c2cc(C(Nc4ccc(CCC([O-])=O)cc4)=O)cc3)=O	CHEMBL3431937	-5.48
369	CHEMBL3431662	COc1ccc(Cl)cc1CN3c2cc(C(Nc4nc(CC([O-])=O)cs4)=O)ccc2S(=O)(CC3=O)=O	CHEMBL3431937	-6.07
370	CHEMBL3431663	c1ccc(OCC(N2CCCC[C@@]2c3nnc(c4ccc5c(NC(CO5)=O)c4)n3)=O)cc1	CHEMBL3431937	-6.02
371	CHEMBL3431664	CC(Nc1ccc(F)c(c2nc([C@]3CCCCN3C(COc4ccccc4)=O)nn2)c1)=O	CHEMBL3431937	-5.37
372	CHEMBL3431665	c1ccc(OCC(N2CCCC[C@@]2c3nc(c4cccc(N)n4)on3)=O)cc1	CHEMBL3431937	-5.26
373	CHEMBL3431666	c1ccc(OCC(N2CCCC[C@@]2c3nc(c4ccc(C#N)nc4)on3)=O)cc1	CHEMBL3431937	-5.07
374	CHEMBL3431667	c1ccc(OCC(N2CCCC[C@@]2c3nc(c4cccc5c4C(NN5)=O)on3)=O)cc1	CHEMBL3431937	-5.19
375	CHEMBL3431668	C[C@@]2(C)c1ccc(C(Nc4ccc(C([O-])=O)c(Cl)c4)=O)cc1N(Cc3cc(Cl)ccc3OC)C(O2)=O	CHEMBL3431937	-5.96

376	CHEMBL3431669	C[C@@]2(C)c1ccc(C(Nc4ccc(CC([O-])=O)c(Cl)c4)=O)cc1N(Cc3cc(Cl)ccc3OC)C(O2)=O	CHEMBL3431937	-5.87
377	CHEMBL3431670	c1ccc(OCC(N2CCCC[C@@]2c3nc(c4ccnc(N)c4)on3)=O)cc1	CHEMBL3431937	-5.31
378	CHEMBL3431671	C[C@@]2(C)c1ccc(C(Nc4ccc(CC([O-])=O)cc4)=O)cc1N(Cc3cc(Cl)ccc3OC)C(O2)=O	CHEMBL3431937	-5.77
379	CHEMBL3431672	CC(Nc1cc(c2nc([C@]3CCCCN3C(COc4ccccc4)=O)nn2)cc(F)c1)=O	CHEMBL3431937	-5.77
380	CHEMBL3431673	CC(Nc1cc(c2nc([C@]3CCCCN3C(COc4ccccc4)=O)nn2)ccc1F)=O	CHEMBL3431937	-5.57
381	CHEMBL3431674	CC(Nc1cc(c2nc([C@]3CCCCN3C(COc4ccccc4)=O)on2)cc(F)c1)=O	CHEMBL3431937	-5.87
382	CHEMBL3431675	CC(Nc1cc(c2nc([C@]3CCCCN3C(COc4ccccc4)=O)on2)ccc1F)=O	CHEMBL3431937	-5.66
383	CHEMBL3431677	Cc1cccc(S(=O)(N2CCOc3c2cc(C(Nc4nc(CC([O-])=O)cs4)=O)cc3)=O)c1	CHEMBL3431937	-5.51
384	CHEMBL3431678	c1cc(S(=O)(N2CCOc3c2cc(C(Nc4nc(CC([O-])=O)cs4)=O)cc3)=O)cc(Cl)c1	CHEMBL3431937	-5.54
385	CHEMBL3431679	Cc1cc(C)cc(S(=O)(N2CCOc3c2cc(C(Nc4nc(CC([O-])=O)cs4)=O)cc3)=O)c1	CHEMBL3431937	-5.50
386	CHEMBL3431681	COc1ccc(Cl)cc1S(=O)(n2ccc3c2cc(C(Nc4ccc(CC([O-])=O)cc4)=O)cc3)=O	CHEMBL3431937	-5.70
387	CHEMBL3431682	Cc1ccc(OC)c(S(=O)(n2ccc3c2cc(C(Nc4ccc(CC([O-])=O)cc4)=O)cc3)=O)c1	CHEMBL3431937	-5.55
388	CHEMBL3431683	c1ccc(OCC(N2CCCC[C@@]2c3nnc(c4ccc(N)nc4)n3)=O)cc1	CHEMBL3431937	-5.40
389	CHEMBL3431685	Cn1cc(S(=O)(c4cccc(Cl)c4)=O)c2c1ccc(C(Nc3nc(CC([O-])=O)cs3)=O)c2	CHEMBL3431937	-6.02
390	CHEMBL3431686	Cc1cc(C)cc(S(=O)(n2ccc3c2cc(C(Nc4ccc(CC([O-])=O)cc4)=O)cc3)=O)c1	CHEMBL3431937	-5.72
391	CHEMBL3431687	c1cc(S(=O)(n2ccc3c2cc(C(Nc4ccc(CC([O-])=O)cc4)=O)cc3)=O)cc(Cl)c1	CHEMBL3431937	-5.65
392	CHEMBL3431688	CC(NCc1cccc(c2nc([C@]3CCCCN3C(COc4ccccc4)=O)nn2)c1)=O	CHEMBL3431937	-5.39
393	CHEMBL3431689	Cc1ccc(OC)c(S(=O)(N2CCc3c2cc(C(Nc4ccc(CC([O-])=O)cc4)=O)cc3)=O)c1	CHEMBL3431937	-5.66
394	CHEMBL3431690	Cc1cc(C)cc(S(=O)(N2CCc3c2cc(C(Nc4ccc(CC([O-])=O)cc4)=O)cc3)=O)c1	CHEMBL3431937	-5.39
395	CHEMBL3431691	c1cc(S(=O)(N2CCc3c2cc(C(Nc4ccc(CC([O-])=O)cc4)=O)cc3)=O)cc(Cl)c1	CHEMBL3431937	-5.44
396	CHEMBL3431692	c1ccc(OCC(N2CCCC[C@@]2C(N4CCc3ccccc3C4)=O)=O)cc1	CHEMBL3431937	-5.38
397	CHEMBL3431693	c1ccc(CNC([C@]2CCCCN2C(COc3ccccc3)=O)=O)cc1	CHEMBL3431937	-5.15
398	CHEMBL3431694	CN(C([C@]2CCCCN2C(COc3ccccc3)=O)=O)Cc1ccccc1	CHEMBL3431937	-5.04
399	CHEMBL3431695	CCN(C(c1nc([C@]2CCCCN2C(COc3ccccc3)=O)no1)=O)CC	CHEMBL3431937	-4.94
400	CHEMBL3431697	c1ccc(OCC(N2CCCC[C@@]2c3nc(c4ccc([N+]([O-])=O)s4)no3)=O)cc1	CHEMBL3431937	-5.36
401	CHEMBL3431698	c1ccc(OCC(N2CCCC[C@@]2c3nc(c4ccc([N+]([O-])=O)o4)no3)=O)cc1	CHEMBL3431937	-5.29
402	CHEMBL3431699	c1ccc(OCC(N2CCCC[C@@]2c3nnc(c4cccs4)n3)=O)cc1	CHEMBL3431937	-5.57
403	CHEMBL3431700	c1ccc(OCC(N2CCCC[C@@]2c3nnc(c4ccccc4F)n3)=O)cc1	CHEMBL3431937	-5.32
404	CHEMBL3431701	c1ccc(OCC(N2CCCC[C@@]2c3nnc(c4cccc(F)c4)n3)=O)cc1	CHEMBL3431937	-5.84
405	CHEMBL3431702	c1ccc(OCC(N2CCCC[C@@]2c3nnc(c4ccc(F)c(F)c4)n3)=O)cc1	CHEMBL3431937	-5.77
406	CHEMBL3431703	COc1ccc(c2nc([C@]3CCCCN3C(C#Cc4ccccc4)=O)on2)cc1	CHEMBL3431937	-5.61
407	CHEMBL3431704	COc1ccc(Cl)cc1S(=O)(N2C[C@](C([O-])=O)Oc3c2cc(C(Nc4ccccc4)=O)cc3)=O	CHEMBL3431937	-7.52
408	CHEMBL3431705	c1ccc(OCC(N2CCCC[C@@]2C#Cc3ccc(C#N)cc3)=O)cc1	CHEMBL3431937	-6.22
409	CHEMBL3431706	c1ccc(OCC(N2CCCC[C@@]2C#Cc3ccc(S(=O)(N)=O)cc3)=O)cc1	CHEMBL3431937	-5.39
410	CHEMBL3431707	c1ccc(OCC(N2CCCC[C@@]2C#Cc3ccc4c(OCC(N4)=O)c3)=O)cc1	CHEMBL3431937	-5.96
411	CHEMBL3431708	c1ccc(OCC(N2CCCC[C@@]2c3nnn(c4ccc5c(ccn5)c4)n3)=O)cc1	CHEMBL3431937	-5.64
412	CHEMBL3431709	CC(Nc1ccc(n2nc([C@]3CCCCN3C(COc4ccccc4)=O)nn2)cc1)=O	CHEMBL3431937	-5.57
413	CHEMBL3431710	c1ccc(OCC(N2CCCC[C@@]2C#Cc3ccc(C(N)=O)cc3)=O)cc1	CHEMBL3431937	-5.53
414	CHEMBL3431711	Cn1cc(Cc4cc(Cl)ccc4OC)c2c1ccc(C(Nc3nc(CC([O-])=O)cs3)=O)c2	CHEMBL3431937	-6.29

415	CHEMBL3431712	Cn1cc(Cc4cc(Cl)ccc4OC)c2c1ccc(C(Nc3ccc(CC([O-])=O)cc3)=O)c2	CHEMBL3431937	-6.07
416	CHEMBL3431713	c1ccc(OCC(N2CCCC[C@@]2c3nc(c4cccc(N5C=NNC5=O)c4)on3)=O)cc1	CHEMBL3431937	-5.46
417	CHEMBL3431714	c1ccc(OCC(N2CCCC[C@@]2c3nc(c4cccc(N5CC(NC5=O)=O)c4)on3)=O)cc1	CHEMBL3431937	-5.47
418	CHEMBL3431715	c1ccc(OCC(N2CCCC[C@@]2c3nc(c4cccc(N5CCCS5(=O)=O)c4)on3)=O)cc1	CHEMBL3431937	-6.00
419	CHEMBL3431716	c1ccc(OCC(N2CCCC[C@@]2c3nc(c4cccc(N5CCCC5=O)c4)on3)=O)cc1	CHEMBL3431937	-5.63
420	CHEMBL3431717	Cc1cc(C(Nc4ccc(CC([O-])=O)cc4)=O)cc2c1OCCN2S(=O)(c3cc(Cl)ccc3OC)=O	CHEMBL3431937	-5.68
421	CHEMBL3431718	Cc1cc(C(Nc4ccc(CC([O-])=O)cc4)=O)cc2c1OCN2S(=O)(c3cc(Cl)ccc3OC)=O	CHEMBL3431937	-5.50
422	CHEMBL3431719	Cc1cc(C(Nc4nc(CC([O-])=O)cs4)=O)cc2c1OCCN2S(=O)(c3cc(Cl)ccc3OC)=O	CHEMBL3431937	-5.73
423	CHEMBL3431720	Cn1cc(Cc4cc(Cl)ccc4OC)c2c1ccc(C(Nc3ccc(C([O-])=O)cc3)=O)c2	CHEMBL3431937	-6.33
424	CHEMBL3431721	Cc1cc(C(Nc4nc(CC([O-])=O)cs4)=O)cc2c1OCN2S(=O)(c3cc(Cl)ccc3OC)=O	CHEMBL3431937	-5.56
425	CHEMBL3431722	COc1ccc(Cl)cc1S(=O)(N2CCCc3c2cc(Oc4ccc(CC([O-])=O)cc4)cc3)=O	CHEMBL3431937	-6.11
426	CHEMBL3431724	CCOc1ccc(C(Nc3ccc(CC([O-])=O)cc3)=O)cc1N(S(=O)(c2cc(Cl)ccc2OC)=O)CCS([O-])=O	CHEMBL3431937	-7.40
427	CHEMBL3431725	CCOc1ccc(C(Nc3ccc(C([O-])=O)c(F)c3)=O)cc1N(S(=O)(c2cc(Cl)ccc2OC)=O)CCS([O-])=O	CHEMBL3431937	-7.16
428	CHEMBL3431726	COc1ccc(Cl)cc1CN3c2cc(C(Nc4ccc(CC([O-])=O)cc4)=O)ccc2COC3=O	CHEMBL3431937	-5.83
429	CHEMBL3431727	COc1ccc(Cl)cc1Cc2cnc3c2cc(C(Nc4nc(CC([O-])=O)cs4)=O)cc3	CHEMBL3431937	-6.31
430	CHEMBL3431728	COc1ccc(Cl)cc1Cc2cnc3c2cc(C(Nc4ccc(CC([O-])=O)cc4)=O)cc3	CHEMBL3431937	-6.37
431	CHEMBL3431729	c1ccc(OCC(N2CCCC[C@@]2C(N4CCc3cccc(Cl)c3C4)=O)=O)cc1	CHEMBL3431937	-5.72
432	CHEMBL3431731	COc1cccc2c1CCN(C([C@]3CCCCN3C(COc4ccccc4)=O)=O)C2	CHEMBL3431937	-5.26
433	CHEMBL3431732	c1ccc(OCC(N2CCCC[C@@]2C(N4CCc3ccc(S(=O)(N)=O)cc3C4)=O)=O)cc1	CHEMBL3431937	-5.35
434	CHEMBL3431734	C[N@@H+]1CCN(c2cc3c(CN(C([C@]4CCCCN4C(COc5ccccc5)=O)=O)C3)cc2Cl)CC1	CHEMBL3431937	-5.50
435	CHEMBL3431735	c1ccc(OCC(N2CCCC[C@@]2C(N4Cc3cc(N5CCOCC5)c(Cl)cc3C4)=O)=O)cc1	CHEMBL3431937	-5.23
436	CHEMBL3431736	CCOc1ccc2c(CN(C([C@]3CCCCN3C(COc4ccccc4)=O)=O)C2)c1	CHEMBL3431937	-5.26
437	CHEMBL3431737	c1ccc(OCC(N2CCCC[C@@]2C(N4Cc3ccc([C@@]5CCOCC5)cc3C4)=O)=O)cc1	CHEMBL3431937	-5.35
438	CHEMBL3431738	c1ccc(OCC(N2CCCC[C@@]2C(N4Cc3ccc(OC[C@]5CC5)cc3C4)=O)=O)cc1	CHEMBL3431937	-5.57
439	CHEMBL3431739	c1ccc(OCC(N2CCCC[C@@]2C(N4Cc3ccc([C@@]5CCOC5)cc3C4)=O)=O)cc1	CHEMBL3431937	-5.33
440	CHEMBL3431740	COc1ccc(Cl)cc1CN3c2cc(C(Nc4nc(CC([O-])=O)cs4)=O)ccc2COC3=O	CHEMBL3431937	-5.77
441	CHEMBL3431741	c1ccc(OCC(N2CCCC[C@@]2c3nc(c4cccc(N5C=CNC5=O)c4)on3)=O)cc1	CHEMBL3431937	-5.13
442	CHEMBL3431742	c1ccc(OCC(N2CCCC[C@@]2c3nc(C(N4CCOCC4)=O)on3)=O)cc1	CHEMBL3431937	-5.09
443	CHEMBL3431743	c1ccc(OCC(N2CCCC[C@@]2c3nc(C(N4CC[C@@](c5ccncc5)C4)=O)on3)=O)cc1	CHEMBL3431937	-5.37
444	CHEMBL3431744	Cn1ccc(c2nc([C@]3CCCCN3C(COc4ccccc4)=O)no2)n1	CHEMBL3431937	-5.03
445	CHEMBL3431745	c1ccc(OCC(N2CCCC[C@@]2c3nc(c4ccnn4)on3)=O)cc1	CHEMBL3431937	-5.23
446	CHEMBL3431746	Cc1cc(c2nc([C@]3CCCCN3C(COc4ccccc4)=O)no2)no1	CHEMBL3431937	-5.17
447	CHEMBL3431747	Cc1cc(c2nc([C@]3CCCCN3C(COc4ccccc4)=O)no2)n(C)n1	CHEMBL3431937	-5.19
448	CHEMBL3431748	Cc1cc(c2nc([C@]3CCCCN3C(COc4ccccc4)=O)no2)nn1	CHEMBL3431937	-5.22
449	CHEMBL3431749	Cc1cc(c2nc([C@]3CCCCN3C(COc4ccccc4)=O)no2)on1	CHEMBL3431937	-5.02
450	CHEMBL3431750	c1ccc(OCC(N2CCCC[C@@]2c3nc(c4cc([N+]([O-])=O)nn4)on3)=O)cc1	CHEMBL3431937	-5.16
451	CHEMBL3431751	c1ccc(OCC(N2CCCC[C@@]2c3nc(c4ccc5c(COC(N5)=O)c4)no3)=O)cc1	CHEMBL3431937	-5.45
452	CHEMBL3431752	Cc1cc(C)cc(S(=O)(N2CCc3c2cc(C(Nc4nc(CC([O-])=O)cs4)=O)cc3)=O)c1	CHEMBL3431937	-5.65
453	CHEMBL3431753	c1cc(S(=O)(N2CCc3c2cc(C(Nc4nc(CC([O-])=O)cs4)=O)cc3)=O)cc(Cl)c1	CHEMBL3431937	-5.54

454	CHEMBL3431754	COc1ccc(Cl)cc1S(=O)(N2CCOc3c2cc(C(Nc4ncc(CC([O-])=O)s4)=O)cc3)=O	CHEMBL3431937	-5.66
455	CHEMBL3431756	c1cc(CN3c2cc(C(Nc4ccc(C([O-])=O)cc4)=O)ccc2S(=O)(CC3=O)=O)cc(Cl)c1	CHEMBL3431937	-5.98
456	CHEMBL3431757	c1ccc(OCC(N2CCCC[C@@]2c3nnc(c4ccc5c(NC(NC5)=O)c4)n3)=O)cc1	CHEMBL3431937	-5.66
457	CHEMBL3431758	c1ccc(OCC(N2CCCC[C@@]2c3nc(c4cccc(N5CCCS5(=O)=O)c4)no3)=O)cc1	CHEMBL3431937	-4.61
458	CHEMBL3431759	c1ccc(OCC(N2CCCC[C@@]2c3nc(c4cccc(N5CCCC5=O)c4)no3)=O)cc1	CHEMBL3431937	-5.41
459	CHEMBL3431760	c1ccc(OCC(N2CCCC[C@@]2c3nc(c4cccc(N5CC(NC5=O)=O)c4)no3)=O)cc1	CHEMBL3431937	-5.42
460	CHEMBL3431761	c1ccc(OCC(N2CCCC[C@@]2c3nc(c4cccc(N5C=NNC5=O)c4)no3)=O)cc1	CHEMBL3431937	-5.31
461	CHEMBL3431762	c1ccc(OCc3nc2cc(F)ccc2n3[C@@]4CCN(C(COc5ccccc5)=O)CC4)cc1	CHEMBL3431937	-5.64
462	CHEMBL3431763	COc1ccc(Cl)cc1S(=O)(N2CCS(=O)(c3c2cc(C(Nc4ccc(C([O-])=O)c(F)c4)=O)cc3)=O)=O	CHEMBL3431937	-6.68
463	CHEMBL3431764	COc1ccc(Cl)cc1S(=O)(N2CCSc3c2cc(C(Nc4ccc(C([O-])=O)c(F)c4)=O)cc3)=O	CHEMBL3431937	-5.59
464	CHEMBL3431766	c1ccc(c2cn(CC(Nc4ccc(C([O-])=O)cc4)=O)c3c2cccc3)cc1	CHEMBL3431937	-5.76
465	CHEMBL3431767	COc1ccc(Cl)cc1S(=O)(N2CCS(=O)(c3c2cc(C(Nc4ccc(C([O-])=O)c(Cl)c4)=O)cc3)=O)=O	CHEMBL3431937	-7.22
466	CHEMBL3431768	CC(Nc1ccc(c2nc([C@]3CCCCN3C(C#Cc4ccccc4)=O)on2)cc1[C@](F)(F)F)=O	CHEMBL3431937	-5.82
467	CHEMBL3431769	CN(C(CCC(Nc1cc(c2nc([C@]3CCCCN3C(COc4ccccc4)=O)on2)cc(F)c1)=O)=O)C	CHEMBL3431937	-6.08
468	CHEMBL3431770	COc1ccc(Cl)cc1Cn2cc(C([O-])=O)c3c2cc(C(Nc4ccccc4)=O)cc3	CHEMBL3431937	-6.28
469	CHEMBL3431773	CN(S(=O)(c3cc(Cl)ccc3OC)=O)c1cccc(C(Nc2nc(CC([O-])=O)cs2)=O)c1	CHEMBL3431937	-5.61
470	CHEMBL3431779	COc1ccc(Cl)cc1Cn2ccc3c2cc(C(Nc4nc(CC([O-])=O)cs4)=O)cc3	CHEMBL3431937	-5.94
471	CHEMBL3431781	CCOC([C@]2CN(S(=O)(c4cc(Cl)ccc4OC)=O)c1cc(C(Nc3cccc(F)c3)=O)ccc1O2)=O	CHEMBL3431937	-5.88
472	CHEMBL3431782	Cn2c1ccc(C(Nc4ccc(C([O-])=O)cc4)=O)cc1c(S(=O)(c3cc(Cl)ccc3OC)=O)n2	CHEMBL3431937	-5.72
473	CHEMBL3431783	Cn2c(S(=O)(c4cc(Cl)ccc4OC)=O)c1cc(C(Nc3ccc(C([O-])=O)cc3)=O)ccc1n2	CHEMBL3431937	-5.65
474	CHEMBL3431784	COc1ccc(Cl)cc1S(=O)(N2C[C@](C([O-])=O)Oc3c2cc(C(Nc4ccccc4F)=O)cc3)=O	CHEMBL3431937	-6.42
475	CHEMBL3431785	COc1ccc(Cl)cc1S(=O)(N2C[C@](C([O-])=O)Oc3c2cc(C(Nc4ccc(F)cc4)=O)cc3)=O	CHEMBL3431937	-7.22
476	CHEMBL3431788	c1cc(S(=O)(N2CCS(=O)(c3c2cc(C(Nc4ccc(C([O-])=O)cc4)=O)cc3)=O)=O)cc(Cl)c1	CHEMBL3431937	-6.07
477	CHEMBL3431789	Cc1cc(C)cc(S(=O)(N2CCS(=O)(c3c2cc(C(Nc4ccc(C([O-])=O)cc4)=O)cc3)=O)=O)c1	CHEMBL3431937	-5.71
478	CHEMBL3431790	c1ccc(OCC(N2CCCC[C@@]2c3nnc(c4cccc(N5CC(NC5=O)=O)c4)n3)=O)cc1	CHEMBL3431937	-5.72
479	CHEMBL3431792	Cn2c1ccc(C(Nc4ccc(CC([O-])=O)cc4)=O)cc1c(S(=O)(c3cc(Cl)ccc3OC)=O)n2	CHEMBL3431937	-6.41
480	CHEMBL3431793	Cc1cc(C)cc(S(=O)(N2CCSc3c2cc(C(Nc4ccc(C([O-])=O)cc4)=O)cc3)=O)c1	CHEMBL3431937	-5.75
481	CHEMBL3431794	c1cc(S(=O)(N2CCSc3c2cc(C(Nc4ccc(C([O-])=O)cc4)=O)cc3)=O)cc(Cl)c1	CHEMBL3431937	-5.55
482	CHEMBL3431795	c1cc(S(=O)(N2CCS(c3c2cc(C(Nc4ccc(C([O-])=O)cc4)=O)cc3)=O)=O)cc(Cl)c1	CHEMBL3431937	-6.55
483	CHEMBL3431796	Cc1cc(C)cc(S(=O)(N2CCS(c3c2cc(C(Nc4ccc(C([O-])=O)cc4)=O)cc3)=O)=O)c1	CHEMBL3431937	-6.31
484	CHEMBL3431797	COc1ccc(Cl)cc1CN3c2cc(C(Nc5nc(CC([O-])=O)cs5)=O)ccc2[C@]4(CC4)C3=O	CHEMBL3431937	-5.99
485	CHEMBL3431798	COc1ccc(Cl)cc1S(=O)(Nc2cc(C(Nc3ccccc3)=O)ccc2CCC([O-])=O)=O	CHEMBL3431937	-5.62
486	CHEMBL3431799	c1ccc(OCC(N2CCCC[C@@]2C(N4Cc3cc(O[C@]5CCOCC5)c(F)cc3C4)=O)=O)cc1	CHEMBL3431937	-5.45
487	CHEMBL3431800	c1ccc(OCC(N2CCCC[C@@]2C(N4Cc3cc(N5CCOCC5)c(F)cc3C4)=O)=O)cc1	CHEMBL3431937	-5.08
488	CHEMBL3431802	c1ccc(OCC(N2CCCC[C@@]2C(N4Cc3ccc(O[C@@]5CCOC5)cc3C4)=O)=O)cc1	CHEMBL3431937	-4.94
489	CHEMBL3431804	c1ccc(c2csc([C@]3CCCCN3C(COc4ccccc4)=O)n2)cc1	CHEMBL3431937	-5.83
490	CHEMBL3431805	c1ccc(OCC(N2CCCC[C@@]2c3nc(c4ccc(C([O-])=O)cc4)cs3)=O)cc1	CHEMBL3431937	-5.29
491	CHEMBL3431806	c1ccc(OCC(N2CCCC[C@@]2c3nc(c4cccc(C([O-])=O)c4)cs3)=O)cc1	CHEMBL3431937	-5.30
492	CHEMBL3431807	c1ccc(OCC(N2CCCC[C@@]2c3nc(c4cccc(C([O-])=O)c4)cs3)=O)cc1	CHEMBL3431937	-5.45

493	CHEMBL3431808	c1ccc(OCC(N2CCCC[C@@]2c3nc(c4cccnc4)cs3)=O)cc1	CHEMBL3431937	-5.42
494	CHEMBL3431810	c1ccc(OCC(N2CCCC[C@@]2c3nc(c4ccncc4)cs3)=O)cc1	CHEMBL3431937	-5.42
495	CHEMBL3431811	COc1ccc(Cl)cc1CN3c2cc(C(Nc5ccc(C([O-])=O)cc5)=O)ccc2[C@]4(CC4)C3=O	CHEMBL3431937	-5.91
496	CHEMBL3431812	COc1ccc(Cl)cc1S(=O)(c2cnc3c2cc(C(Nc4ccccc4)=O)cc3)=O	CHEMBL3431937	-7.22
497	CHEMBL3431814	COc1ccc(Cl)cc1S(=O)(c2cn(CC(N)=O)c3c2cc(C(Nc4ccccc4)=O)cc3)=O	CHEMBL3431937	-6.52
498	CHEMBL3431816	COc1ccc(Cl)cc1S(=O)(c2cn(CCC([O-])=O)c3c2cc(C(Nc4ccccc4)=O)cc3)=O	CHEMBL3431937	-6.41
499	CHEMBL3431818	C[C@@](C(N(c2cc(C(Nc3nc(CC([O-])=O)cs3)=O)ccc2Cl)Cc1cc(Cl)ccc1OC)=O)C	CHEMBL3431937	-5.50
500	CHEMBL3431819	Cc1cc(C(Nc4ccc(CC([O-])=O)cc4)=O)cc2c1CCN2S(=O)(c3cc(Cl)ccc3OC)=O	CHEMBL3431937	-5.51
501	CHEMBL3431820	Cc1cc(C(Nc4nc(CC([O-])=O)cs4)=O)cc2c1CCN2S(=O)(c3cc(Cl)ccc3OC)=O	CHEMBL3431937	-5.69
502	CHEMBL3431821	COc1ccc(Cl)cc1S(=O)(N2CCCCc3c2cc(C(Nc4nc(CC([O-])=O)cs4)=O)cc3)=O	CHEMBL3431937	-5.46
503	CHEMBL3431822	Cc1ccc(OC)c(S(=O)(N2CCCc3c2cc(C(Nc4nc(CC([O-])=O)cs4)=O)cc3)=O)c1	CHEMBL3431937	-5.77
504	CHEMBL3431823	COc1ccc(Cl)cc1S(=O)(Nc2cc(C(Nc3ccccc3)=O)ccc2\C=C\C([O-])=O)=O	CHEMBL3431937	-6.00
505	CHEMBL3431824	COc1ccc(Cl)cc1S(=O)(N2COc3c2cc(C(Nc4ccn(CC([O-])=O)n4)=O)cc3)=O	CHEMBL3431937	-7.52
506	CHEMBL3431825	CN(S(=O)(c4cc(Cl)ccc4OC)=O)c1cc(C(Nc3nc(CC([O-])=O)cs3)=O)cc2c1OCO2	CHEMBL3431937	-5.61
507	CHEMBL3431826	CN(S(=O)(c4cc(Cl)ccc4OC)=O)c1cc(C(Nc3ccc(C([O-])=O)cc3)=O)cc2c1OCCO2	CHEMBL3431937	-5.60
508	CHEMBL3431827	CN(S(=O)(c4cc(Cl)ccc4OC)=O)c1cc(C(Nc3nc(CC([O-])=O)cs3)=O)cc2c1OCCO2	CHEMBL3431937	-5.89
509	CHEMBL3431829	CN(S(=O)(c4cc(Cl)ccc4OC)=O)c1cc(C(Nc3ccc(C([O-])=O)cc3)=O)cc2c1OCO2	CHEMBL3431937	-5.69
510	CHEMBL3431830	COc1ccc(Cl)cc1S(=O)(c2cn(CCCC([O-])=O)c3c2cc(C(Nc4ccccc4)=O)cc3)=O	CHEMBL3431937	-6.57
511	CHEMBL3431831	COc1ccc(Cl)cc1S(=O)(c2cn(CCO)c3c2cc(C(Nc4ccccc4)=O)cc3)=O	CHEMBL3431937	-7.22
512	CHEMBL3431832	COc1ccc(Cl)cc1S(=O)(c2cn(CCCO)c3c2cc(C(Nc4ccccc4)=O)cc3)=O	CHEMBL3431937	-7.22
513	CHEMBL3431835	COc1ccc(Cl)cc1S(=O)(N2CCc3c2cc(C(Nc4ccn(CC([O-])=O)n4)=O)cc3)=O	CHEMBL3431937	-6.64
514	CHEMBL3431837	COc1ccc(Cl)cc1S(=O)(c3c2cc(C(Nc4ccccc4)=O)ccc2n(CCC([O-])=O)n3)=O	CHEMBL3431937	-6.17
515	CHEMBL3431838	COc1ccc(Cl)cc1S(=O)(N2CCOc3c2cc(C(Nc4ccn(CC([O-])=O)n4)=O)cc3)=O	CHEMBL3431937	-7.05
516	CHEMBL3431841	Cc1cc(C)cc(S(=O)(c3c2cc(C(Nc4ccccc4)=O)ccc2n(CC([O-])=O)n3)=O)c1	CHEMBL3431937	-8.00
517	CHEMBL3431842	Cc1cc(C)cc(S(=O)(c3c2cc(C(Nc4ccccc4)=O)ccc2n(CCC([O-])=O)n3)=O)c1	CHEMBL3431937	-6.04
518	CHEMBL3431843	Cc1cc(C)cc(S(=O)(c3c2cc(C(Nc4ccc(C([O-])=O)cc4)=O)ccc2nn3)=O)c1	CHEMBL3431937	-7.52
519	CHEMBL3431844	Cc1cc(C)cc(S(=O)(c3c2cc(C(Nc4ccc(C([O-])=O)cc4)=O)ccc2n(C)n3)=O)c1	CHEMBL3431937	-6.12
520	CHEMBL3431845	COc1ccc(Cl)cc1S(=O)(N2C[C@](C([O-])=O)Cc3c2cc(C(Nc4ccccc4)=O)cc3)=O	CHEMBL3431937	-5.74
521	CHEMBL3431846	Cc1cc(c2nc([C@]3CCCCN3C(COc4ccccc4)=O)nn2)n(C)n1	CHEMBL3431937	-5.28
522	CHEMBL3431847	c1ccc(OCC(N2CCCC[C@@]2c3csc(c4cccc(C([O-])=O)c4)n3)=O)cc1	CHEMBL3431937	-5.63
523	CHEMBL3431848	CS(=O)(c1ccc(c2csc([C@]3CCCCN3C(COc4ccccc4)=O)n2)cc1)=O	CHEMBL3431937	-5.68
524	CHEMBL3431854	c1ccc(OCC(N2CCCC[C@@]2c3nc(c4ccc(C#N)cc4)cs3)=O)cc1	CHEMBL3431937	-5.71
525	CHEMBL3431856	c1ccc(OCC(N2CCCC[C@@]2c3nc(c4ccc(Cl)nc4)cs3)=O)cc1	CHEMBL3431937	-5.90
526	CHEMBL3431859	c1ccc(OCC(N2CCCC[C@@]2c3nc(c4ccc(C(N)=O)cc4)cs3)=O)cc1	CHEMBL3431937	-5.26
527	CHEMBL3431860	CC(Nc1ccc(c2nc([C@]3CCCCN3C(COc4ccccc4)=O)cs2)cc1)=O	CHEMBL3431937	-5.91
528	CHEMBL3431864	c1ccc(OCC(N2CCCC[C@@]2c3csc(c4ccc([C@](F)(F)F)nc4)n3)=O)cc1	CHEMBL3431937	-5.88
529	CHEMBL3431868	Cc1ccc(c2nc([C@]3CCCCN3C(COc4ccccc4)=O)cs2)cn1	CHEMBL3431937	-5.68
530	CHEMBL3431872	COc1ccc(c2nc([C@]3CCCCN3C(COc4ccccc4)=O)cs2)cn1	CHEMBL3431937	-6.52
531	CHEMBL3431873	c1ccc(OCC(N2CCCC[C@@]2c3csc(c4ccccn4)n3)=O)cc1	CHEMBL3431937	-5.32

532	CHEMBL3431878	Cc1c(c4cccc(C([O-])=O)c4)nc([C@]2CCCCN2C(COc3ccccc3)=O)s1	CHEMBL3431937	-5.21
533	CHEMBL3431880	CN(S(=O)(c4cc(Cl)ccc4OC)=O)c1cc(C(Nc3ncc(CC([O-])=O)s3)=O)cc2c1OCO2	CHEMBL3431937	-5.65
534	CHEMBL3431881	CN(S(=O)(c4cc(Cl)ccc4OC)=O)c1cc(C(Nc3ccn(CC([O-])=O)n3)=O)cc2c1OCO2	CHEMBL3431937	-6.89
535	CHEMBL3431882	COc1ccc(Cl)cc1S(=O)(N2CCOc3c2cc(C(Nc4nc(CC([O-])=O)cs4)=O)cc3Cl)=O	CHEMBL3431937	-5.59
536	CHEMBL3431883	COc1ccc(Cl)cc1S(=O)(N2CCOc3c2cc(C(Nc4ccc(CC([O-])=O)cc4)=O)cc3Cl)=O	CHEMBL3431937	-5.49
537	CHEMBL3431884	CCN(S(=O)(c4cc(Cl)ccc4OC)=O)c1cc(C(Nc3nc(CC([O-])=O)cs3)=O)cc2c1OCO2	CHEMBL3431937	-5.47
538	CHEMBL3431886	CCN(S(=O)(c4cc(Cl)ccc4OC)=O)c1cc(C(Nc3ncc(CC([O-])=O)s3)=O)cc2c1OCO2	CHEMBL3431937	-5.49
539	CHEMBL3431888	CCCN(S(=O)(c4cc(Cl)ccc4OC)=O)c1cc(C(Nc3nc(CC([O-])=O)cs3)=O)cc2c1OCO2	CHEMBL3431937	-5.45
540	CHEMBL3431889	CCCN(S(=O)(c4cc(Cl)ccc4OC)=O)c1cc(C(Nc3ccc(CC([O-])=O)cc3)=O)cc2c1OCO2	CHEMBL3431937	-5.46
541	CHEMBL3431890	CCCN(S(=O)(c4cc(Cl)ccc4OC)=O)c1cc(C(Nc3ncc(CC([O-])=O)s3)=O)cc2c1OCO2	CHEMBL3431937	-5.45
542	CHEMBL3431895	COc1ccc(Cl)cc1S(=O)(N2COc3c2cc(C(Nc4ccc(C([O-])=O)cc4)=O)cc3F)=O	CHEMBL3431937	-5.66
543	CHEMBL3431896	COc1ccc(Cl)cc1S(=O)(N2COc3c2cc(C(Nc4ccc(C([O-])=O)c(F)c4)=O)cc3F)=O	CHEMBL3431937	-5.73
544	CHEMBL3431897	COc1ccc(Cl)cc1S(=O)(N2COc3c2cc(C(Nc4ccc(C([O-])=O)c(Cl)c4)=O)cc3F)=O	CHEMBL3431937	-5.75
545	CHEMBL3431898	C[C@]1CN(S(=O)(c4cc(Cl)ccc4OC)=O)c2c1ccc(C(Nc3ccc(C([O-])=O)c(F)c3)=O)c2	CHEMBL3431937	-5.70
546	CHEMBL3431899	C[C@]1CN(S(=O)(c4cc(Cl)ccc4OC)=O)c2c1ccc(C(Nc3ccc(C([O-])=O)c(Cl)c3)=O)c2	CHEMBL3431937	-5.75
547	CHEMBL3431900	C[C@]1CN(S(=O)(c4cc(Cl)ccc4OC)=O)c2c1ccc(C(Nc3ccc(C([O-])=O)cc3)=O)c2	CHEMBL3431937	-5.62
548	CHEMBL3431901	c1ccc(OCC(N2CCCC[C@@]2c3nc(c4cccc(OCC([O-])=O)c4)no3)=O)cc1	CHEMBL3431937	-6.43
549	CHEMBL3431902	c1ccc(OCC(N2CCCC[C@@]2c3nc(c4cccc(OCC(N)=O)c4)no3)=O)cc1	CHEMBL3431937	-5.60
550	CHEMBL3431903	COc1ccc(Cl)cc1S(=O)(N2CCOc3c2cc(C(Nc4ccc(C([O-])=O)cc4)=O)cc3F)=O	CHEMBL3431937	-5.61
551	CHEMBL3431904	c1ccc(OCC(N2CCCC[C@@]2c3nc(c4ccc(C#N)cc4)on3)=O)cc1	CHEMBL3431937	-5.26
552	CHEMBL3431905	c1ccc(OCC(N2CCCC[C@@]2c3nc(c4cccc(C#N)c4)on3)=O)cc1	CHEMBL3431937	-5.42
553	CHEMBL3431906	c1ccc(OCC(N2CCCC[C@@]2c3nc(c4cc(N)cnc4)on3)=O)cc1	CHEMBL3431937	-5.30
554	CHEMBL3431907	COc1ccc(Cl)cc1Sc2cnc3c2cc(C(Nc4ccc(C([O-])=O)cc4)=O)cc3	CHEMBL3431937	-6.75
555	CHEMBL3431908	Cn1cc(Sc4cc(Cl)ccc4OC)c2c1ccc(C(Nc3ccc(C([O-])=O)cc3)=O)c2	CHEMBL3431937	-6.28
556	CHEMBL3431909	Cn1cc(S(=O)(c4cc(Cl)ccc4OC)=O)c2c1ccc(C(Nc3ccc(C([O-])=O)cc3)=O)c2	CHEMBL3431937	-6.33
557	CHEMBL3431910	CC1NC(C(c2nc([C@]3CCCCN3C(COc4ccccc4)=O)on2)=CN1)=O	CHEMBL3431937	-5.52
558	CHEMBL3431911	c1ccc(OCC(N3C[C@@]2C[C@@]2[C@@]3c4nc(c5ccc(S(=O)(N)=O)cc5)no4)=O)cc1	CHEMBL3431937	-5.29
559	CHEMBL3431912	COc1ccc(Cl)cc1S(=O)(N2CCOc3c2cc(C(Nc4ccc(C([O-])=O)c(F)c4)=O)cc3F)=O	CHEMBL3431937	-5.78
560	CHEMBL3431913	COc1ccc(Cl)cc1S(=O)(N2CCOc3c2cc(C(Nc4ccc(C([O-])=O)c(Cl)c4)=O)cc3F)=O	CHEMBL3431937	-5.84
561	CHEMBL3431914	COc1ccc(Cl)cc1S(=O)(N2CCOc3c2cc(C(Nc4ccc(C([O-])=O)cc4)=O)cc3Cl)=O	CHEMBL3431937	-5.66
562	CHEMBL3431915	Cc1cc(C(Nc4ccc(C([O-])=O)cc4)=O)cc2c1OCCN2S(=O)(c3cc(Cl)ccc3OC)=O	CHEMBL3431937	-5.78
563	CHEMBL3431916	Cc1cc(C(Nc4ccc(C([O-])=O)cc4)=O)cc2c1OCN2S(=O)(c3cc(Cl)ccc3OC)=O	CHEMBL3431937	-5.73
564	CHEMBL3431917	COc1ccc(Cl)cc1S(=O)(N2COc3c2cc(C(Nc4ccc(C([O-])=O)cc4)=O)cc3Cl)=O	CHEMBL3431937	-5.41
565	CHEMBL3431918	COc1ccc(Cl)cc1S(=O)(N2CCOc3c2cc(C(Nc4ccc(C([O-])=O)cc4)=O)cc3[C@](F)(F)F)=O	CHEMBL3431937	-5.58
566	CHEMBL3431920	C[C@@](N(S(=O)(c4cc(Cl)ccc4OC)=O)c1cc(C(Nc3ncc(CC([O-])=O)s3)=O)cc2c1OCO2)C	CHEMBL3431937	-5.45
567	CHEMBL3431922	COCCN(S(=O)(c4cc(Cl)ccc4OC)=O)c1cc(C(Nc3nc(CC([O-])=O)cs3)=O)cc2c1OCO2	CHEMBL3431937	-5.94
568	CHEMBL3431926	COc1ccc(Cl)cc1S(=O)(N2CCOc3c2cc(C(Nc4ncc(CC([O-])=O)s4)=O)cc3Cl)=O	CHEMBL3431937	-5.51
569	CHEMBL3431927	COc1ccc(Cl)cc1S(=O)(N2CCOc3c2cc(C(Nc4ccn(CC([O-])=O)n4)=O)cc3Cl)=O	CHEMBL3431937	-7.52
570	CHEMBL595	N1C(=O)S[C@@](Cc3ccc(OCCc2ncc(CC)cc2)cc3)C1=O	CHEMBL3431937	-5.13

571	CHEMBL108877	c1(O)ccccc1Cl	CHEMBL905613	-4.47
572	CHEMBL11	N1(CCC[N@H+](C)C)c2c(cccc2)CCc3c1cccc3	CHEMBL905613	-4.54
573	CHEMBL110739	C1CC(=O)C=C2CC[C@@]3[C@]([C@@](O)C[C@@]4(C)[C@]3CC[C@@]4C(=O)CO)[C@@]12C	CHEMBL905613	-4.67
574	CHEMBL112	C(=O)(C)Nc1ccc(O)cc1	CHEMBL905613	-5.91
575	CHEMBL113	c12c(n(C)cn2)C(=O)N(C)C(=O)N1C	CHEMBL905613	-5.21
576	CHEMBL1143	c1(Cl)cc(Cl)ccc1O	CHEMBL905613	-4.58
577	CHEMBL13	O(C[C@](C[NH2+][C@@](C)C)O)c1ccc(CCOC)cc1	CHEMBL905613	-5.01
578	CHEMBL130	[N+]([O-])(=O)c1ccc(cc1)[C@@](O)[C@@](CO)NC(=O)[C@@](Cl)Cl	CHEMBL905613	-5.35
579	CHEMBL131	C1C(=O)C=C2[C@]([C@@]4[C@]([C@]3[C@]([C@](C(CO)=O)(O)CC3)(C)C[C@@]4O)CC2)(C)C=1	CHEMBL905613	-5.49
580	CHEMBL134	C1(=NCCN1)Nc2c(cccc2Cl)Cl	CHEMBL905613	-4.97
581	CHEMBL13888	[N+]([O-])(=O)c1cc(ccc1)O	CHEMBL905613	-4.56
582	CHEMBL14060	Oc1ccccc1	CHEMBL905613	-4.16
583	CHEMBL14068	[N+]([O-])(=O)c1cc(ccc1)N	CHEMBL905613	-4.35
584	CHEMBL14092	c1(ccccc1)c2ccccc2	CHEMBL905613	-5.39
585	CHEMBL14205	Oc1c(cccc1)[N+](=O)[O-]	CHEMBL905613	-4.05
586	CHEMBL14282	Nc1ccc(cc1)[N+](=O)[O-]	CHEMBL905613	-4.43
587	CHEMBL15063	n1c(nc(N[C@@](C)C)nc1NCC)Cl	CHEMBL905613	-4.40
588	CHEMBL154	c1([C@](C)C(=O)[O-])cc2c(cc1)cc(OC)cc2	CHEMBL905613	-5.29
589	CHEMBL15844	n1ccc2ccccc12	CHEMBL905612	-4.50
590	CHEMBL15888	Clc1ccc(cc1)N	CHEMBL905613	-4.12
591	CHEMBL16	N1C(=O)[C@](c2ccccc2)(c3ccccc3)NC=1O	CHEMBL905613	-4.58
592	CHEMBL1790041	[N+]([O-])(=O)\C=C(\NCCSCc1oc(C[N@H+](C)C)cc1)/NC	CHEMBL905613	-5.87
593	CHEMBL189558	O=C1[C@@](NC(=O)[C@](Cc3ccc(cc3)O)N1)Cc2ccc(cc2)O	CHEMBL905612	-6.17
594	CHEMBL190	N1(C)c2c(ncn2)C(=O)N(C)C1=O	CHEMBL905613	-5.40
595	CHEMBL191011	N1C(=O)[C@](Cc3cnc5c3cccc5)NC(=O)[C@@]1Cc2cnc4c2cccc4	CHEMBL905612	-5.04
596	CHEMBL207225	C([O-])(CCc1cnc2c1cccc2)=O	CHEMBL905612	-5.68
597	CHEMBL22	n1c(N)c(cnc1N)Cc2cc(OC)c(OC)c(OC)c2	CHEMBL905613	-5.46
598	CHEMBL226544	[C@]1(Cc2cnc3c2cccc3)C(NCC(N1)=O)=O	CHEMBL905612	-6.02
599	CHEMBL226545	c2ccc1c(c(CCO)cn1)c2	CHEMBL905612	-4.72
600	CHEMBL226650	c1cccc([C@@](CC)C)c1OC(NC)=O	CHEMBL905613	-4.57
601	CHEMBL226651	COC1=NN(CSP(OC)(OC)=S)C(S1)=O	CHEMBL905613	-4.76
602	CHEMBL226967	COP2(OCc1ccccc1O2)=S	CHEMBL905613	-4.80
603	CHEMBL226968	CCc1ccc(C(NN([C@](C)(C)C)C(c2cc(C)cc(C)c2)=O)=O)cc1	CHEMBL905612	-4.80
604	CHEMBL226969	C[C@](C)(N(NC(c2ccc(Cl)cc2)=O)C(c1ccccc1)=O)C	CHEMBL905612	-4.76
605	CHEMBL227020	Cc1cc(C)cc(C(N(NC(c2c(C)c3c(OCCC3)cc2)=O)[C@](C)(C)C)=O)c1	CHEMBL905612	-4.78
606	CHEMBL227122	c4cccc1c4nc(C[C@@](C([O-])=O)NC(=O)OC[C@]2c3c(c5c2cccc5)cccc3)c1	CHEMBL905612	-4.89
607	CHEMBL227123	[NH3+][C@@](Cc2cnc1c2cccc1)C(N)=O	CHEMBL905612	-5.56
608	CHEMBL227124	[NH3+]CC(=O)N[C@@](Cc1cnc2c1cccc2)C(N)=O	CHEMBL905612	-6.38
609	CHEMBL227173	[NH3+][C@@](Cc1cnc2c1cccc2)C(=O)N[C@@](C)C(=O)N[C@@]([C@@](C)C)C(N)=O	CHEMBL905612	-6.62

610	CHEMBL227174	N(C(C)=O)[C@@](Cc1cnc2c1cccc2)C(N[C@@]([C@@](C)C)C(N)=O)=O	CHEMBL905612	-6.18
611	CHEMBL227181	c1(C)ccc(CC)cn1	CHEMBL905613	-5.33
612	CHEMBL227265	c1cccc2c1nc(C[C@@](C([O-])=O)NC(=O)O[C@](C)(C)C)c2	CHEMBL905612	-5.77
613	CHEMBL227338	N(C(C)=O)[C@](Cc1cnc2c1cccc2)C(=O)N[C@@]([C@@](C)C)C(N)=O	CHEMBL905612	-6.14
614	CHEMBL227339	N(C(C)=O)[C@@](Cc1ccc(O)cc1)C(N[C@@](C[C@@](C)C)C(N)=O)=O	CHEMBL905612	-6.54
615	CHEMBL23	[C@]1(OC(C)=O)C(=O)N(CC[N@@H+](C)C)c3c(cccc3)S[C@]1c2ccc(OC)cc2	CHEMBL905613	-5.62
616	CHEMBL23832	c1cccc(Nc2ccccc2C(=O)[O-])c1	CHEMBL905613	-4.78
617	CHEMBL266195	O(C[C@@](O)C[NH2+][C@@](C)C)c1c(cccc1)CC=C	CHEMBL905613	-4.70
618	CHEMBL27	c12c(cccc1cccc2)OC[C@@](O)C[NH2+][C@@](C)C	CHEMBL905613	-4.53
619	CHEMBL274009	[N+]([O-])(=O)c1c(cccc1)N	CHEMBL905613	-4.24
620	CHEMBL277474	C1(=O)N(c2ccccc2)N(C)C(C)=C1	CHEMBL905613	-5.36
621	CHEMBL279564	c12c3c4ccc1cccc2ccc3ccc4	CHEMBL905613	-5.21
622	CHEMBL288470	C1(=O)C(N(C)C)=C(C)N(C)N1c2ccccc2	CHEMBL905613	-4.55
623	CHEMBL29878	c1(C)c(O)ccc(C)c1	CHEMBL905613	-4.27
624	CHEMBL323348	c1(C(OC)=O)c(C(OC)=O)cccc1	CHEMBL905613	-4.18
625	CHEMBL325415	c1c(cccc1Cl)N	CHEMBL905613	-4.15
626	CHEMBL35	NS(=O)(c1c(cc(c(C(=O)[O-])c1)NCc2ccco2)Cl)=O	CHEMBL905613	-6.40
627	CHEMBL354761	[N+]([O-])(=O)c1cc(Cl)ccc1Cl	CHEMBL905613	-4.77
628	CHEMBL374395	c1cccc2c1nc(C[C@@](C([O-])=O)NC(=O)OCc3ccccc3)c2	CHEMBL905612	-5.62
629	CHEMBL379099	c1cccc2c1ncc2C(N)=O	CHEMBL905612	-5.21
630	CHEMBL384467	C1=CC(=O)C=C2CC[C@@]3[C@@]([C@@](O)C[C@@]4(C)[C@]3C[C@@](C)[C@]4(O)C(=O)CO)(F)[C@@]12C	CHEMBL905613	-5.23
631	CHEMBL386630	C1CC(C=C2CC[C@@]3[C@](CC[C@@]4(C)[C@]3CC[C@@]4O)[C@@]12C)=O	CHEMBL905613	-4.59
632	CHEMBL38688	c1ccc(Nc2ccccc2)cc1	CHEMBL905613	-5.28
633	CHEMBL387527	c12ccccc1ncc2C(=O)[O-]	CHEMBL905612	-6.10
634	CHEMBL387744	c1cc(C(=O)NN([C@](C)(C)C)C(=O)c2ccccc2)ccc1	CHEMBL905612	-4.78
635	CHEMBL388382	c2c(C[C@@](C(N[C@@](Cc1ccccc1)C(N)=O)=O)NC(C)=O)ccc(O)c2	CHEMBL905612	-6.92
636	CHEMBL388384	c1ccccc1CSP(O[C@@](C)C)(=O)O[C@@](C)C	CHEMBL905613	-4.44
637	CHEMBL388558	c1(C(OCC)=O)c(C(OCC)=O)cccc1	CHEMBL905613	-4.66
638	CHEMBL388559	CCN(C(SCc1ccc(Cl)cc1)=O)CC	CHEMBL905613	-4.47
639	CHEMBL388560	n1c(C)cc(OP(OCC)(=S)OCC)nc1[C@@](C)C	CHEMBL905613	-5.02
640	CHEMBL389621	C2CC(=O)C=C1CC[C@@]3[C@]([C@@](O)C[C@@]4(C)[C@]3CC[C@]4(O)C(=O)CO)[C@]12C	CHEMBL905613	-5.32
641	CHEMBL389885	c1cccc(Cl)c1N	CHEMBL905613	-4.19
642	CHEMBL390937	c2cccc1c2c(C[C@@](C(N)=O)NC(C)=O)cn1	CHEMBL905612	-5.72
643	CHEMBL390938	[NH3+][C@@](Cc2ccccc2)C(=O)N[C@@](Cc1cnc3c1cccc3)C(N)=O	CHEMBL905612	-5.22
644	CHEMBL406819	c1cc(ncc1CN2CCNC2\=N[\N+]([O-])=O)Cl	CHEMBL905613	-5.32
645	CHEMBL424	c1(c(cccc1)O)C(=O)[O-]	CHEMBL905613	-5.72
646	CHEMBL429	c1(C(N)=O)cc([C@@](O)C[NH2+][C@@](C)CCc2ccccc2)ccc1O	CHEMBL905613	-5.20
647	CHEMBL435	NS(=O)(c1cc2c(NCNS2(=O)=O)cc1Cl)=O	CHEMBL905613	-6.57
648	CHEMBL46931	c1cccc(C)c1O	CHEMBL905613	-4.03

649	CHEMBL500	c12c(cccc1OC[C@@](O)C[NH2+][C@@](C)C)ncc2	CHEMBL905613	-5.49
650	CHEMBL521	C([O-])(=O)[C@@](C)c1ccc(C[C@@](C)C)cc1	CHEMBL905613	-4.49
651	CHEMBL527	S1(=O)(=O)N(C)C(C(=O)Nc3ncccc3)=C(O)c2c1cccc2	CHEMBL905613	-4.88
652	CHEMBL537	c1cc(O)ccc1O	CHEMBL905613	-5.20
653	CHEMBL538	Nc1ccccc1	CHEMBL905613	-3.92
654	CHEMBL546	O(C[C@@](O)C[NH2+][C@@](C)C)c1c(cccc1)OCC=C	CHEMBL905613	-4.90
655	CHEMBL55772	N(NC(=O)c2c(C)c(ccc2)OC)([C@](C)(C)C)C(=O)c1cc(C)cc(C)c1	CHEMBL905612	-4.72
656	CHEMBL571	C(=O)(c2ccccc2)c1cc(ccc1)[C@](C)C(=O)[O-]	CHEMBL905613	-5.50
657	CHEMBL642	c1(C(C)=O)c(ccc(c1)NC(CCC)=O)OC[C@@](O)C[NH2+][C@@](C)C	CHEMBL905613	-6.15
658	CHEMBL6466	C1(=O)C=Cc2ccccc2O1	CHEMBL905613	-4.22
659	CHEMBL649	c12C[C@](O)[C@](O)Cc2cccc1OC[C@@](O)C[NH2+][C@@](C)(C)C	CHEMBL905613	-5.60
660	CHEMBL66381	c1cc(C#N)ccc1O	CHEMBL905613	-4.73
661	CHEMBL6640	c2ccc1c(ncc1CC[NH3+])c2	CHEMBL905612	-5.27
662	CHEMBL6966	O(C)c1cc(ccc1OC)[C@](CCC[N@H+](C)CCc2cc(c(cc2)OC)OC)(C#N)[C@@](C)C	CHEMBL905613	-4.41
663	CHEMBL6995	C(=O)(C)Nc1ccc(cc1)OC[C@](O)C[NH2+][C@@](C)C	CHEMBL905613	-6.13
664	CHEMBL72	N1(CCC[NH2+]C)c2c(cccc2)CCc3c1cccc3	CHEMBL905613	-4.45
665	CHEMBL82411	c12c(c(CC(=O)[O-])cn1)cccc2	CHEMBL905612	-6.40
666	CHEMBL9	c1(N3CC[NH2+]CC3)c(F)cc2c(N(CC)C=C(C(=O)[O-])C2=O)c1	CHEMBL905613	-6.72
667	CHEMBL9967	N1(C(=O)C[N@H+]2CC[N@H+](C)CC2)c3c(cccc3)C(=O)Nc4c1nccc4	CHEMBL905613	-6.06
668	CHEMBL108877	c1(O)ccccc1Cl	CHEMBL905613	-4.82
669	CHEMBL11	N1(CCC[N@H+](C)C)c2c(cccc2)CCc3c1cccc3	CHEMBL905613	-4.20
670	CHEMBL110739	C1CC(=O)C=C2CC[C@@]3[C@]([C@@](O)C[C@@]4(C)[C@]3CC[C@@]4C(=O)CO)[C@@]12C	CHEMBL905613	-4.47
671	CHEMBL112	C(=O)(C)Nc1ccc(O)cc1	CHEMBL905613	-5.65
672	CHEMBL113	c12c(n(C)cn2)C(=O)N(C)C(=O)N1C	CHEMBL905613	-4.80
673	CHEMBL1143	c1(Cl)cc(Cl)ccc1O	CHEMBL905613	-3.90
674	CHEMBL13	O(C[C@](C[NH2+][C@@](C)C)O)c1ccc(CCOC)cc1	CHEMBL905613	-4.84
675	CHEMBL130	[N+]([O-])(=O)c1ccc(cc1)[C@@](O)[C@@](CO)NC(=O)[C@@](Cl)Cl	CHEMBL905613	-5.24
676	CHEMBL131	C1C(=O)C=C2[C@]([C@@]4[C@]([C@]3[C@]([C@](C(CO)=O)(O)CC3)(C)C[C@@]4O)CC2)(C)C=1	CHEMBL905613	-5.52
677	CHEMBL134	C1(=NCCN1)Nc2c(cccc2Cl)Cl	CHEMBL905613	-4.94
678	CHEMBL13888	[N+]([O-])(=O)c1cc(ccc1)O	CHEMBL905613	-4.16
679	CHEMBL14060	Oc1ccccc1	CHEMBL905613	-3.83
680	CHEMBL14068	[N+]([O-])(=O)c1cc(ccc1)N	CHEMBL905613	-3.71
681	CHEMBL14092	c1(ccccc1)c2ccccc2	CHEMBL905613	-4.27
682	CHEMBL14205	Oc1c(cccc1)[N+](=O)[O-]	CHEMBL905613	-3.66
683	CHEMBL14282	Nc1ccc(cc1)[N+](=O)[O-]	CHEMBL905613	-3.91
684	CHEMBL15063	n1c(nc(N[C@@](C)C)nc1NCC)Cl	CHEMBL905613	-4.14
685	CHEMBL154	c1([C@](C)C(=O)[O-])cc2c(cc1)cc(OC)cc2	CHEMBL905613	-5.26
686	CHEMBL15888	Clc1ccc(cc1)N	CHEMBL905613	-3.59
687	CHEMBL16	N1C(=O)[C@](c2ccccc2)(c3ccccc3)NC=1O	CHEMBL905613	-4.93

688	CHEMBL1790041	[N+]([O-])(=O)\C=C(\NCCSCc1oc(C[N@H+](C)C)cc1)/NC	CHEMBL905613	-5.52
689	CHEMBL190	N1(C)c2c(ncn2)C(=O)N(C)C1=O	CHEMBL905613	-5.58
690	CHEMBL22	n1c(N)c(cnc1N)Cc2cc(OC)c(OC)c(OC)c2	CHEMBL905613	-5.38
691	CHEMBL226650	c1cccc([C@@](CC)C)c1OC(NC)=O	CHEMBL905613	-3.88
692	CHEMBL226651	COC1=NN(CSP(OC)(OC)=S)C(S1)=O	CHEMBL905613	-4.33
693	CHEMBL226967	COP2(OCc1ccccc1O2)=S	CHEMBL905613	-4.36
694	CHEMBL227181	c1(C)ccc(CC)cn1	CHEMBL905613	-4.89
695	CHEMBL23	[C@]1(OC(C)=O)C(=O)N(CC[N@@H+](C)C)c3c(cccc3)S[C@]1c2ccc(OC)cc2	CHEMBL905613	-3.91
696	CHEMBL23832	c1cccc(Nc2ccccc2C(=O)[O-])c1	CHEMBL905613	-4.61
697	CHEMBL266195	O(C[C@@](O)C[NH2+][C@@](C)C)c1c(cccc1)CC=C	CHEMBL905613	-4.21
698	CHEMBL27	c12c(cccc1cccc2)OC[C@@](O)C[NH2+][C@@](C)C	CHEMBL905613	-4.42
699	CHEMBL274009	[N+]([O-])(=O)c1c(cccc1)N	CHEMBL905613	-3.64
700	CHEMBL277474	C1(=O)N(c2ccccc2)N(C)C(C)=C1	CHEMBL905613	-5.00
701	CHEMBL279564	c12c3c4ccc1cccc2ccc3ccc4	CHEMBL905613	-4.32
702	CHEMBL288470	C1(=O)C(N(C)C)=C(C)N(C)N1c2ccccc2	CHEMBL905613	-4.14
703	CHEMBL29878	c1(C)c(O)ccc(C)c1	CHEMBL905613	-3.58
704	CHEMBL323348	c1(C(OC)=O)c(C(OC)=O)cccc1	CHEMBL905613	-3.80
705	CHEMBL325415	c1c(cccc1Cl)N	CHEMBL905613	-3.58
706	CHEMBL35	NS(=O)(c1c(cc(c(C(=O)[O-])c1)NCc2ccco2)Cl)=O	CHEMBL905613	-6.24
707	CHEMBL354761	[N+]([O-])(=O)c1cc(Cl)ccc1Cl	CHEMBL905613	-4.09
708	CHEMBL384467	C1=CC(=O)C=C2CC[C@@]3[C@@]([C@@](O)C[C@@]4(C)[C@]3C[C@@](C)[C@]4(O)C(=O)CO)(F)[C@@]12C	CHEMBL905613	-4.95
709	CHEMBL386630	C1CC(C=C2CC[C@@]3[C@](CC[C@@]4(C)[C@]3CC[C@@]4O)[C@@]12C)=O	CHEMBL905613	-3.87
710	CHEMBL38688	c1ccc(Nc2ccccc2)cc1	CHEMBL905613	-4.82
711	CHEMBL388384	c1ccccc1CSP(O[C@@](C)C)(=O)O[C@@](C)C	CHEMBL905613	-3.44
712	CHEMBL388558	c1(C(OCC)=O)c(C(OCC)=O)cccc1	CHEMBL905613	-4.33
713	CHEMBL388559	CCN(C(SCc1ccc(Cl)cc1)=O)CC	CHEMBL905613	-4.03
714	CHEMBL388560	n1c(C)cc(OP(OCC)(=S)OCC)nc1[C@@](C)C	CHEMBL905613	-4.51
715	CHEMBL389621	C2CC(=O)C=C1CC[C@@]3[C@]([C@@](O)C[C@@]4(C)[C@]3CC[C@]4(O)C(=O)CO)[C@]12C	CHEMBL905613	-5.07
716	CHEMBL389885	c1cccc(Cl)c1N	CHEMBL905613	-3.61
717	CHEMBL406819	c1cc(ncc1CN2CCNC2\=N[\N+]([O-])=O)Cl	CHEMBL905613	-5.28
718	CHEMBL424	c1(c(cccc1)O)C(=O)[O-]	CHEMBL905613	-5.31
719	CHEMBL429	c1(C(N)=O)cc([C@@](O)C[NH2+][C@@](C)CCc2ccccc2)ccc1O	CHEMBL905613	-5.25
720	CHEMBL435	NS(=O)(c1cc2c(NCNS2(=O)=O)cc1Cl)=O	CHEMBL905613	-6.54
721	CHEMBL46931	c1cccc(C)c1O	CHEMBL905613	-3.53
722	CHEMBL500	c12c(cccc1OC[C@@](O)C[NH2+][C@@](C)C)ncc2	CHEMBL905613	-5.85
723	CHEMBL521	C([O-])(=O)[C@@](C)c1ccc(C[C@@](C)C)cc1	CHEMBL905613	-4.14
724	CHEMBL527	S1(=O)(=O)N(C)C(C(=O)Nc3ncccc3)=C(O)c2c1cccc2	CHEMBL905613	-4.72
725	CHEMBL537	c1cc(O)ccc1O	CHEMBL905613	-5.34
726	CHEMBL538	Nc1ccccc1	CHEMBL905613	-3.52

727	CHEMBL546	O(C[C@@](O)C[NH2+][C@@](C)C)c1c(cccc1)OCC=C	CHEMBL905613	-5.03
728	CHEMBL571	C(=O)(c2ccccc2)c1cc(ccc1)[C@](C)C(=O)[O-]	CHEMBL905613	-5.40
729	CHEMBL642	c1(C(C)=O)c(ccc(c1)NC(CCC)=O)OC[C@@](O)C[NH2+][C@@](C)C	CHEMBL905613	-5.58
730	CHEMBL6466	C1(=O)C=Cc2ccccc2O1	CHEMBL905613	-3.56
731	CHEMBL649	c12C[C@](O)[C@](O)Cc2cccc1OC[C@@](O)C[NH2+][C@@](C)(C)C	CHEMBL905613	-4.49
732	CHEMBL66381	c1cc(C#N)ccc1O	CHEMBL905613	-4.65
733	CHEMBL6966	O(C)c1cc(ccc1OC)[C@](CCC[N@H+](C)CCc2cc(c(cc2)OC)OC)(C#N)[C@@](C)C	CHEMBL905613	-3.96
734	CHEMBL6995	C(=O)(C)Nc1ccc(cc1)OC[C@](O)C[NH2+][C@@](C)C	CHEMBL905613	-6.43
735	CHEMBL72	N1(CCC[NH2+]C)c2c(cccc2)CCc3c1cccc3	CHEMBL905613	-3.81
736	CHEMBL9	c1(N3CC[NH2+]CC3)c(F)cc2c(N(CC)C=C(C(=O)[O-])C2=O)c1	CHEMBL905613	-6.75
737	CHEMBL9967	N1(C(=O)C[N@H+]2CC[N@H+](C)CC2)c3c(cccc3)C(=O)Nc4c1nccc4	CHEMBL905613	-6.09
738	BDH_33697220	C(c2cc4c(CCC4)cc2)(=O)N1CC[C@@]3(CC1)CNC(COc5c(cccc5)CC=CC3)=O	Astellas	-4.52
739	BDH_33701281	C(N2CC[C@@]3(CC2)C(=O)NCCOc4c(cccc4)C(=O)NCC=CC3)(=O)c1ncc[n+]1	Astellas	-6.52
740	BDH_33702300	c1(cc(C[C@](C)C)no1)C(=O)N2CC[C@]3(CC2)C(=O)NCCNC(=O)c4c(cccc4)OCCCC3	Astellas	-4.64
741	LAS_51647569	n1cnn(c1)CC(N2CC[C@@]3(CC2)C(=O)N(C)CCOc4c(CCCCC3)cccc4)=O	Astellas	-4.63
742	LAS_51649260	c2cnc4c(CN(Cc3cccc(OC)c3OC)CCCCCOc1c(cccc1)O4)c2	Astellas	-4.54
743	LAS_51658240	c1cnc2c(CN(Cc4c(OC)c(OC)ccc4)CCOCCOc3c(cccc3)O2)c1	Astellas	-4.56
744	LAS_51663301	c1(C(N2CC[C@]3(C(=O)NCCOCc4c[n+](nn4)CCCC3)CC2)=O)cn(CCC)nc1	Astellas	-6.05
745	LAS_51900482	[C@@]1(N3CC[C@]2(CC3)CCCCc4c(cccc4)OCCCC(=O)N(C2)C)CCOCC1	Astellas	-4.60
746	LAS_51900724	N(C)(C)CCCNC(=O)[C@@]2NC(=O)c1ccccc1OC[C@@]([C@](C)C)NC(=O)[C@@]([C@](C)C)NC(=O)[C@](C[C@](C)C)NC(=O)CC2	Astellas	-5.84
747	LAS_51904053	N1(CCOCC1)CCCNC(=O)[C@]3NC(=O)c2c(OCCN(C)C(=O)[C@@]([C@](C)C)NC(=O)C3)cccc2	Astellas	-5.68
748	LAS_52042692	c1(OCC)ccc(CN4Cc3c[n+](CC(=O)NCCOc2ccccc2C4)nn3)cc1	Astellas	-4.55
749	LAS_52042848	[C@@]1(N4CC(=O)Nc2cc(Cl)ccc2Oc3ccccc3OCCOCC4)CCOCC1	Astellas	-4.57
750	LAS_52044571	[n+]1(CC(N3CC[C@]4(CC3)CNC(=O)COc2c(C[C@@](O)[C@](O)C4)cccc2)=O)c([C@@](C)C)ncc1	Astellas	-5.20
751	LAS_52104235	C1(=O)NCCCOc3c(cccc3)C(=O)N[C@@](C(=O)O)CCC(=O)N[C@@]1Cc2ccccc2	Astellas	-5.82
752	LAS_52105209	N1(C)CCCN(C(=O)[C@@]2NC(=O)c3c(cccc3)OCCCNC(=O)[C@](C[C@](C)C)NC(=O)CC2)CC1	Astellas	-5.69
753	LAS_52107944	C1C[C@@](CCN1Cc6ccc(cc6)F)C(=O)N5C[C@@]3CC[C@@](CCN(c4ccccc4C5)C(=O)C)N3Cc2ccncc2	Astellas	-4.59
754	LAS_52110813	O1CCN(C(O[C@@](C)(C)C)=O)CCN(CC)C(=O)c3c(nccc3)Oc2c1cccc2	Astellas	-4.59
755	LAS_52132208	C1(=O)N[C@@](Cc4ccccc4)COc3c(cccc3)C(=O)NCCCOc2c(cccc2)C(=O)N[C@@](C(O)=O)CC1	Astellas	-6.10
756	LAS_52135120	[C@]2(CNC(=O)[C@@]4NC(=O)c3c(OC[C@@]([C@](C)C)NC(=O)[C@]5(CCCC5)NC(=O)CC4)cccc3)(CCOCC2)c1cc(ccc1)F	Astellas	-4.80
757	LAS_52135200	[C@]2(CNC(=O)[C@@]4NC(=O)c3c(OC[C@@]([C@](C)C)NC(=O)[C@@]5(CCCCC5)NC(=O)CC4)cccc3)(CCOCC2)c1cc(ccc1)F	Astellas	-4.63
758	LAS_52137850	c1(OCCC)cc(c2ccc3c(CN4[C@](CCC4)C(=O)NCCOCCO3)c2)ccc1	Astellas	-4.52
759	LAS_52141146	c1cnc2c(C(NCCCN4CCOCC4)=NCCCCCCOc3c(cccc3)O2)c1	Astellas	-5.63
760	LAS_52141152	c1cnc2c(C(N4CCN(CC4)[C@@](C)C)=NCCCCCCOc3c(cccc3)O2)c1	Astellas	-4.91
761	LAS_52144084	c2(N(C)CCc4ccccc4)ccc3c(CN1[C@](C(=O)NCCOCCO3)CCC1)c2	Astellas	-4.52
762	LAS_52146431	c1(c2ccc3c(CN4C[C@](C(=O)NCCOCCO3)CCC4)c2)c(F)c(OC)ccc1	Astellas	-4.54
763	LAS_52146600	C(=O)(N1CC[C@@]3(COc2ccccc2C(=O)NCCOCCCC3)CC1)CCN(C)C	Astellas	-5.46
764	LAS_52146805	n1c(O)cc(C(N2CC[C@@]4(C(=O)NCCOc3ccccc3CCCCC4)CC2)=O)cc1	Astellas	-5.84
765	LAS_52152793	N1(CCCNC3c2c(Oc4ccccc4OCCCCCN3)nccc2)CCOCC1	Astellas	-5.78

766	LAS_52154828	C1(=O)N[C@@](CCC(=O)N[C@]3(CCCC3)C(=O)N[C@]([C@@](C)C)COc4c1cccc4)C(NCCCc2nc(C)cs2)=O	Astellas	-4.70
767	LAS_52155805	C1(=O)N[C@](CCC(=O)N[C@@]3(CCCC3)C(=O)N[C@]([C@@](C)C)COc4c1cccc4)C(=O)NCc5cc(ccc5)CN2C(CCC2)=O	Astellas	-5.34
768	LAS_52155968	c2(Nc4cccc(OC)c4)ccc1c(CN3C[C@@](C(NCCOCCO1)=O)CCC3)c2	Astellas	-4.61
769	LAS_52159132	C(CC(N4Cc3ccccc3N(C(COC)=O)CCCN(Cc5ccc(Cl)cc5)CCC4)=O)[n+]1ncc2c1cccc2	Astellas	-4.53
770	LAS_52162509	c1(ccccc1Cl)CC(N4Cc2c(Oc3ccccc3OCCOCC4)nccc2)=O	Astellas	-4.55
771	LAS_52164169	c1(ccccc1)OCC(N2CC[C@]4(CN(C)C(COc3ccccc3C[C@@](O)[C@](O)C4)=O)CC2)=O	Astellas	-5.17
772	LAS_52166734	c2ccc1OCCCNC([C@@](Cc3ccccc3)NC([C@@](NC(C[C@@](NC(c1c2)=O)C(=O)O)=O)C[C@](C)C)=O)=O	Astellas	-5.81
773	LAS_52167560	C1(=O)N[C@@](C(NCCCc2cc(OC)c(OC)cc2)=O)CC(=O)N[C@@]([C@](C)C)C(=O)N[C@](Cc4ccccc4)C(=O)NCCCOc3c1cccc3	Astellas	-5.55
774	LAS_52167954	CCC(=O)N3CCOc4ccc(c1ccncc1)cc4Cc2cccc(C(NCC3)=O)c2	Astellas	-4.88
775	LAS_52168541	c1(ccccc1OC)CC(N3Cc2c(OCCCCCNC(=O)C3)c(OCC)ccc2)=O	Astellas	-4.62
776	LAS_52168719	c1(cc(C)ccc1)CC(N4Cc2c(Oc3ccccc3OCCCCCC4)nccc2)=O	Astellas	-4.52
777	LAS_52169928	C1(=O)N[C@](C(=O)NC[C@]4(CCOCC4)c3cc(ccc3)F)CC(=O)N[C@@]2(CCCCC2)C(=O)N[C@@](Cc6ccccc6)C(=O)NCCCOc5c1cccc5	Astellas	-4.77
778	LAS_52169970	C1(=O)N[C@](C(NCCOc2c(C)cc(C)cc2)=O)CC(=O)N[C@]3(CCCCC3)C(=O)N[C@@](Cc5ccccc5)C(=O)NCCCOc4c1cccc4	Astellas	-4.55
779	LAS_52170106	C1(=O)N[C@@](C(=O)NCc2ccc(cc2)CN4CCCC4)CC(=O)N[C@]3(CCCC3)C(=O)N[C@](Cc6ccccc6)C(=O)NCCCOc5c1cccc5	Astellas	-5.80
780	LAS_52172509	c1(OCC(N2CC[C@](O)[C@](O)COc3c(cccc3)CCCC2)=O)ccc(Cl)cc1	Astellas	-4.56
781	LAS_52172754	c1(C(N2CCCC[C@@]3(CO)CCN(CC3)Cc4c(cccc4)OCC2)=O)c(N)nccn1	Astellas	-6.22
782	LAS_52172839	c1(C(N2CCCC[C@@]3(CO)CCN(CC3)C(=O)c4c(cccc4)OCC2)=O)cccnc1	Astellas	-6.13
783	LAS_52466348	c1c(Cl)ccc(C(N4Cc2c(Oc3ccccc3OCCCCCC4)nccc2)=O)c1	Astellas	-4.54
784	LAS_52480202	C1N(C(=O)c2ccc3c(Cc4cc(ccc4)CN(C[C@@]5CC5)CCCCCO3)c2)CCOC1	Astellas	-4.60
785	LAS_52482819	c1(cnc[n+]1)C(N2CC[C@]3(CCCCOCCN(C)Cc4c(cccc4)OC3)CC2)=O	Astellas	-6.00
786	LAS_52506402	C(N(C)C)C(N3C[C@]2Oc1ccccc1C(N(CCCCCCCCO[C@@]2C3)C)=O)=O	Astellas	-4.91
787	LAS_52507024	c1(C(N3CCCCCCN(C)C(=O)c2c(O[C@]4[C@@]3CCCC4)cccc2)=O)cn(C)cn1	Astellas	-5.24
788	LAS_52511076	[C@]1(CC(N4[C@@]2CCCC[C@]2Oc3c(C(=O)N(CC)CCCCC4)cccc3)=O)CCCC1	Astellas	-4.52
789	LAS_52511151	c1(C(N4[C@@]2CCCC[C@@]2Oc3c(C(=O)N(C)CCCCC4)cccc3)=O)nn(C)c(C)c1	Astellas	-5.33
790	LAS_52514101	c1(C(N3CCCCCN(CC)C(=O)c2c(cccc2)OC[C@@]3[C@@](C)C)=O)nc(N)sc1	Astellas	-5.27
791	LAS_52515343	c1(C(N2[C@](C[C@@](C)C)COc3c(C(N(C)CCCCCC2)=O)cccc3)=O)c[n+](C)nc1	Astellas	-4.84
792	LAS_52515710	c1(cncn1)C(N4[C@@]3[C@](CCCC3)OC2=CCC=C[C@@]2C(N(C)CCCCCCC4)=O)=O	Astellas	-4.64
793	LAS_52515717	[C@@](C)(C)CC(N3[C@@]2[C@](CCCC2)OC1=CCC=C[C@@]1C(N(C)CCCCCCC3)=O)=O	Astellas	-4.52
794	AC_9A54	N6[C@](C(N[C@@]([C@@](C)(C)C)C(N[C@](C(N[C@](C(N[C@](C(N[C@](C(N[C@](Cc3ccc4c(cccc4)c3)C(N[C@](C(N[C@](C(N[C@](CCCC)C(N[C@](C6=O)CCC(N)=O)=O)=O)Cc5cc(c(F)cc5)F)=O)CCC\N=C(\N)N)=O)=O)CCCN=C(N)N)=O)CCCN=C(N)N)=O)CCCN=C(N)N)=O)[C@](C)C)=O)=O)Cc1cnc2c1cccc2	Enamine	-7.00
795	AC_9A5	N1[C@](C(N[C@@]([C@](O)C)C(N[C@](C(N[C@](C(N[C@](C(N[C@](C(N[C@](Cc4ccc5c(cccc5)c4)C(N[C@](C(N[C@](C(N[C@](CCCC)C(N[C@](C1=O)CCC(N)=O)=O)=O)Cc6ccc(F)cc6)=O)CCCNC(=N)N)=O)=O)CCCNC(=N)N)=O)CCCNC(N)=N)=O)CCCN\C(=N)\N)=O)C)=O)=O)Cc2cnc3c2cccc3	Enamine	-7.20

Table S2 Experimental solubility and Log*D* values of macro-cyclic compounds

Number	Compound name	Solubility (x 10^{-6} mol/L)		$LogD_{7.4}$
		Solubility in JP1[a]	Solubility in JP2[b]	
1	BDH 33697220	11.2	9.6	4.5
2	BDH 33701281	>=100	>=100	1.2
3	BDH 33701755	>=100	>=100	1.1
4	BDH 33702300	69.5	54.7	3.1
5	LAS 51641052	No data	No data	No data
6	LAS 51647569	>=100	>=100	2.4
7	LAS 51649260	>=100	>=100	3.4
8	LAS 51658240	>=100	>=100	2
9	LAS 51663301	>=100	>=100	0.2
10	LAS 51900229	No data	No data	No data
11	LAS 51900259	No data	No data	No data
12	LAS 51900482	>=100	>=100	2.1
13	LAS 51900714	No data	No data	No data
14	LAS 51900724	>=100	>=100	1
15	LAS 51903074	No data	No data	No data
16	LAS 51904053	>=100	>=100	0.3
17	LAS 52042692	>=100	6.3	3.3
18	LAS 52042848	>=100	39.7	3.9
19	LAS 52044571	>=100	>=100	0.2
20	LAS 52096562	No data	No data	No data
21	LAS 52096588	No data	No data	No data
22	LAS 52103304	No data	No data	No data
23	LAS 52103320	No data	No data	No data
24	LAS 52104235	>=100	>=100	-0.8
25	LAS 52105209	>=100	>=100	0.4
26	LAS 52105501	No data	No data	No data
27	LAS 52107944	>=100	>=100	2.8
28	LAS 52110813	41.9	>=100	3.2
29	LAS 52132208	1.6	>=100	0.1
30	LAS 52135120	>=100	>=100	3.6
31	LAS 52135200	>=100	>=100	4
32	LAS 52137850	>=100	1.8	4.8
33	LAS 52141146	>=100	>=100	0.4

34	LAS 52141152	>=100	>=100	1.1
35	LAS 52144084	>=100	>=100	4
36	LAS 52146431	>=100	>=100	3.7
37	LAS 52146600	>=100	>=100	0.5
38	LAS 52146805	>=100	>=100	2.1
39	LAS 52152793	>=100	>=100	0.3
40	LAS 52154828	>=100	>=100	4
41	LAS 52155805	>=100	>=100	3
42	LAS 52155968	>=100	>=100	3.1
43	LAS 52159132	>=100	6	4.4
44	LAS 52159509	<1	<1	>6.1
45	LAS 52162509	1.1	<1	3.3
46	LAS 52163178	No data	No data	No data
47	LAS 52164169	>=100	>=100	1.4
48	LAS 52166734	>=100	>=100	0.1
49	LAS 52167560	13.7	16.6	3.3
50	LAS 52167954	>=100	<1	2.9
51	LAS 52168541	5.9	7.8	3.4
52	LAS 52168719	<1	<1	4.9
53	LAS 52169928	67.3	57.6	4.2
54	LAS 52169970	3.9	2.9	5.2
55	LAS 52170082	No data	No data	No data
56	LAS 52170106	>=100	>=100	1.9
57	LAS 52172509	61.5	50	3.5
58	LAS 52172754	>=100	>=100	0.4
59	LAS 52172839	>=100	>=100	1.5
60	LAS 52453086	No data	No data	No data
61	LAS 52466348	<1	<1	>6
62	LAS 52480202	>=100	>=100	2.6
63	LAS 52482819	>=100	>=100	0.7
64	LAS 52506402	>=100	>=100	1.5
65	LAS 52507024	>=100	>=100	2.4
66	LAS 52511076	>=100	>=100	4.1
67	LAS 52511151	>=100	>=100	2.4
68	LAS 52514101	>=100	>=100	2
69	LAS 52515343	>=100	>=100	2.6
70	LAS 52515710	>=100	>=100	3

71	LAS 52515717	>=100	>=100	4.3

a : 1st Fluid for disintegration test, pH 1.2/1st Fluid for dissolution test, pH 1.2

b : 2nd Fluid for disintegration test, pH 6.8/ 2nd Fluid for dissolution test, pH 6.8

Table S3 Experimental and predicted data by the 4-fold cross validation

No.	Compound ID	LogP_{app}			
		Exptl	Model A	Model A	Model AB
1	CHEMBL121893	-2.67	-3.50	-3.11	-3.29
2	CHEMBL538150	-2.85	-3.45	-3.49	-3.62
3	CHEMBL539139	-3.80	-3.98	-3.78	-3.78
4	CHEMBL539393	-2.91	-3.27	-3.15	-3.24
5	CHEMBL539718	-3.22	-3.24	-3.30	-3.35
6	CHEMBL540227	-5.70	-4.06	-3.95	-4.15
7	CHEMBL540471	-2.75	-3.53	-3.50	-3.46
8	CHEMBL541478	-3.32	-3.53	-3.81	-3.80
9	CHEMBL541481	-2.69	-3.32	-3.49	-3.31
10	CHEMBL549482	-3.14	-3.17	-3.04	-3.10
11	CHEMBL549483	-3.19	-3.19	-3.13	-3.23
12	CHEMBL549557	-3.14	-3.06	-3.31	-3.33
13	CHEMBL549561	-3.00	-3.42	-3.68	-3.54
14	CHEMBL549562	-3.66	-3.28	-3.42	-3.29
15	CHEMBL549685	-3.64	-3.03	-3.11	-3.27
16	CHEMBL549889	-3.32	-3.53	-3.54	-3.69
17	CHEMBL550090	-2.94	-3.03	-3.24	-2.95
18	CHEMBL550094	-3.16	-3.71	-3.25	-3.35
19	CHEMBL550295	-2.96	-3.91	-3.66	-3.81
20	CHEMBL550497	-3.03	-3.42	-3.38	-3.47
21	CHEMBL550752	-2.98	-3.41	-3.28	-3.31
22	CHEMBL550758	-3.16	-3.56	-3.31	-3.50
23	CHEMBL550760	-3.80	-3.59	-3.38	-3.38
24	CHEMBL550761	-3.42	-3.36	-3.28	-3.21
25	CHEMBL550765	-2.90	-3.11	-3.36	-3.31
26	CHEMBL550766	-3.04	-3.32	-3.59	-3.54
27	CHEMBL550767	-3.12	-3.35	-3.35	-3.31
28	CHEMBL550768	-3.44	-3.29	-3.35	-3.38
29	CHEMBL550841	-6.70	-3.82	-3.90	-3.95
30	CHEMBL550845	-2.98	-3.53	-3.36	-3.53
31	CHEMBL550905	-6.00	-3.51	-3.38	-3.41
32	CHEMBL550954	-3.25	-3.53	-3.43	-3.52
33	CHEMBL551046	-2.89	-3.85	-3.96	-3.93

34	CHEMBL551106	-2.81	-3.31	-3.32	-3.29
35	CHEMBL551184	-3.02	-3.41	-3.40	-3.48
36	CHEMBL551185	-2.98	-3.31	-3.09	-3.12
37	CHEMBL551244	-3.85	-3.93	-4.03	-4.17
38	CHEMBL551385	-4.52	-3.75	-3.76	-3.78
39	CHEMBL551450	-2.64	-2.79	-3.10	-2.89
40	CHEMBL551710	-3.08	-3.26	-3.35	-3.15
41	CHEMBL551791	-4.10	-3.44	-3.33	-3.38
42	CHEMBL551962	-6.00	-3.64	-3.67	-3.73
43	CHEMBL551975	-2.87	-3.63	-3.52	-3.60
44	CHEMBL551976	-3.13	-3.88	-3.68	-3.63
45	CHEMBL551979	-3.72	-3.04	-3.44	-3.10
46	CHEMBL552048	-2.81	-3.61	-3.30	-3.36
47	CHEMBL552105	-2.90	-3.19	-3.04	-3.36
48	CHEMBL552106	-2.84	-3.21	-3.47	-3.41
49	CHEMBL552158	-3.16	-3.24	-3.26	-3.22
50	CHEMBL552185	-3.18	-3.34	-3.41	-3.36
51	CHEMBL552392	-3.15	-3.57	-3.65	-3.42
52	CHEMBL552517	-3.85	-3.51	-3.43	-3.22
53	CHEMBL552742	-3.77	-3.81	-3.76	-3.68
54	CHEMBL553190	-2.83	-3.24	-3.42	-3.16
55	CHEMBL553624	-3.03	-3.24	-3.23	-3.16
56	CHEMBL553652	-2.89	-3.89	-3.34	-3.88
57	CHEMBL555065	-3.09	-3.78	-3.38	-3.50
58	CHEMBL555726	-4.30	-3.85	-3.76	-3.52
59	CHEMBL555749	-3.64	-3.78	-3.65	-3.49
60	CHEMBL556270	-6.00	-3.83	-3.71	-3.76
61	CHEMBL557905	-3.07	-3.23	-3.41	-3.28
62	CHEMBL557906	-5.00	-3.37	-3.45	-3.42
63	CHEMBL558106	-3.30	-3.71	-3.28	-3.35
64	CHEMBL558107	-2.87	-2.72	-2.96	-2.72
65	CHEMBL558297	-3.42	-3.74	-3.89	-3.59
66	CHEMBL558298	-4.30	-3.80	-3.87	-3.61
67	CHEMBL558889	-3.25	-3.80	-3.68	-3.87
68	CHEMBL559520	-3.13	-3.23	-3.24	-3.22
69	CHEMBL559521	-4.16	-3.37	-3.27	-3.48
70	CHEMBL559522	-3.77	-3.37	-3.69	-3.44

71	CHEMBL559716	-3.34	-3.38	-3.07	-3.08
72	CHEMBL559717	-3.48	-3.48	-3.43	-3.59
73	CHEMBL559718	-3.09	-3.81	-3.77	-3.73
74	CHEMBL560108	-3.27	-3.87	-3.87	-3.76
75	CHEMBL560114	-3.07	-2.91	-2.78	-2.69
76	CHEMBL560148	-2.96	-3.08	-3.28	-3.20
77	CHEMBL560172	-2.98	-3.34	-3.24	-3.32
78	CHEMBL560309	-2.83	-3.78	-4.07	-4.03
79	CHEMBL560310	-3.50	-3.99	-4.01	-4.11
80	CHEMBL560311	-3.22	-3.50	-3.29	-3.44
81	CHEMBL560369	-2.85	-3.36	-3.55	-3.44
82	CHEMBL560510	-3.11	-3.18	-3.29	-3.10
83	CHEMBL560512	-3.08	-3.31	-3.67	-3.71
84	CHEMBL560568	-3.85	-3.32	-3.09	-3.21
85	CHEMBL560569	-2.64	-3.38	-3.43	-3.56
86	CHEMBL560675	-5.00	-4.54	-4.62	-4.73
87	CHEMBL560911	-3.25	-2.85	-2.97	-2.98
88	CHEMBL560972	-3.03	-3.58	-3.37	-3.44
89	CHEMBL561050	-3.27	-3.42	-3.43	-3.59
90	CHEMBL561051	-4.70	-3.74	-3.65	-3.49
91	CHEMBL561052	-3.46	-3.27	-3.25	-3.13
92	CHEMBL561170	-3.72	-3.45	-3.55	-3.48
93	CHEMBL561245	-2.75	-3.39	-3.31	-3.06
94	CHEMBL561446	-3.77	-3.29	-3.16	-3.06
95	CHEMBL561569	-4.05	-3.47	-3.52	-3.38
96	CHEMBL561570	-3.17	-3.51	-3.48	-3.20
97	CHEMBL561573	-3.80	-3.42	-3.55	-3.62
98	CHEMBL561574	-3.52	-3.84	-3.81	-3.60
99	CHEMBL561648	-3.11	-3.81	-3.73	-3.75
100	CHEMBL561649	-2.73	-3.12	-2.71	-2.86
101	CHEMBL561650	-3.43	-3.62	-3.52	-3.80
102	CHEMBL561653	-3.82	-3.36	-3.53	-3.78
103	CHEMBL561850	-2.81	-2.98	-2.81	-3.00
104	CHEMBL561975	-3.11	-3.46	-3.39	-3.57
105	CHEMBL562173	-2.92	-3.39	-3.43	-3.42
106	CHEMBL562250	-2.78	-3.48	-3.14	-3.39
107	CHEMBL562251	-3.36	-3.39	-3.49	-3.40

108	CHEMBL562507	-3.30	-3.57	-3.40	-3.60
109	CHEMBL563032	-3.33	-3.24	-3.09	-3.26
110	CHEMBL563154	-2.88	-3.49	-3.75	-3.31
111	CHEMBL563175	-3.17	-3.47	-3.46	-3.57
112	CHEMBL563181	-2.71	-3.39	-3.35	-3.36
113	CHEMBL563566	-3.14	-4.02	-4.12	-3.81
114	CHEMBL563804	-3.00	-3.45	-3.25	-3.43
115	CHEMBL563816	-3.33	-3.47	-3.58	-3.47
116	CHEMBL563871	-3.07	-4.05	-3.94	-3.80
117	CHEMBL563943	-3.01	-3.46	-3.67	-3.63
118	CHEMBL564112	-3.62	-3.68	-3.72	-3.57
119	CHEMBL564464	-3.37	-3.84	-3.82	-3.68
120	CHEMBL565150	-3.57	-3.55	-3.49	-3.50
121	CHEMBL569845	-2.80	-3.74	-3.53	-3.73
122	CHEMBL570307	-2.87	-3.33	-3.30	-3.21
123	CHEMBL570776	-3.06	-3.50	-3.33	-3.51
124	CHEMBL571226	-2.86	-4.07	-3.87	-4.06
125	CHEMBL572341	-3.59	-3.46	-3.63	-3.45
126	CHEMBL572342	-3.11	-3.22	-2.95	-3.28
127	CHEMBL572348	-2.99	-3.42	-3.35	-3.31
128	CHEMBL1294	-5.48	-5.41	-5.54	-5.46
129	CHEMBL24	-7.52	-5.64	-5.60	-5.75
130	CHEMBL3221410	-6.24	-5.79	-6.07	-5.99
131	CHEMBL3342578	-5.91	-6.05	-5.90	-6.13
132	CHEMBL3403637	-6.23	-6.04	-6.21	-6.40
133	CHEMBL3425512	-7.05	-6.93	-7.07	-6.82
134	CHEMBL3425617	-6.17	-5.97	-5.83	-5.77
135	CHEMBL3425618	-5.64	-5.92	-6.05	-5.74
136	CHEMBL3425619	-6.96	-6.06	-5.92	-6.05
137	CHEMBL3425620	-5.48	-5.98	-6.14	-5.95
138	CHEMBL3425621	-6.72	-6.31	-6.61	-6.30
139	CHEMBL3425622	-6.55	-5.98	-5.98	-6.00
140	CHEMBL3425623	-5.60	-5.96	-6.16	-6.01
141	CHEMBL3425624	-5.78	-6.06	-6.11	-6.01
142	CHEMBL3425625	-6.39	-6.28	-6.90	-6.26
143	CHEMBL3425626	-8.00	-7.13	-7.26	-7.25
144	CHEMBL3425627	-7.16	-7.27	-7.18	-7.28

145	CHEMBL3425628	-7.70	-7.09	-7.05	-7.26
146	CHEMBL3425629	-8.00	-7.21	-7.19	-7.29
147	CHEMBL3425630	-8.00	-7.14	-7.32	-7.32
148	CHEMBL3425631	-7.52	-7.24	-7.12	-7.34
149	CHEMBL3425632	-6.46	-6.95	-7.02	-6.62
150	CHEMBL3425633	-6.80	-7.21	-7.44	-7.05
151	CHEMBL3425634	-7.16	-6.90	-6.80	-6.74
152	CHEMBL3425635	-6.03	-6.66	-6.44	-6.40
153	CHEMBL3425636	-7.52	-6.91	-6.64	-6.67
154	CHEMBL3425637	-6.85	-6.78	-6.83	-6.81
155	CHEMBL3425639	-7.00	-6.37	-6.53	-6.34
156	CHEMBL3425640	-6.39	-6.18	-6.11	-6.06
157	CHEMBL3425642	-8.00	-7.33	-7.67	-7.89
158	CHEMBL3425643	-6.43	-6.55	-6.58	-6.60
159	CHEMBL3425644	-8.00	-6.87	-7.24	-7.21
160	CHEMBL3425646	-7.40	-7.73	-7.70	-7.74
161	CHEMBL3425647	-8.00	-7.46	-7.96	-7.73
162	CHEMBL3425648	-7.70	-7.92	-7.85	-8.05
163	CHEMBL3425649	-8.00	-7.83	-7.65	-7.63
164	CHEMBL3425650	-7.70	-8.21	-8.08	-8.02
165	CHEMBL3425653	-7.70	-8.22	-8.13	-8.23
166	CHEMBL3425654	-8.00	-8.17	-7.94	-8.09
167	CHEMBL3427784	-5.79	-6.08	-6.03	-6.04
168	CHEMBL3427785	-7.05	-6.33	-6.42	-6.42
169	CHEMBL3427786	-6.96	-6.07	-6.02	-6.18
170	CHEMBL3427787	-5.46	-6.11	-6.12	-6.22
171	CHEMBL3427788	-6.11	-6.02	-6.02	-5.95
172	CHEMBL3427789	-6.12	-6.10	-6.03	-6.10
173	CHEMBL3427790	-5.89	-6.29	-6.26	-6.24
174	CHEMBL3427791	-6.02	-5.93	-5.93	-5.80
175	CHEMBL3427792	-5.82	-6.14	-6.20	-6.16
176	CHEMBL3427793	-5.88	-5.97	-6.18	-6.02
177	CHEMBL3427794	-5.42	-5.90	-6.06	-5.88
178	CHEMBL3427795	-5.96	-6.23	-6.41	-6.15
179	CHEMBL3427796	-5.73	-5.73	-5.97	-5.77
180	CHEMBL3427797	-7.00	-5.73	-6.01	-5.79
181	CHEMBL3427798	-5.82	-5.77	-5.98	-5.83

182	CHEMBL421362	-7.10	-6.91	-6.96	-6.89
183	CHEMBL121	-5.12	-4.73	-5.02	-4.67
184	CHEMBL2216774	-5.04	-5.32	-5.36	-5.57
185	CHEMBL2216778	-6.85	-6.27	-6.15	-6.27
186	CHEMBL2216779	-5.56	-5.96	-5.79	-5.84
187	CHEMBL3431460	-5.84	-5.66	-5.68	-5.51
188	CHEMBL3431462	-5.30	-5.40	-5.36	-5.30
189	CHEMBL3431464	-5.23	-6.11	-5.32	-5.70
190	CHEMBL3431465	-5.40	-4.99	-5.02	-5.13
191	CHEMBL3431466	-5.25	-5.38	-5.20	-5.41
192	CHEMBL3431467	-5.36	-5.00	-4.94	-4.99
193	CHEMBL3431468	-5.78	-5.20	-5.16	-5.17
194	CHEMBL3431469	-5.41	-5.06	-4.91	-5.23
195	CHEMBL3431470	-5.53	-6.05	-5.94	-5.68
196	CHEMBL3431471	-5.52	-5.71	-5.82	-5.74
197	CHEMBL3431473	-6.02	-4.76	-4.55	-4.84
198	CHEMBL3431474	-5.46	-4.89	-4.92	-5.20
199	CHEMBL3431476	-5.65	-4.81	-4.85	-4.82
200	CHEMBL3431477	-6.15	-5.94	-6.19	-6.41
201	CHEMBL3431478	-6.17	-6.16	-6.32	-6.38
202	CHEMBL3431479	-6.31	-5.62	-5.50	-5.60
203	CHEMBL3431480	-5.45	-5.55	-5.17	-5.28
204	CHEMBL3431481	-5.65	-4.84	-5.35	-5.86
205	CHEMBL3431482	-5.77	-5.46	-5.73	-5.71
206	CHEMBL3431483	-5.78	-5.59	-5.36	-5.51
207	CHEMBL3431484	-5.81	-4.70	-4.63	-5.06
208	CHEMBL3431485	-5.38	-5.00	-4.97	-4.99
209	CHEMBL3431486	-5.53	-4.92	-5.08	-5.39
210	CHEMBL3431487	-5.29	-5.48	-5.49	-5.53
211	CHEMBL3431488	-5.62	-5.54	-5.54	-5.68
212	CHEMBL3431490	-5.66	-5.50	-5.31	-5.32
213	CHEMBL3431491	-5.62	-6.34	-5.53	-5.94
214	CHEMBL3431492	-5.27	-5.26	-5.05	-5.32
215	CHEMBL3431493	-5.35	-5.34	-5.23	-5.41
216	CHEMBL3431494	-5.44	-4.75	-5.50	-5.57
217	CHEMBL3431495	-5.67	-5.89	-5.84	-5.78
218	CHEMBL3431496	-5.48	-5.71	-5.69	-5.39

219	CHEMBL3431497	-5.36	-5.80	-5.91	-5.83
220	CHEMBL3431498	-5.55	-5.93	-5.79	-5.72
221	CHEMBL3431499	-5.54	-5.71	-5.89	-5.79
222	CHEMBL3431500	-5.55	-5.52	-5.66	-5.46
223	CHEMBL3431501	-6.13	-6.14	-6.42	-6.17
224	CHEMBL3431502	-5.35	-5.57	-5.48	-5.65
225	CHEMBL3431503	-5.52	-5.54	-5.35	-5.72
226	CHEMBL3431504	-5.72	-6.33	-6.40	-6.42
227	CHEMBL3431505	-5.90	-5.56	-5.64	-5.90
228	CHEMBL3431506	-5.74	-6.12	-6.05	-6.08
229	CHEMBL3431507	-5.71	-6.09	-5.88	-5.73
230	CHEMBL3431508	-5.46	-5.73	-5.59	-5.80
231	CHEMBL3431509	-5.53	-5.61	-5.66	-5.61
232	CHEMBL3431510	-5.75	-5.53	-5.81	-5.62
233	CHEMBL3431511	-5.55	-5.84	-5.80	-5.80
234	CHEMBL3431512	-5.61	-5.84	-5.84	-5.91
235	CHEMBL3431513	-7.70	-6.03	-6.03	-6.17
236	CHEMBL3431514	-7.56	-6.47	-7.04	-6.46
237	CHEMBL3431515	-5.29	-5.47	-5.44	-5.35
238	CHEMBL3431516	-5.79	-5.62	-5.76	-5.62
239	CHEMBL3431517	-5.72	-5.53	-5.28	-5.40
240	CHEMBL3431518	-5.44	-6.02	-5.53	-5.92
241	CHEMBL3431519	-5.83	-5.02	-5.49	-5.55
242	CHEMBL3431520	-6.08	-6.22	-5.47	-5.65
243	CHEMBL3431521	-5.65	-6.54	-6.17	-5.86
244	CHEMBL3431522	-5.80	-6.49	-6.53	-6.63
245	CHEMBL3431523	-6.02	-5.24	-5.36	-5.24
246	CHEMBL3431524	-5.59	-5.36	-5.22	-5.41
247	CHEMBL3431525	-5.64	-6.44	-6.11	-6.13
248	CHEMBL3431526	-5.23	-5.21	-5.12	-5.22
249	CHEMBL3431527	-6.58	-5.71	-5.75	-5.96
250	CHEMBL3431528	-5.27	-5.61	-5.38	-5.55
251	CHEMBL3431529	-5.54	-5.82	-5.78	-5.87
252	CHEMBL3431530	-5.59	-5.92	-5.70	-5.68
253	CHEMBL3431531	-5.65	-5.63	-5.59	-5.62
254	CHEMBL3431532	-5.50	-5.29	-6.39	-6.12
255	CHEMBL3431535	-5.50	-5.76	-5.60	-5.92

256	CHEMBL3431536	-5.57	-5.89	-6.01	-6.08
257	CHEMBL3431537	-5.61	-6.12	-5.46	-5.83
258	CHEMBL3431538	-6.34	-5.80	-6.28	-6.14
259	CHEMBL3431539	-5.64	-5.36	-5.04	-4.89
260	CHEMBL3431540	-5.58	-5.67	-5.41	-5.44
261	CHEMBL3431541	-6.13	-6.05	-6.52	-5.93
262	CHEMBL3431542	-5.55	-5.60	-5.61	-5.73
263	CHEMBL3431544	-6.03	-5.74	-5.20	-5.50
264	CHEMBL3431545	-5.48	-6.04	-5.83	-5.85
265	CHEMBL3431546	-5.70	-6.13	-5.87	-5.97
266	CHEMBL3431548	-5.78	-5.63	-5.91	-5.88
267	CHEMBL3431549	-5.92	-6.18	-5.80	-5.81
268	CHEMBL3431550	-5.72	-6.03	-5.56	-5.72
269	CHEMBL3431551	-5.79	-5.78	-5.50	-5.73
270	CHEMBL3431552	-5.75	-5.95	-5.67	-5.91
271	CHEMBL3431553	-5.32	-5.97	-5.84	-5.85
272	CHEMBL3431554	-5.67	-5.76	-5.46	-5.81
273	CHEMBL3431555	-6.57	-6.37	-6.45	-6.31
274	CHEMBL3431556	-5.82	-6.16	-6.54	-6.09
275	CHEMBL3431557	-5.47	-5.68	-5.47	-5.41
276	CHEMBL3431558	-5.56	-6.40	-5.92	-5.70
277	CHEMBL3431559	-5.32	-5.56	-5.56	-5.25
278	CHEMBL3431560	-5.43	-5.76	-5.95	-5.49
279	CHEMBL3431561	-5.63	-5.83	-6.29	-5.87
280	CHEMBL3431562	-7.16	-6.14	-5.93	-5.97
281	CHEMBL3431563	-5.81	-5.60	-5.54	-5.38
282	CHEMBL3431564	-8.00	-6.43	-6.27	-6.07
283	CHEMBL3431565	-5.79	-6.20	-6.20	-6.10
284	CHEMBL3431566	-5.42	-5.93	-5.70	-5.78
285	CHEMBL3431567	-5.70	-6.09	-6.21	-5.80
286	CHEMBL3431568	-5.26	-6.20	-6.06	-5.93
287	CHEMBL3431569	-6.29	-6.21	-5.90	-6.00
288	CHEMBL3431570	-5.48	-5.82	-5.15	-5.63
289	CHEMBL3431571	-5.53	-5.39	-5.45	-5.41
290	CHEMBL3431572	-5.28	-5.55	-5.51	-5.69
291	CHEMBL3431573	-5.77	-5.80	-5.96	-5.92
292	CHEMBL3431574	-5.84	-5.92	-5.89	-6.03

293	CHEMBL3431575	-6.59	-5.60	-5.71	-5.85
294	CHEMBL3431576	-6.09	-6.47	-6.10	-6.21
295	CHEMBL3431577	-6.24	-5.98	-6.04	-6.08
296	CHEMBL3431578	-5.54	-5.84	-5.87	-5.99
297	CHEMBL3431579	-5.59	-5.61	-5.60	-5.65
298	CHEMBL3431580	-5.91	-5.27	-5.92	-5.47
299	CHEMBL3431581	-5.59	-5.58	-5.51	-5.69
300	CHEMBL3431582	-5.65	-6.42	-6.38	-6.43
301	CHEMBL3431583	-5.70	-5.28	-5.34	-5.31
302	CHEMBL3431584	-6.28	-5.26	-5.04	-4.91
303	CHEMBL3431585	-5.53	-6.01	-5.95	-6.07
304	CHEMBL3431586	-5.87	-5.72	-5.68	-5.53
305	CHEMBL3431587	-5.78	-5.78	-6.01	-6.01
306	CHEMBL3431589	-6.18	-5.15	-4.86	-5.26
307	CHEMBL3431591	-6.19	-5.60	-5.57	-5.84
308	CHEMBL3431592	-5.27	-5.74	-5.47	-5.37
309	CHEMBL3431594	-5.56	-6.03	-5.97	-5.98
310	CHEMBL3431595	-5.72	-5.85	-5.89	-5.80
311	CHEMBL3431596	-5.54	-5.67	-5.94	-5.98
312	CHEMBL3431597	-5.70	-6.01	-5.43	-5.53
313	CHEMBL3431598	-5.26	-4.87	-5.03	-4.98
314	CHEMBL3431600	-5.77	-6.15	-6.01	-5.50
315	CHEMBL3431601	-5.52	-5.96	-6.40	-5.64
316	CHEMBL3431602	-5.78	-5.85	-5.59	-5.93
317	CHEMBL3431603	-5.66	-5.82	-5.89	-5.89
318	CHEMBL3431605	-5.70	-5.83	-5.51	-5.93
319	CHEMBL3431606	-5.82	-5.58	-5.51	-5.75
320	CHEMBL3431609	-5.36	-5.43	-5.50	-5.02
321	CHEMBL3431610	-6.64	-6.25	-6.06	-6.29
322	CHEMBL3431611	-5.52	-5.65	-5.88	-5.87
323	CHEMBL3431612	-5.67	-5.71	-5.85	-6.04
324	CHEMBL3431613	-5.60	-5.72	-6.08	-6.22
325	CHEMBL3431614	-5.54	-5.81	-6.05	-5.88
326	CHEMBL3431615	-5.20	-5.41	-5.14	-5.16
327	CHEMBL3431616	-5.42	-5.93	-5.77	-5.93
328	CHEMBL3431617	-5.26	-5.65	-5.82	-5.55
329	CHEMBL3431618	-5.38	-5.34	-5.32	-5.34

330	CHEMBL3431619	-5.50	-6.15	-6.06	-5.96
331	CHEMBL3431620	-5.27	-5.76	-5.98	-5.26
332	CHEMBL3431621	-5.55	-5.60	-5.63	-5.56
333	CHEMBL3431622	-5.65	-6.03	-5.92	-5.91
334	CHEMBL3431623	-5.29	-5.72	-5.65	-5.71
335	CHEMBL3431624	-5.44	-5.88	-5.78	-5.74
336	CHEMBL3431625	-5.95	-5.86	-5.99	-6.13
337	CHEMBL3431626	-5.39	-5.85	-5.94	-6.04
338	CHEMBL3431627	-5.49	-5.85	-5.75	-5.83
339	CHEMBL3431628	-5.91	-5.85	-6.20	-6.07
340	CHEMBL3431629	-6.22	-6.13	-5.98	-5.85
341	CHEMBL3431630	-5.84	-5.82	-5.99	-5.90
342	CHEMBL3431631	-5.43	-5.60	-5.51	-5.76
343	CHEMBL3431632	-6.02	-5.84	-6.13	-5.80
344	CHEMBL3431633	-6.03	-6.04	-6.21	-5.97
345	CHEMBL3431635	-5.73	-5.82	-5.76	-5.78
346	CHEMBL3431636	-6.40	-5.40	-5.40	-5.31
347	CHEMBL3431637	-5.36	-4.99	-4.83	-4.63
348	CHEMBL3431638	-5.79	-5.80	-5.84	-5.75
349	CHEMBL3431639	-5.76	-5.65	-5.47	-5.46
350	CHEMBL3431640	-5.53	-5.65	-5.59	-5.68
351	CHEMBL3431641	-6.89	-6.18	-6.22	-6.37
352	CHEMBL3431642	-6.00	-6.36	-6.03	-6.11
353	CHEMBL3431643	-5.45	-5.77	-5.53	-5.75
354	CHEMBL3431644	-5.75	-5.75	-6.00	-5.81
355	CHEMBL3431645	-5.27	-6.09	-6.08	-6.32
356	CHEMBL3431646	-5.82	-5.80	-5.93	-5.57
357	CHEMBL3431647	-5.67	-5.63	-5.60	-5.76
358	CHEMBL3431648	-5.86	-5.44	-5.08	-5.26
359	CHEMBL3431649	-5.88	-5.79	-5.38	-5.57
360	CHEMBL3431650	-5.27	-5.95	-5.39	-5.23
361	CHEMBL3431651	-5.21	-5.98	-6.28	-6.08
362	CHEMBL3431652	-6.52	-5.67	-5.44	-5.57
363	CHEMBL3431653	-6.33	-6.24	-5.69	-5.98
364	CHEMBL3431654	-5.20	-5.74	-5.67	-5.32
365	CHEMBL3431655	-5.41	-5.76	-5.59	-5.38
366	CHEMBL3431659	-6.92	-6.58	-6.48	-6.25

367	CHEMBL3431660	-5.74	-6.09	-5.96	-6.22
368	CHEMBL3431661	-5.48	-5.38	-5.61	-5.57
369	CHEMBL3431662	-6.07	-6.19	-6.44	-6.21
370	CHEMBL3431663	-6.02	-5.92	-5.43	-5.74
371	CHEMBL3431664	-5.37	-5.97	-5.95	-5.83
372	CHEMBL3431665	-5.26	-5.56	-5.26	-5.58
373	CHEMBL3431666	-5.07	-5.58	-5.24	-5.23
374	CHEMBL3431667	-5.19	-5.58	-5.48	-5.21
375	CHEMBL3431668	-5.96	-6.15	-6.15	-6.04
376	CHEMBL3431669	-5.87	-5.92	-5.62	-5.82
377	CHEMBL3431670	-5.31	-5.53	-5.44	-5.61
378	CHEMBL3431671	-5.77	-5.88	-5.58	-5.79
379	CHEMBL3431672	-5.77	-5.89	-5.84	-5.80
380	CHEMBL3431673	-5.57	-5.82	-5.73	-5.79
381	CHEMBL3431674	-5.87	-5.44	-5.67	-5.59
382	CHEMBL3431675	-5.66	-5.39	-5.51	-5.48
383	CHEMBL3431677	-5.51	-5.58	-5.75	-5.63
384	CHEMBL3431678	-5.54	-5.88	-5.73	-5.81
385	CHEMBL3431679	-5.50	-5.62	-5.67	-5.65
386	CHEMBL3431681	-5.70	-5.84	-6.04	-5.86
387	CHEMBL3431682	-5.55	-5.76	-5.94	-5.76
388	CHEMBL3431683	-5.40	-5.81	-5.70	-5.82
389	CHEMBL3431685	-6.02	-6.30	-6.58	-6.62
390	CHEMBL3431686	-5.72	-5.47	-5.81	-5.44
391	CHEMBL3431687	-5.65	-5.81	-5.98	-5.64
392	CHEMBL3431688	-5.39	-5.81	-5.85	-5.80
393	CHEMBL3431689	-5.66	-5.70	-5.62	-5.81
394	CHEMBL3431690	-5.39	-5.78	-5.78	-5.76
395	CHEMBL3431691	-5.44	-5.84	-5.81	-5.91
396	CHEMBL3431692	-5.38	-5.23	-5.09	-5.17
397	CHEMBL3431693	-5.15	-5.29	-5.47	-5.11
398	CHEMBL3431694	-5.04	-5.13	-5.24	-4.90
399	CHEMBL3431695	-4.94	-5.70	-5.71	-5.57
400	CHEMBL3431697	-5.36	-4.87	-4.92	-4.97
401	CHEMBL3431698	-5.29	-5.26	-4.82	-5.30
402	CHEMBL3431699	-5.57	-5.36	-5.59	-5.50
403	CHEMBL3431700	-5.32	-5.37	-5.28	-5.31

404	CHEMBL3431701	-5.84	-5.41	-5.37	-5.48
405	CHEMBL3431702	-5.77	-5.51	-5.44	-5.23
406	CHEMBL3431703	-5.61	-4.81	-4.71	-5.05
407	CHEMBL3431704	-7.52	-6.03	-6.17	-6.22
408	CHEMBL3431705	-6.22	-5.34	-5.21	-5.44
409	CHEMBL3431706	-5.39	-6.03	-6.28	-5.84
410	CHEMBL3431707	-5.96	-5.94	-5.66	-6.04
411	CHEMBL3431708	-5.64	-6.10	-6.21	-6.08
412	CHEMBL3431709	-5.57	-5.58	-5.91	-5.84
413	CHEMBL3431710	-5.53	-5.63	-6.05	-5.93
414	CHEMBL3431711	-6.29	-5.88	-6.04	-5.82
415	CHEMBL3431712	-6.07	-5.72	-5.79	-5.96
416	CHEMBL3431713	-5.46	-5.52	-5.50	-5.84
417	CHEMBL3431714	-5.47	-5.43	-5.44	-5.62
418	CHEMBL3431715	-6.00	-5.12	-5.12	-5.03
419	CHEMBL3431716	-5.63	-5.40	-5.35	-5.45
420	CHEMBL3431717	-5.68	-5.47	-5.11	-5.46
421	CHEMBL3431718	-5.50	-5.52	-5.46	-5.71
422	CHEMBL3431719	-5.73	-5.67	-5.67	-5.74
423	CHEMBL3431720	-6.33	-5.88	-6.03	-6.11
424	CHEMBL3431721	-5.56	-5.78	-5.55	-5.53
425	CHEMBL3431722	-6.11	-5.53	-5.65	-5.78
426	CHEMBL3431724	-7.40	-7.17	-6.66	-6.56
427	CHEMBL3431725	-7.16	-7.75	-7.16	-7.76
428	CHEMBL3431726	-5.83	-5.43	-5.14	-5.56
429	CHEMBL3431727	-6.31	-5.93	-5.90	-5.89
430	CHEMBL3431728	-6.37	-5.57	-5.92	-5.63
431	CHEMBL3431729	-5.72	-5.15	-5.04	-5.25
432	CHEMBL3431731	-5.26	-5.20	-5.03	-5.33
433	CHEMBL3431732	-5.35	-5.91	-5.83	-5.91
434	CHEMBL3431734	-5.50	-5.17	-5.21	-5.49
435	CHEMBL3431735	-5.23	-5.46	-5.35	-5.41
436	CHEMBL3431736	-5.26	-5.41	-5.77	-5.71
437	CHEMBL3431737	-5.35	-4.83	-5.16	-5.28
438	CHEMBL3431738	-5.57	-5.57	-5.29	-4.97
439	CHEMBL3431739	-5.33	-4.85	-5.09	-5.06
440	CHEMBL3431740	-5.77	-5.52	-5.49	-5.77

441	CHEMBL3431741	-5.13	-5.64	-6.09	-6.19
442	CHEMBL3431742	-5.09	-5.31	-5.51	-5.30
443	CHEMBL3431743	-5.37	-6.23	-6.00	-6.12
444	CHEMBL3431744	-5.03	-5.51	-5.67	-5.47
445	CHEMBL3431745	-5.23	-5.86	-5.90	-5.85
446	CHEMBL3431746	-5.17	-4.91	-4.99	-4.94
447	CHEMBL3431747	-5.19	-5.32	-5.03	-5.18
448	CHEMBL3431748	-5.22	-5.81	-5.58	-5.69
449	CHEMBL3431749	-5.02	-5.16	-5.15	-5.46
450	CHEMBL3431750	-5.16	-5.76	-5.62	-5.72
451	CHEMBL3431751	-5.45	-5.12	-4.93	-4.89
452	CHEMBL3431752	-5.65	-5.41	-5.57	-5.68
453	CHEMBL3431753	-5.54	-5.83	-6.00	-5.87
454	CHEMBL3431754	-5.66	-5.70	-5.54	-5.51
455	CHEMBL3431756	-5.98	-6.20	-6.16	-6.04
456	CHEMBL3431757	-5.66	-5.77	-5.54	-5.65
457	CHEMBL3431758	-4.61	-5.27	-5.40	-5.05
458	CHEMBL3431759	-5.41	-5.50	-5.43	-5.58
459	CHEMBL3431760	-5.42	-5.38	-5.57	-5.54
460	CHEMBL3431761	-5.31	-5.70	-5.80	-5.95
461	CHEMBL3431762	-5.64	-5.99	-6.19	-6.05
462	CHEMBL3431763	-6.68	-6.33	-6.41	-6.06
463	CHEMBL3431764	-5.59	-5.76	-5.60	-5.72
464	CHEMBL3431766	-5.76	-6.10	-5.79	-6.04
465	CHEMBL3431767	-7.22	-6.17	-6.23	-6.08
466	CHEMBL3431768	-5.82	-5.37	-5.74	-5.56
467	CHEMBL3431769	-6.08	-5.43	-5.72	-5.61
468	CHEMBL3431770	-6.28	-6.37	-6.43	-6.44
469	CHEMBL3431773	-5.61	-5.82	-5.82	-5.97
470	CHEMBL3431779	-5.94	-6.02	-6.18	-6.04
471	CHEMBL3431781	-5.88	-5.95	-6.09	-5.73
472	CHEMBL3431782	-5.72	-5.98	-6.17	-6.25
473	CHEMBL3431783	-5.65	-6.20	-6.17	-6.31
474	CHEMBL3431784	-6.42	-6.33	-6.32	-6.33
475	CHEMBL3431785	-7.22	-6.47	-6.60	-6.48
476	CHEMBL3431788	-6.07	-6.24	-6.07	-6.50
477	CHEMBL3431789	-5.71	-6.08	-6.16	-6.12

478	CHEMBL3431790	-5.72	-5.77	-5.68	-5.64
479	CHEMBL3431792	-6.41	-6.00	-6.01	-5.99
480	CHEMBL3431793	-5.75	-5.55	-5.62	-5.58
481	CHEMBL3431794	-5.55	-5.83	-5.60	-5.69
482	CHEMBL3431795	-6.55	-6.34	-6.26	-6.44
483	CHEMBL3431796	-6.31	-6.59	-6.29	-6.07
484	CHEMBL3431797	-5.99	-5.79	-5.65	-5.47
485	CHEMBL3431798	-5.62	-5.51	-5.67	-5.47
486	CHEMBL3431799	-5.45	-4.94	-5.20	-4.56
487	CHEMBL3431800	-5.08	-5.24	-5.49	-5.54
488	CHEMBL3431802	-4.94	-5.27	-5.44	-5.06
489	CHEMBL3431804	-5.83	-5.31	-4.90	-5.14
490	CHEMBL3431805	-5.29	-5.81	-5.72	-5.79
491	CHEMBL3431806	-5.30	-5.90	-5.84	-5.96
492	CHEMBL3431807	-5.45	-5.84	-5.94	-5.96
493	CHEMBL3431808	-5.42	-5.47	-5.62	-5.49
494	CHEMBL3431810	-5.42	-5.72	-5.36	-5.64
495	CHEMBL3431811	-5.91	-5.83	-5.72	-5.68
496	CHEMBL3431812	-7.22	-5.92	-6.26	-6.31
497	CHEMBL3431814	-6.52	-5.94	-6.09	-6.18
498	CHEMBL3431816	-6.41	-6.10	-6.22	-6.40
499	CHEMBL3431818	-5.50	-5.69	-5.87	-5.73
500	CHEMBL3431819	-5.51	-5.66	-5.70	-5.73
501	CHEMBL3431820	-5.69	-5.77	-5.52	-5.71
502	CHEMBL3431821	-5.46	-5.69	-5.81	-5.89
503	CHEMBL3431822	-5.77	-5.72	-5.34	-5.56
504	CHEMBL3431823	-6.00	-5.82	-5.99	-5.65
505	CHEMBL3431824	-7.52	-6.90	-6.74	-6.72
506	CHEMBL3431825	-5.61	-5.99	-5.95	-5.77
507	CHEMBL3431826	-5.60	-5.53	-5.78	-6.01
508	CHEMBL3431827	-5.89	-5.65	-5.76	-5.80
509	CHEMBL3431829	-5.69	-6.04	-6.14	-5.99
510	CHEMBL3431830	-6.57	-5.61	-6.06	-5.75
511	CHEMBL3431831	-7.22	-6.39	-6.66	-6.66
512	CHEMBL3431832	-7.22	-5.99	-6.08	-5.96
513	CHEMBL3431835	-6.64	-6.76	-6.81	-6.68
514	CHEMBL3431837	-6.17	-6.40	-6.10	-6.48

515	CHEMBL3431838	-7.05	-6.40	-6.85	-6.69
516	CHEMBL3431841	-8.00	-6.29	-6.71	-6.58
517	CHEMBL3431842	-6.04	-6.22	-6.33	-6.51
518	CHEMBL3431843	-7.52	-6.49	-6.54	-6.64
519	CHEMBL3431844	-6.12	-5.92	-6.12	-6.33
520	CHEMBL3431845	-5.74	-6.01	-6.17	-6.15
521	CHEMBL3431846	-5.28	-5.24	-4.87	-4.83
522	CHEMBL3431847	-5.63	-5.78	-5.66	-5.83
523	CHEMBL3431848	-5.68	-5.14	-5.42	-5.58
524	CHEMBL3431854	-5.71	-5.53	-5.34	-5.65
525	CHEMBL3431856	-5.90	-5.55	-5.31	-5.52
526	CHEMBL3431859	-5.26	-5.72	-5.74	-5.71
527	CHEMBL3431860	-5.91	-5.70	-6.04	-5.77
528	CHEMBL3431864	-5.88	-5.65	-5.55	-5.66
529	CHEMBL3431868	-5.68	-5.37	-5.35	-5.51
530	CHEMBL3431872	-6.52	-5.55	-5.44	-5.54
531	CHEMBL3431873	-5.32	-5.45	-5.05	-5.09
532	CHEMBL3431878	-5.21	-5.74	-5.38	-5.79
533	CHEMBL3431880	-5.65	-5.66	-5.77	-5.62
534	CHEMBL3431881	-6.89	-6.59	-7.19	-6.79
535	CHEMBL3431882	-5.59	-5.86	-5.71	-5.93
536	CHEMBL3431883	-5.49	-5.64	-5.81	-5.83
537	CHEMBL3431884	-5.47	-5.86	-6.24	-5.74
538	CHEMBL3431886	-5.49	-5.52	-5.80	-5.52
539	CHEMBL3431888	-5.45	-5.64	-5.64	-5.53
540	CHEMBL3431889	-5.46	-5.50	-5.02	-5.06
541	CHEMBL3431890	-5.45	-5.36	-5.50	-5.39
542	CHEMBL3431895	-5.66	-5.77	-5.82	-5.85
543	CHEMBL3431896	-5.73	-6.02	-5.94	-5.67
544	CHEMBL3431897	-5.75	-5.98	-6.06	-6.17
545	CHEMBL3431898	-5.70	-6.00	-5.87	-5.81
546	CHEMBL3431899	-5.75	-5.68	-5.93	-5.96
547	CHEMBL3431900	-5.62	-5.99	-5.77	-5.73
548	CHEMBL3431901	-6.43	-5.67	-5.62	-5.65
549	CHEMBL3431902	-5.60	-5.78	-5.73	-5.85
550	CHEMBL3431903	-5.61	-5.57	-5.78	-5.87
551	CHEMBL3431904	-5.26	-5.11	-5.07	-5.29

552	CHEMBL3431905	-5.42	-5.36	-4.95	-5.03
553	CHEMBL3431906	-5.30	-5.80	-5.43	-5.64
554	CHEMBL3431907	-6.75	-6.47	-6.58	-6.38
555	CHEMBL3431908	-6.28	-6.27	-6.46	-6.23
556	CHEMBL3431909	-6.33	-6.42	-6.44	-6.47
557	CHEMBL3431910	-5.52	-6.17	-6.35	-6.43
558	CHEMBL3431911	-5.29	-5.74	-6.04	-5.47
559	CHEMBL3431912	-5.78	-5.68	-6.10	-6.11
560	CHEMBL3431913	-5.84	-5.95	-6.07	-6.07
561	CHEMBL3431914	-5.66	-5.67	-5.94	-5.99
562	CHEMBL3431915	-5.78	-5.69	-5.70	-5.66
563	CHEMBL3431916	-5.73	-5.59	-5.55	-5.72
564	CHEMBL3431917	-5.41	-6.05	-5.88	-5.80
565	CHEMBL3431918	-5.58	-6.06	-6.15	-6.36
566	CHEMBL3431920	-5.45	-5.37	-5.40	-5.34
567	CHEMBL3431922	-5.94	-5.42	-5.51	-5.27
568	CHEMBL3431926	-5.51	-5.60	-5.61	-5.73
569	CHEMBL3431927	-7.52	-6.73	-6.52	-6.55
570	CHEMBL595	-5.13	-4.54	-4.44	-4.39
571	CHEMBL108877	-4.47	-4.39	-4.64	-4.50
572	CHEMBL11	-4.54	-4.11	-4.14	-4.23
573	CHEMBL110739	-4.67	-4.32	-4.29	-4.21
574	CHEMBL112	-5.91	-5.49	-5.48	-5.54
575	CHEMBL113	-5.21	-5.24	-4.97	-5.16
576	CHEMBL1143	-4.58	-4.69	-4.94	-4.82
577	CHEMBL13	-5.01	-4.74	-4.63	-4.77
578	CHEMBL130	-5.35	-5.23	-4.95	-5.32
579	CHEMBL131	-5.49	-5.15	-5.39	-5.21
580	CHEMBL134	-4.97	-5.46	-6.09	-5.66
581	CHEMBL13888	-4.56	-4.45	-4.41	-4.44
582	CHEMBL14060	-4.16	-4.11	-3.99	-3.93
583	CHEMBL14068	-4.35	-3.82	-4.02	-4.02
584	CHEMBL14092	-5.39	-4.45	-4.36	-4.58
585	CHEMBL14205	-4.05	-4.16	-4.10	-4.11
586	CHEMBL14282	-4.43	-3.65	-4.04	-3.94
587	CHEMBL15063	-4.40	-4.13	-4.26	-4.10
588	CHEMBL154	-5.29	-5.27	-5.58	-5.61

589	CHEMBL15844	-4.50	-4.65	-4.54	-4.53
590	CHEMBL15888	-4.12	-4.56	-4.61	-4.49
591	CHEMBL16	-4.58	-5.06	-5.38	-4.93
592	CHEMBL1790041	-5.87	-5.12	-5.18	-5.31
593	CHEMBL189558	-6.17	-6.38	-6.18	-6.33
594	CHEMBL190	-5.40	-5.37	-5.10	-5.33
595	CHEMBL191011	-5.04	-6.45	-6.60	-6.43
596	CHEMBL207225	-5.68	-5.20	-4.92	-4.96
597	CHEMBL22	-5.46	-4.87	-4.71	-5.01
598	CHEMBL226544	-6.02	-5.87	-6.19	-6.01
599	CHEMBL226545	-4.72	-4.94	-4.97	-4.98
600	CHEMBL226650	-4.57	-5.05	-4.84	-5.22
601	CHEMBL226651	-4.76	-4.76	-4.48	-4.90
602	CHEMBL226967	-4.80	-4.09	-4.57	-4.57
603	CHEMBL226968	-4.80	-5.18	-5.09	-5.17
604	CHEMBL226969	-4.76	-5.67	-5.28	-5.22
605	CHEMBL227020	-4.78	-4.51	-4.30	-4.37
606	CHEMBL227122	-4.89	-5.55	-5.36	-5.29
607	CHEMBL227123	-5.56	-5.49	-5.52	-5.22
608	CHEMBL227124	-6.38	-6.09	-6.41	-6.12
609	CHEMBL227173	-6.62	-6.14	-6.27	-6.32
610	CHEMBL227174	-6.18	-6.00	-6.19	-6.15
611	CHEMBL227181	-5.33	-4.27	-4.42	-4.27
612	CHEMBL227265	-5.77	-5.99	-5.68	-5.71
613	CHEMBL227338	-6.14	-5.93	-5.91	-6.07
614	CHEMBL227339	-6.54	-5.91	-5.91	-6.06
615	CHEMBL23	-5.62	-4.57	-5.13	-4.93
616	CHEMBL23832	-4.78	-5.40	-5.61	-5.42
617	CHEMBL266195	-4.70	-4.76	-4.53	-4.66
618	CHEMBL27	-4.53	-5.22	-5.05	-5.16
619	CHEMBL274009	-4.24	-3.93	-3.99	-4.06
620	CHEMBL277474	-5.36	-4.55	-4.27	-4.34
621	CHEMBL279564	-5.21	-3.75	-3.98	-4.53
622	CHEMBL288470	-4.55	-4.48	-4.12	-4.40
623	CHEMBL29878	-4.27	-4.66	-4.48	-4.54
624	CHEMBL323348	-4.18	-4.13	-4.05	-4.12
625	CHEMBL325415	-4.15	-4.38	-4.33	-4.26

626	CHEMBL35	-6.40	-6.32	-6.05	-6.56
627	CHEMBL354761	-4.77	-3.80	-3.95	-3.96
628	CHEMBL374395	-5.62	-5.63	-5.31	-5.43
629	CHEMBL379099	-5.21	-5.16	-5.17	-5.07
630	CHEMBL384467	-5.23	-5.03	-5.00	-4.87
631	CHEMBL386630	-4.59	-4.29	-4.62	-4.73
632	CHEMBL38688	-5.28	-4.82	-5.02	-5.01
633	CHEMBL387527	-6.10	-5.34	-5.34	-5.18
634	CHEMBL387744	-4.78	-5.35	-5.12	-5.29
635	CHEMBL388382	-6.92	-6.00	-6.01	-6.19
636	CHEMBL388384	-4.44	-3.96	-4.21	-4.33
637	CHEMBL388558	-4.66	-4.40	-4.57	-4.35
638	CHEMBL388559	-4.47	-4.47	-4.34	-4.08
639	CHEMBL388560	-5.02	-4.84	-5.03	-4.58
640	CHEMBL389621	-5.32	-4.75	-4.81	-4.77
641	CHEMBL389885	-4.19	-4.04	-4.16	-4.06
642	CHEMBL390937	-5.72	-5.90	-5.72	-5.67
643	CHEMBL390938	-5.22	-5.84	-5.87	-5.97
644	CHEMBL406819	-5.32	-4.81	-5.07	-4.98
645	CHEMBL424	-5.72	-4.68	-4.56	-4.44
646	CHEMBL429	-5.20	-5.39	-5.51	-5.37
647	CHEMBL435	-6.57	-5.92	-5.87	-6.09
648	CHEMBL46931	-4.03	-3.86	-3.93	-4.17
649	CHEMBL500	-5.49	-6.12	-5.89	-5.99
650	CHEMBL521	-4.49	-4.44	-4.56	-4.50
651	CHEMBL527	-4.88	-5.78	-5.03	-5.21
652	CHEMBL537	-5.20	-5.18	-5.38	-5.08
653	CHEMBL538	-3.92	-3.49	-3.82	-3.74
654	CHEMBL546	-4.90	-5.08	-4.87	-4.96
655	CHEMBL55772	-4.72	-5.06	-4.96	-5.08
656	CHEMBL571	-5.50	-4.57	-4.73	-4.76
657	CHEMBL642	-6.15	-5.13	-5.22	-5.38
658	CHEMBL6466	-4.22	-4.17	-3.95	-3.77
659	CHEMBL649	-5.60	-5.32	-5.00	-5.43
660	CHEMBL66381	-4.73	-4.90	-5.10	-5.20
661	CHEMBL6640	-5.27	-5.04	-4.96	-4.71
662	CHEMBL6966	-4.41	-4.24	-4.33	-4.33

663	CHEMBL6995	-6.13	-5.85	-6.13	-6.02
664	CHEMBL72	-4.45	-4.45	-4.52	-4.53
665	CHEMBL82411	-6.40	-5.26	-5.00	-5.05
666	CHEMBL9	-6.72	-6.40	-6.44	-6.42
667	CHEMBL9967	-6.06	-5.34	-5.23	-5.38
668	CHEMBL108877	-4.82	-4.36	-4.54	-4.75
669	CHEMBL11	-4.20	-4.16	-4.09	-4.23
670	CHEMBL110739	-4.47	-4.52	-4.32	-4.21
671	CHEMBL112	-5.65	-5.55	-5.51	-5.54
672	CHEMBL113	-4.80	-5.13	-5.15	-5.32
673	CHEMBL1143	-3.90	-4.69	-5.08	-5.08
674	CHEMBL13	-4.84	-4.74	-4.68	-4.74
675	CHEMBL130	-5.24	-5.42	-4.95	-5.50
676	CHEMBL131	-5.52	-5.16	-5.39	-5.21
677	CHEMBL134	-4.94	-5.77	-6.09	-5.59
678	CHEMBL13888	-4.16	-4.45	-4.39	-4.33
679	CHEMBL14060	-3.83	-4.06	-4.06	-3.98
680	CHEMBL14068	-3.71	-3.86	-4.03	-3.99
681	CHEMBL14092	-4.27	-4.45	-4.58	-4.63
682	CHEMBL14205	-3.66	-4.29	-4.10	-4.25
683	CHEMBL14282	-3.91	-3.65	-4.10	-4.16
684	CHEMBL15063	-4.14	-4.38	-4.61	-4.04
685	CHEMBL154	-5.26	-5.30	-5.46	-5.47
686	CHEMBL15888	-3.59	-4.53	-4.59	-4.53
687	CHEMBL16	-4.93	-5.22	-5.16	-4.88
688	CHEMBL1790041	-5.52	-5.50	-5.18	-5.31
689	CHEMBL190	-5.58	-5.44	-5.10	-5.33
690	CHEMBL22	-5.38	-4.93	-4.71	-4.93
691	CHEMBL226650	-3.88	-4.87	-4.97	-5.22
692	CHEMBL226651	-4.33	-4.76	-4.66	-5.02
693	CHEMBL226967	-4.36	-4.09	-4.53	-4.81
694	CHEMBL227181	-4.89	-4.22	-4.41	-4.29
695	CHEMBL23	-3.91	-4.80	-5.13	-5.01
696	CHEMBL23832	-4.61	-5.32	-5.74	-5.50
697	CHEMBL266195	-4.21	-4.71	-4.68	-4.66
698	CHEMBL27	-4.42	-5.04	-5.28	-5.14
699	CHEMBL274009	-3.64	-4.11	-4.02	-4.10

700	CHEMBL277474	-5.00	-4.77	-4.27	-4.47
701	CHEMBL279564	-4.32	-3.75	-3.98	-4.74
702	CHEMBL288470	-4.14	-4.48	-4.49	-4.40
703	CHEMBL29878	-3.58	-4.66	-4.55	-4.57
704	CHEMBL323348	-3.80	-4.09	-4.05	-4.27
705	CHEMBL325415	-3.58	-4.38	-4.37	-4.24
706	CHEMBL35	-6.24	-6.23	-6.33	-6.67
707	CHEMBL354761	-4.09	-3.85	-3.95	-3.96
708	CHEMBL384467	-4.95	-5.03	-4.75	-4.87
709	CHEMBL386630	-3.87	-4.31	-4.80	-4.73
710	CHEMBL38688	-4.82	-4.81	-5.07	-5.01
711	CHEMBL388384	-3.44	-4.22	-4.06	-4.33
712	CHEMBL388558	-4.33	-4.30	-4.57	-4.35
713	CHEMBL388559	-4.03	-4.60	-4.42	-4.29
714	CHEMBL388560	-4.51	-5.22	-5.18	-4.87
715	CHEMBL389621	-5.07	-4.91	-4.81	-4.79
716	CHEMBL389885	-3.61	-4.04	-4.25	-4.14
717	CHEMBL406819	-5.28	-4.81	-5.07	-5.29
718	CHEMBL424	-5.31	-4.64	-4.53	-4.35
719	CHEMBL429	-5.25	-5.39	-5.51	-5.37
720	CHEMBL435	-6.54	-5.79	-5.87	-6.19
721	CHEMBL46931	-3.53	-4.18	-4.00	-4.17
722	CHEMBL500	-5.85	-6.12	-6.06	-5.86
723	CHEMBL521	-4.14	-4.56	-4.56	-4.48
724	CHEMBL527	-4.72	-5.72	-5.39	-5.16
725	CHEMBL537	-5.34	-5.19	-5.38	-5.27
726	CHEMBL538	-3.52	-3.77	-3.82	-3.74
727	CHEMBL546	-5.03	-5.08	-4.87	-4.67
728	CHEMBL571	-5.40	-4.66	-4.73	-4.76
729	CHEMBL642	-5.58	-5.18	-5.27	-5.33
730	CHEMBL6466	-3.56	-4.17	-4.20	-3.77
731	CHEMBL649	-4.49	-5.59	-5.22	-5.81
732	CHEMBL66381	-4.65	-4.90	-5.12	-5.20
733	CHEMBL6966	-3.96	-4.26	-4.45	-4.55
734	CHEMBL6995	-6.43	-5.85	-5.93	-6.02
735	CHEMBL72	-3.81	-4.67	-4.43	-4.53
736	CHEMBL9	-6.75	-6.26	-6.32	-6.49

737	CHEMBL9967	-6.09	-5.51	-5.37	-5.44
738	BDH_33697220	-4.52	-5.27	-4.87	-5.39
739	BDH_33701281	-6.52	-6.28	-6.19	-6.07
740	BDH_33702300	-4.64	-5.15	-5.01	-5.16
741	LAS_51647569	-4.63	-5.33	-5.09	-5.35
742	LAS_51649260	-4.54	-4.68	-4.52	-4.40
743	LAS_51658240	-4.56	-4.51	-4.39	-4.35
744	LAS_51663301	-6.05	-5.91	-5.66	-5.60
745	LAS_51900482	-4.60	-4.64	-4.49	-4.53
746	LAS_51900724	-5.84	-5.47	-5.37	-5.60
747	LAS_51904053	-5.68	-5.41	-5.48	-4.87
748	LAS_52042692	-4.55	-5.29	-5.27	-5.47
749	LAS_52042848	-4.57	-4.39	-4.82	-4.77
750	LAS_52044571	-5.20	-5.31	-5.31	-5.49
751	LAS_52104235	-5.82	-6.18	-5.78	-6.07
752	LAS_52105209	-5.69	-5.05	-5.06	-5.09
753	LAS_52107944	-4.59	-5.04	-5.82	-5.59
754	LAS_52110813	-4.59	-5.39	-4.74	-5.03
755	LAS_52132208	-6.10	-5.65	-5.57	-5.94
756	LAS_52135120	-4.80	-4.90	-4.70	-4.82
757	LAS_52135200	-4.63	-4.52	-4.84	-4.78
758	LAS_52137850	-4.52	-4.21	-4.72	-4.31
759	LAS_52141146	-5.63	-4.76	-5.00	-4.50
760	LAS_52141152	-4.91	-3.99	-4.24	-4.83
761	LAS_52144084	-4.52	-4.29	-4.49	-4.71
762	LAS_52146431	-4.54	-4.77	-4.58	-4.93
763	LAS_52146600	-5.46	-4.93	-4.98	-4.63
764	LAS_52146805	-5.84	-5.61	-5.45	-5.24
765	LAS_52152793	-5.78	-4.80	-4.68	-4.62
766	LAS_52154828	-4.70	-4.92	-4.97	-5.03
767	LAS_52155805	-5.34	-5.62	-5.56	-5.68
768	LAS_52155968	-4.61	-4.89	-5.14	-4.84
769	LAS_52159132	-4.53	-4.86	-4.42	-4.99
770	LAS_52162509	-4.55	-4.47	-4.49	-4.24
771	LAS_52164169	-5.17	-6.38	-6.20	-6.38
772	LAS_52166734	-5.81	-6.23	-6.38	-6.38
773	LAS_52167560	-5.55	-5.90	-5.84	-6.11

774	LAS_52167954	-4.88	-5.48	-5.33	-5.56
775	LAS_52168541	-4.62	-4.66	-4.65	-4.55
776	LAS_52168719	-4.52	-4.50	-4.52	-4.26
777	LAS_52169928	-4.77	-4.90	-5.21	-4.89
778	LAS_52169970	-4.55	-5.50	-5.79	-5.74
779	LAS_52170106	-5.80	-5.82	-5.76	-5.56
780	LAS_52172509	-4.56	-6.12	-5.52	-5.21
781	LAS_52172754	-6.22	-5.60	-5.53	-5.67
782	LAS_52172839	-6.13	-5.30	-5.59	-5.51
783	LAS_52466348	-4.54	-4.58	-4.44	-4.52
784	LAS_52480202	-4.60	-4.81	-4.72	-4.58
785	LAS_52482819	-6.00	-5.05	-4.92	-4.93
786	LAS_52506402	-4.91	-4.60	-4.79	-5.00
787	LAS_52507024	-5.24	-5.01	-5.09	-5.04
788	LAS_52511076	-4.52	-4.69	-4.79	-5.14
789	LAS_52511151	-5.33	-4.60	-4.83	-4.99
790	LAS_52514101	-5.27	-4.62	-4.45	-4.32
791	LAS_52515343	-4.84	-4.96	-4.44	-4.70
792	LAS_52515710	-4.64	-5.36	-5.41	-5.55
793	LAS_52515717	-4.52	-4.08	-4.64	-4.13
794	AC_9A54	-7.00	-6.94	-6.76	-6.60
795	AC_9A5	-7.20	-7.47	-7.15	-6.66

Table S4 Dependence of elapsed CPU time on the number of conformers

No. of structures	Model A		Model B		Model AB		CPU time (min)	
	RMSE	Q^2	RMSE	Q^2	RMSE	Q^2	Average	Max
300	0.530	0.782	0.528	0.784	0.522	0.789	9.46	61.53
100	0.532	0.781	0.531	0.781	0.520	0.790	2.61	12.55
50	0.534	0.780	0.529	0.784	0.521	0.790	1.11	10.43
20	0.530	0.783	0.532	0.781	0.533	0.780	0.33	4.21
10	0.534	0.779	0.531	0.782	0.527	0.785	0.13	2.10
5	0.536	0.778	0.535	0.779	0.532	0.781	0.05	1.06
3	0.537	0.777	0.538	0.776	0.534	0.770	0.02	0.69
2	0.541	0.774	0.542	0.773	0.543	0.772	0.01	0.48
1	0.543	0.772	0.539	0.775	0.540	0.775	0.01	0.10

Table S5 Coefficients {*c*} obtained by the L1 regularization instead of the L2 regularization in Equation 16.

No.	Model AB	{c}	Model A	{c}	Model B	{c}
1	No. aromatic atoms	1.338	No. of C=N	0.805	No. aromatic atoms	1.681
2	<A>$_{wat}$ of sp$_2$C	1.248	Log<Rx>$_{wat}$	0.649	<A>$_{wat}$ of sp$_2$C	1.514
3	<R>$_{mem}$	1.002	<A>wat of cationic C	0.564	No. of C=N	0.910
4	Log<Rx>$_{mem}$	0.954	<R>$_{wat}$	0.556	<A>$_{wat}$ of cationic C	0.507
5	<Rx>$_{mem}$	0.788	No. atoms in rings	0.506	No. of NH	0.435
6	<Rz>$_{mem}$	0.720	No. of NH	0.389	<R>$_{mem}$	0.418
7	No. of C=N	0.667	No. of N atoms	0.347	<R>$^2_{mem}$	0.396
8	<R>$_{wat}$	0.651	No. of aromatic atoms	0.326	No. atoms in rings	0.396
9	Log<Rz>$_{mem}$	0.544	No. of -CH$_2$-	0.294	No. of -CH$_2$-	0.282
10	No. atoms in rings	0.479	<Rz>$_{wat}$	0.282	No. of CNO	0.281
11	No. of NH	0.431	No. of -CH$_2$-CH$_2$-	0.257	No. of C-N-C	0.242
12	No. of N atoms	0.359	No. of CNO	0.243	No. of -CH$_2$-CH$_2$-	0.230
13	No. of -CH$_2$-	0.329	No. of C-N-C	0.228	<Ry>$_{mem}$	0.229
14	No. of aromatic atoms	0.318	No. of OH	0.206	No. of N atoms	0.228
15	<A>$_{wat}$ of cationic C	0.292	q(OH)	0.186	No. of C=O	0.221
16	<Rz>wat	0.283	No. of C=O	0.180	No. of OH	0.211
17	No. of CNO	0.222	No. N atoms in aromatic rin	0.152	<Rz>$_{mem}$	0.206
18	No. of OH	0.214	No. of O atoms in rings	0.151	q(OH)	0.194
19	No. of -CH$_2$-CH$_2$-	0.208	No. of -CH$_2$-CH$_2$-	0.145	No. of -CH$_2$-CH$_2$-	0.193
20	No. of OH	0.185	No. of -N-	0.138	No. N atoms in aromatic rin	0.175
21	<R>$^2_{wat}$	0.175	No. of -CH$_2$-CH$_2$- in rings	0.135	No. of -CH$_2$-CH$_2$- in rings	0.144
22	No. of C=O	0.154	No. of A(QH)-CH$_2$-A[a]	0.121	No. of -N-	0.139
23	No. of O atoms in rings	0.140	<R>$^2_{wat}$	0.110	No. of O atoms	0.138
24	<R>$^2_{mem}$	0.127	5-membered ring	0.108	No. of aromatic atoms	0.137
25	No. N atoms in aromatic rin	0.124	4-membered ring	0.100	No. of O atoms in rings	0.119
26	No. of -N-	0.120	No. of -CH$_2$-CH$_3$	0.099	No. of A(QH)-CH$_2$-A[a]	0.106
27	No. of C-N-C	0.118	<A>$_{wat}$ of sp$_2$ O	0.095	q(NH)	0.097
28	No. of -CH$_2$-CH$_2$- in rings	0.113	No. of -CH$_2$-	0.087	<A>$_{mem}$ of sp$_3$ O	0.092
29	4-membered ring	0.089	<A>$_{wat}$ of sp$_3$ O	0.086	No. of -CH$_2$-CH$_3$	0.083
30	q(OH)	0.088	No. of O atoms	0.083	<A>$_{wat}$ of O of COOH	0.074

[a]: Atoms “A” and “Q” are any atom and pseudo atom, respectively.

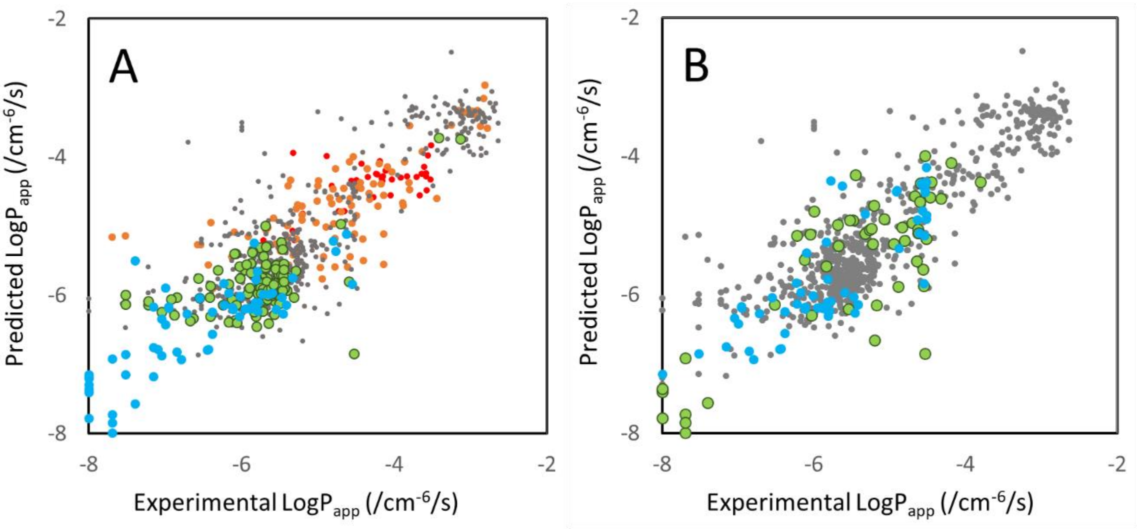

Figure S1. Predicted and experimental LogP_{app} obtained by the L1 regression model in terms of (A) *MW* and (B) N_{ring}. (A), respectively. The model used is Model B at N_{struct}=100. (A) Red, orange, gray, green, and blue spheres represent the molecules with 0 < *MW* < 150 Da, 150 Da < *MW* < 300 Da, 300 Da < *MW* < 500 Da, 500 Da < *MW* < 600 Da, and 600 Da < *MW*, respectively. (B) Gray, green, and blue spheres represent the molecules with N_{ring} C < 12, 12 < N_{ring} < 20, and 20 < N_{ring}, respectively.

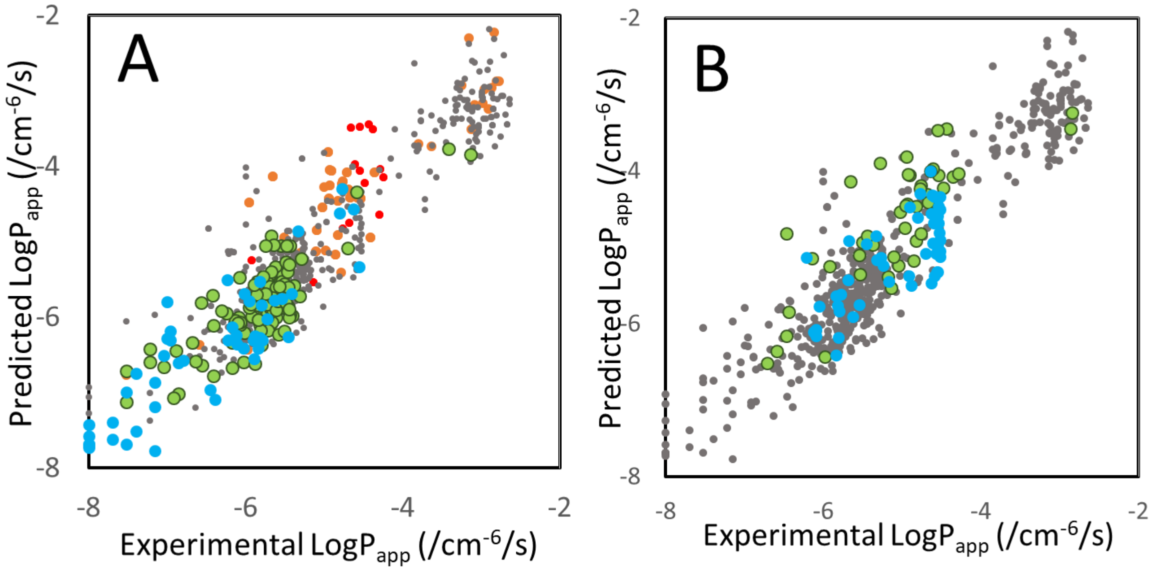

Figure S2. Predicted and experimental LogP_{app} obtained by the L2 regression model based on the Moldred descriptors in terms of (A) *MW* and (B) N_{ring}. (A), respectively. (A) Red, orange, gray, green, and blue spheres represent the molecules with 0 < *MW* < 150 Da, 150 Da < *MW* < 300 Da, 300 Da < *MW* < 500 Da, 500 Da < *MW* < 600 Da, and 600 Da < *MW*, respectively. (B) Gray, green, and blue spheres represent the molecules with N_{ring} C < 12, 12 < N_{ring} < 20, and 20 < N_{ring}, respectively.
